# Genome-wide analysis of growth phase-dependent translational and transcriptional regulation in halophilic archaea

**DOI:** 10.1186/1471-2164-8-415

**Published:** 2007-11-12

**Authors:** Christian Lange, Alexander Zaigler, Mathias Hammelmann, Jens Twellmeyer, Günter Raddatz, Stephan C Schuster, Dieter Oesterhelt, Jörg Soppa

**Affiliations:** 1 Institute for Molecular Biosciences, Johann Wolfgang Goethe University, Max-von-Laue-Strasse 9, 60438 Frankfurt a.M., Germany; 2 Department of Membrane Biochemistry, Max Planck Institute of Biochemistry, Am Klopferspitz 18, 82152 Martinsried, Germany; 3 Genomics Group, Max Planck Institute for Developmental Biology, Spemannstrasse 37-39, 72076 Tübingen, Germany; 4 GANYMED Pharmaceuticals AG, Freiligrathstrasse 12, 55131 Mainz, Germany; 5 MR-Center, Max Planck Institute for Biological Cybernetics, Spemannstrasse 34, 72076 Tübingen, Germany; 6 Center for Comparative Genomics and Bioinformatics, Center for Infectious Disease Dynamics, Pennsylvania State University, University Park, Pennsylvania 16802, USA

## Abstract

**Background:**

Differential expression of genes can be regulated on many different levels. Most global studies of gene regulation concentrate on transcript level regulation, and very few global analyses of differential translational efficiencies exist. The studies have revealed that in *Saccharomyces cerevisiae*, *Arabidopsis thaliana*, and human cell lines translational regulation plays a significant role. Additional species have not been investigated yet. Particularly, until now no global study of translational control with any prokaryotic species was available.

**Results:**

A global analysis of translational control was performed with two haloarchaeal model species, *Halobacterium salinarum *and *Haloferax volcanii*. To identify differentially regulated genes, exponentially growing and stationary phase cells were compared.

More than 20% of *H. salinarum *transcripts are translated with non-average efficiencies. By far the largest group is comprised of genes that are translated with above-average efficiency specifically in exponential phase, including genes for many ribosomal proteins, RNA polymerase subunits, enzymes, and chemotaxis proteins. Translation of 1% of all genes is specifically repressed in either of the two growth phases. For comparison, DNA microarrays were also used to identify differential transcriptional regulation in *H. salinarum*, and 17% of all genes were found to have non-average transcript levels in exponential versus stationary phase.

In *H. volcanii*, 12% of all genes are translated with non-average efficiencies. The overlap with *H. salinarum *is negligible. In contrast to *H. salinarum*, 4.6% of genes have non-average translational efficiency in both growth phases, and thus they might be regulated by other stimuli than growth phase.

**Conclusion:**

For the first time in any prokaryotic species it was shown that a significant fraction of genes is under differential translational control. Groups of genes with different regulatory patterns were discovered. However, neither the fractions nor the identity of regulated genes are conserved between *H. salinarum *and *H. volcanii*, indicating that prokaryotes as well as eukaryotes use differential translational control for the regulation of gene expression, but that the identity of regulated genes is not conserved.

For 70 *H. salinarum *genes potentiation of regulation was observed, but for the majority of regulated genes either transcriptional or translational regulation is employed.

## Background

The accurate regulation of the expression of genes into biological functions is essential for all living organisms. Coordinate regulation is necessary for the adaptation to different environmental conditions, for stress response, for development, for cell cycle control, and for many additional processes. Regulation can operate at various levels, from the initiation of transcription to posttranslational control of protein activity and beyond. While selected examples have been studied for decades, methods for the global analysis of gene expression have been developed more recently. Most widely used is the determination of transcript level changes using DNA microarrays. However, it has become more and more obvious that regulation of gene expression happens at all levels, and thus the post-transcriptional regulation should not be neglected in genome-wide studies of differential gene expression [1-3]. 2D gel electrophoresis is a well established global approach to analyze the steady state level of proteins in parallel. However, disadvantages are that only subproteomes can be studied (e.g. the cytoplasmic, membrane, or extracellular proteome), that the identity of every single spot has subsequently to be identified by mass spectrometry, and that proteins can be very stable and the presence of a protein does not necessarily mean that it is synthesized and needed under the conditions under investigation.

Therefore it would be better to determine translational efficiency more directly. One global approach was to predict the translational efficiencies of all *E. coli *genes using a neural network [4]. The bioinformatic analysis was based on the experimental determination of the reporter gene activities of 185 clones carrying randomized ribosome binding sites [5]. However, transcript levels were not determined but assumed to be identical in all cases. Another approach to quantify the protein production rate is the combination of pulse-labeling with 2D gel electrophoresis. Very few studies exist, but the protein production levels have not been correlated to the transcript levels [6,7] and thus translational efficiencies could not be calculated.

Global methods for the determination of translational efficiencies and identification of translationally regulated genes typically are comprised of density gradient centrifugation of a cytoplasmic extract, resulting in the separation of mRNAs according to their ribosome association, followed by the comparison of RNA fractions using DNA microarrays [3,8-11]. In a method called "ribosome density profiling", many fractions are collected and used to measure the exact ribosome density of each mRNA. The transcript levels in all fractions are compared to a common reference with DNA microarrays [12]. This method is extremely demanding in terms of costs, DNA microarrays, and time. An alternative is the comparison of combined fractions of a gradient containing either free, non-translated RNA or translated, polysome-bound RNAs [13]. For both approaches, the experiment has to be performed with two defined culture conditions to be able to discriminate mRNAs that are subject to differential regulation of translational efficiencies from mRNAs that have a constitutive low translation initiation efficiency. Most often, cultures are compared before and after the application of a stress condition, the addition of an inhibitor, etc.

While genome-wide studies of differential transcription and mRNA decay are already available for all three domains of life, global analyses of translational regulation have until now only been performed with three species of eukaryotes (*Saccharomyces cerevisiae*: [2,12-16], *Arabidopsis thaliana*: [17-20], human cell cultures: [11,21-23]). All these studies identified translationally regulated genes, their fractions varied from about 1% to 25%, in part exceeding and in part falling below the number of transcriptionally regulated genes. Some studies revealed that translational regulation was responsible for more than half of the detected protein synthesis rate changes [2], while other analyses rather indicated a specific translational inhibition of a defined set of genes containing specific regulatory elements [24].

While it is now well-established that a considerable fraction of eukaryotic genes is regulated on the translational level, it is generally assumed that the fraction of translationally regulated genes is much smaller in prokaryotes, which are thought to nearly exclusively use transcript level regulation. However, no global analysis of translational regulation was available for any prokaryotic species. Some specific genes have been characterized showing that translational regulation involving regulatory proteins or small non-coding RNAs does occur at least in bacteria [25-28].

Almost nothing is known about translational regulation in archaea. For some genes it has been found that the changes of mRNA levels and protein levels do not fit, and this had been taken as circumstantial evidence that translational regulation might exist also in the domain of archaea [29]. To clarify the occurrence and importance of translational regulation in archaea, genome-wide analyses of translational efficiencies were performed by comparing exponentially growing cells with stationary phase cells. Two haloarchaeal model species, *Halobacterium salinarum *and *Haloferax volcanii *[30], were used. This allowed to evaluate the evolutionary conservation of translational control in archaea. Furthermore, DNA microarrays were used to analyse growth phase-dependent transcript level regulation and determine whether the so-called potentiation of regulation (regulation of genes on both levels) exists in haloarchaea.

## Results and Discussion

### Experimental approach for the identification of translationally regulated genes in two species of different haloarchaeal genera

**Figure 1:**
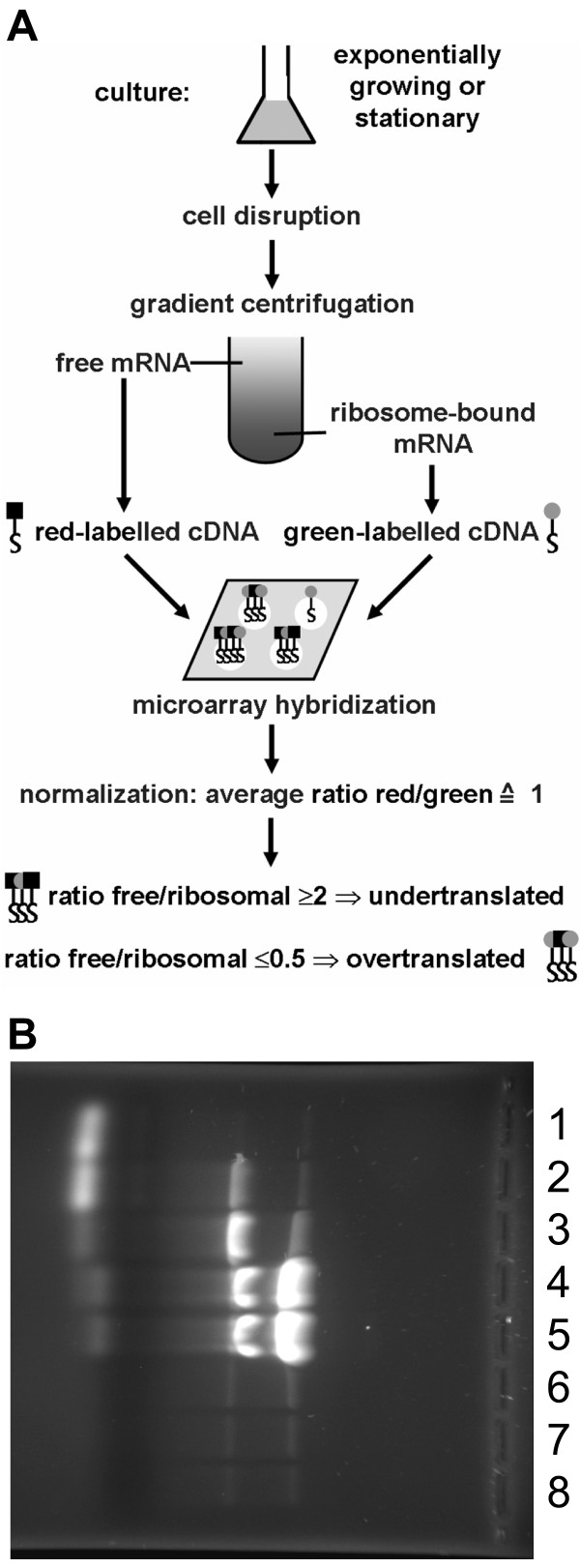
**Global analysis of translational regulation**. **A**. Schematic overview of the experimental approach. **B**. An agarose gel showing the RNAs contained in the fractions of a typical density gradient. The numbers 1 – 8 denote the fractions from top to bottom. Fractions 1 and 2 and fractions 6–8 were pooled to yield the fractions of "free transcripts" and "ribosome-bound transcripts", respectively.

Translationally regulated genes are characterized by differential translational efficiencies of their transcripts under different conditions. We chose to compare exponentially growing and stationary phase cells to identify translationally regulated genes. Exponentially growing cultures were defined to have a cell density of 4 – 5 × 10^8 ^cells/ml, about two to three cell divisions before they enter stationary phase. Stationary phase cultures were grown to the maximal cell density and incubated for another 24 hours before the cells were harvested. In both cases cytoplasmic extracts were fractionated using sucrose density gradients to separate free mRNA, which was not translated, from polysome-bound mRNA, which was actively translated at the time of cell disruption. Fig. 1A gives an overview of the experimental approach. Fig. 1B shows the RNAs that were present in the different fractions of a typical density gradient. The two top fractions and the bottom three fractions were pooled, respectively, and were used for RNA isolation, cDNA synthesis, labelling with different fluorescent dyes, and comparison using DNA microarrays. To ensure a high fidelity of results, at least three biological replicates were performed. All transcripts with an at least twofold deviation from the average value of all genes under only one of the two growth conditions were assumed to be translationally regulated. It should be noted that only "free" and "ribosome-bound" transcripts have been quantified, but that growth phase-dependent differences in the number of ribosomes on translated transcripts could not be resolved. This would have required to divide the gradient into a higher number of fractions and the individual analysis of all fractions with DNA microarrays ("ribosome density mapping"). The translational efficiency is typically determined by the translation initiation rate, but for transcripts with rare codons at their 5'-end can also be determined by the elongation rate [31]. Changes of one of these parameters without a concomitant alteration of the fraction of free transcripts would not have been noted. Therefore the results described below define the lower limit of translational regulation in haloarchaea.

Two different species, *H. salinarum *and *H. volcanii*, were used for the analysis, because we aimed to characterize the evolutionary conservation of translational regulation. All results have been deposited in the ArrayExpress database (accession numbers see Methods). For both species, the majority of protein-coding genes were found in the ribosome-bound fraction and thus were actively translated in the two growth phases, i.e. 70% of all *H. salinarum *genes and 65% of all *H. volcanii *genes. These values are similar to the results obtained with *Saccharomyces cerevisiae*. Up to 80% of all yeast genes were reported to be translated [32].

### Genes with differential translational repression in *H. salinarum*

In *H. salinarum*, 21 genes were found to be at least twofold undertranslated in one growth phase, but not in the other (Table 1). 14 of these genes exhibit a translational repression in exponential phase, while the translation of seven genes is repressed in stationary phase. The highest level of translational regulation was detected for the transcript encoding translation initiation factor aIF-1A (OE3470F), which has a 13-fold higher translational efficiency during exponential phase than during stationary phase. The initiation factor aIF-1A is conserved in all three domains of life and was shown to be essential both for *E. coli *and *S. cerevisiae *[33]. The eukaryotic homologue, eIF-1A, is important for ribosome-scanning and for initiation codon selection [34]. If the archaeal factor had the same function, a decrease of the aIF-1A concentration due to translational repression in stationary phase could be expected to lead to a general decrease in initiation rate at many or all transcripts. As the initiation step is rate limiting, this would lead to a general decrease in translational efficiency and thus potentiate the regulatory effect.

**Table 1:** *H. salinarum *genes with a differential translational repression in exponential phase or in stationary phase^*1^

		Translational repression factor						Transcript level		
		
ORF	Gene product	Exp			Stat			Stat/exp		
		
		∅	SD	n	∅	SD	n	∅	SD	n
OE4511R	Hypothetical protein	**9.0**	3.8	4	**1.2**	0.2	3	**0.1**	0.0	3
OE1119F	dTDPglucose 4,6-dehydratase	**4.7**		2	**1.2**	0.0	2			0
OE3538R	Hypothetical protein	**3.5**	2.0	4	**0.9**	0.1	2	**0.5**	0.1	2
OE3049R	Hypothetical protein	**2.7**	1.8	3	**1.2**	0.4	3	**1.3**	0.2	2
OE4187R	Probable DNA-binding protein	**2.6**	1.1	4	**1.4**	0.2	2	**0.2**	0.1	3
OE2082F	Conserved hypothetical protein	**2.5**	0.2	2	**1.1**	0.2	2			0
OE2024F	Conserved hypothetical protein	**2.4**	0.6	3	**1.1**	0.4	2	**0.7**		1
OE1114F	Probable glucose-1-phosphate thymidylyltransferase GraD3	**2.4**	0.6	3	**1.3**	0.2	2	**1.5**	0.1	3
OE1982R	Conserved hypothetical protein	**2.2**	0.3	2	**1.0**	0.0	2	**1.5**		1
OE3090R	Conserved hypothetical protein	**2.2**	0.8	4	**1.1**	0.3	3			0
OE1222R	tRNA adenylyltransferase, CCA-adding	**2.2**	0.2	2	**1.1**	0.1	3	**2.3**	0.7	2
OE2021F	Conserved hypothetical protein	**2.1**	0.9	4	**1.0**	0.1	2	**0.8**		1
OE1385F	Conserved hypothetical protein	**2.1**	0.7	4	**1.0**	0.2	3	**0.9**	0.2	2
OE1147R	Protein-L-isoaspartate O-methyltransferase PimT1	**2.0**	1.2	4	**1.0**	0.1	2	**1.1**	0.2	3

OE3470F	Translation initiation factor aIF-1A	**0.6**	0.2	4	**8.4**	6.1	3	**0.7**	0.1	3
OE1405R	Conserved hypothetical protein	**2.4**	0.9	4	**5.1**	0.2	3	**0.3**	0.0	3
OE1083R	Probable transposase (ISH3/ISH27)	**1.5**	0.4	4	**3.1**	1.1	2	**1.0**	0.3	3
OE5071F	Protein kinase weak homolog	**0.6**	0.3	4	**2.7**	0.1	3			0
OE7017R	Probable transposase (ISH3/ISH27)	**0.6**	0.2	4	**2.7**	0.4	3	**1.1**	0.2	3
OE3100F	Bacterioopsin-linked protein Blp	**0.6**	0.1	3	**2.5**	0.3	3	**3.2**	0.6	3
OE2433A1F	Probable transposase (ISH3/ISH27)	**1.0**	0.5	4	**2.3**	0.5	3	**1.5**	0.4	3

^*1^All genes are tabulated which have a "translational repression factor" of ≥2 differentially in exponential phase (upper part) or in stationary phase (lower part). The translational repression factor is the quotient of free RNA to polysome-bound RNA (normalized to the average of all genes). If available, also the relative transcript levels of these genes are listed (quotient stationary phase/exponential phase). ORF numbers and gene products were taken from the genome website HaloLex [62]. The columns ∅, SD and n list average value, standard deviation and number of repetitions.

The biological reason for translational regulation of the other 20 genes is less clear. 10 of the genes are annotated to encode "hypothetical proteins". The association of their transcripts with polysomes in one growth phase strongly indicates that the proteins are produced during that phase and proves that the proteins are not "hypothetical". Three genes encode transposases for one family of insertion elements (ISH3/ISH27), indicating that transposition of members of this family is less efficient in stationary phase. The remaining seven genes belong to different functional categories.

### Confirmation of microarray-based results of 14 genes using qRT-PCR

To confirm the results with an independent method, quantitative Realtime Reverse Transcription PCR (qRT-PCR) was applied. Eight of the 21 differentially regulated genes were chosen, five with a translational repression in the exponential phase and three that were undertranslated during stationary phase. As a control group, six genes were chosen arbitrarily which had average translational efficiencies in both growth phases. Three biological replicates were performed. All qRT-PCR curves were analyzed using the $2^{-\Delta \Delta {\text{C}}_{\text{T}}}$ method [35] and normalized to the average of the control group. In Table 2 the qRT-PCR results of all 14 genes are summarized and compared with the DNA-microarray results. In 13 of the 14 cases, the results obtained with both methods were in agreement. In three cases, the values obtained with DNA microarrays were higher than those obtained with qRT-PCR (Table 2). In one case, qRT-PCR and DNA microarray analysis yielded deviating results (OE5071F). One theoretical explanation, i.e. cross-hybridization of other genes on the microarray, could be excluded because there are no similar sequences in the genome. The reason for the conflicting results with gene OE5071F could not be clarified.

**Table 2:** Verification of DNA microarray-derived results with qRT-PCR^*1^

	Translational repression factor exp						Translational repression factor stat					
	
ORF	Realtime PCR			Microarray			Realtime PCR			Microarray		
	
	∅	SD	n	∅	SD	n	∅	SD	n	∅	SD	n
OE2010R	**1.2**	0.1	3	**1.0**	0.0	4	**1.2**	0.4	3	**1.0**	0.1	3
OE2055F	**1.0**	0.1	3	**1.0**	0.2	4	**1.3**	0.1	3	**1.0**	0.1	3
OE2563R	**0.8**	0.1	3	**1.0**	0.1	4	**0.7**	0.1	3	**1.0**	0.1	3
OE2595F	**1.2**	0.1	3	**1.0**	0.2	4	**1.0**	0.1	3	**1.0**	0.1	3
OE3637R	**0.7**	0.1	3	**1.0**	0.2	4	**0.9**	0.4	3	**1.0**	0.1	3
OE4674F	**1.1**	0.1	2	**0.9**	0.1	4	**0.8**	0.2	3	**0.9**	0.0	3

OE4511R	**45.4**	16.3	3	**9.0**	3.8	4	**12.7**	5.3	3	**1.2**	0.2	3
OE3538R	**19.6**	2.9	3	**3.5**	2.0	4	**4.3**	1.4	3	**0.9**	0.1	2
OE4187R	**8.7**	1.0	3	**2.6**	1.1	4	**6.4**	0.7	3	**1.4**	0.2	2
OE1119F	**3.0**	1.0	3	**4.7**		2	**1.4**	0.2	3	**1.2**	0.0	2
OE3049R	**2.2**	0.6	3	**2.7**	1.8	3	**1.6**	0.2	3	**1.2**	0.4	3

OE1405R	**9.2**	1.2	3	**2.4**	0.9	4	**13.5**	4.9	3	**5.1**	0.2	3
OE3470F	**1.9**	1.0	3	**0.6**	0.2	4	**7.4**	4.1	3	**8.4**	6.1	3
OE5071F	**2.7**	0.6	3	**0.6**	0.3	4	**1.5**	0.3	3	**2.7**	0.1	3

^*1^The relative levels of free mRNA and polysome-bound mRNA of selected genes were quantified by qRT-PCR in exponential phase ("exp") and stationary phase ("stat") cultures. The results for the 14 genes were normalized to the average values of six genes (upper part, OE2010R – OE4674R) which were presumed to be not translationally regulated based on the DNA microarray results. For comparison, the DNA microarray results are also listed. ORF numbers were taken from the genome website HaloLex [62]. The columns ∅, SD and n list average value, standard deviation and number of repetitions.

The possibility of cross-hybridization was also an issue for the gene encoding the translation initiation factor discussed above (OE3470F), because the *H. salinarum *genome contains a second, orthologous gene (OE4136R), and both share a high sequence identity of 66%. However, the DNA microarray analysis as well as qRT-PCR both revealed that only one of the two transcripts is translationally regulated (OE3470F), while the other one (OE4136R) is translated with average efficiency in both growth phases (data not shown). In summary, the DNA microarray results could be confirmed with qRT-PCR in 13 out of 14 cases, and even very similar genes could be differentiated.

### *H. salinarum *genes with growth phase-dependent above-average translational efficiency

The microarray data were also analyzed with respect to genes with an above-average ribosome association. Genes were classified as growth phase dependent overtranslated if they showed an at least twofold below average level of free mRNA in one growth phase, but not in the other. This group is comprised of 394 different genes, equivalent to 20% of all genes analyzed (Table S1 – Additional file 1). 393 of these genes have a differential above average translational efficiency in exponential phase, while only a single gene encoding a hypothetical protein is better translated in stationary phase (OE3542R). The genomic organization of this group of genes is striking. 69% of these genes (271) are located on the megaplasmids of *H. salinarum*, while only 21% of all analyzed genes are megaplasmid encoded. In total, 68% of all megaplasmid-encoded genes are translated at least twofold better and 90% are translated at least 1.5 fold better than average. This is in contrast to the genes localized on the chromosome. Only 8% of chromosomal genes are differentially translated with above-average efficiency.

171 of the 394 differentially translated genes are annotated as "hypothetical proteins", "conserved hypothetical proteins", or "genes with no known function" (Table S1). The differential translation of their transcripts proves that they are real genes and suggests that the encoded proteins are especially needed in exponential phase (OE3542R in stationary phase). Among the remaining 223 genes some functional categories are obviously enriched. Examples are 1) 33 genes involved in translation, including 27 ribosomal protein genes, 2) 13 genes for elements of the basal transcription machinery, including nine genes for TBP and TFB paralogs, 3) 21 genes for the chemotaxis machinery, including CheA, CheB, histidine kinases and haloarchaeal transducers, 4) 15 genes of the two gas vesicle gene clusters, and 5) 22 genes for transposases (Table S1). This non-random distribution prompted us to analyze the differential translational efficiencies of all *H. salinarum *genes as well as of the chromosomally encoded and of the megaplasmid encoded subfractions (Table 3). Exponential phase overtranslation was found for all functional categories of genes localized on megaplasmids. In contrast, among the chromosomally localized genes differential overtranslation is confined to very few functional categories. All genes of the functional classes "basal transcription apparatus" (TC) and "translation" (TL) are on average at least twofold better translated in exponential than in stationary phase. A somewhat lower average exponential phase overtranslation was also detected for the functional classes "chaperones" (CHP), "cellular processes" (CP), "signal transduction" (SIG), and "transposases and insertion elements" (ISH). These genes encode fundamental cellular processes like transcription, translation, protein folding, cell cycle and cell division, all of which have a higher activity in exponentially growing than in stationary phase cells.

**Table 3:** Growth phase-dependent translational regulation in different functional categories of the *H. salinarum *genome^*1^

	Whole genome			Main chromosome			Megaplasmids		
	
Functional class	∅	SD	n	∅	SD	n	∅	SD	n
**AA**	**1.0**	0.5	72	**1.0**	0.4	68	**1.7**	0.7	4
**CE**	**0.8**	0.3	2	**0.8**	0.3	2			
**CHM**	**0.7**	0.3	10	**0.7**	0.3	10			
**CHP**	**1.6**	0.8	3	**1.6**	0.8	3			
**CHY**	**1.4**	0.9	430	**0.9**	0.5	295	**2.3**	0.7	135
**CIM**	**1.2**	0.7	40	**1.2**	0.7	40			
**COM**	**1.0**	0.5	57	**1.0**	0.5	57			
**CP**	**1.7**	0.8	26	**1.5**	0.8	20	**2.2**	0.3	6
**EM**	**1.3**	0.7	39	**1.2**	0.7	37	**2.3**	0.5	2
**HY**	**1.2**	0.8	349	**0.8**	0.3	255	**2.3**	0.7	94
**ISH**	**1.9**	0.8	71	**1.1**	0.6	22	**2.3**	0.6	49
**LIP**	**1.1**	0.6	33	**1.1**	0.4	32	**3.6**		1
**MIS**	**1.3**	0.8	207	**1.1**	0.7	166	**2.1**	0.8	41
**MOT**	**0.9**	0.2	6	**0.9**	0.2	6			
**NOF**	**1.1**	0.6	312	**0.9**	0.4	274	**2.4**	0.7	38
**NUM**	**1.2**	0.9	39	**1.0**	0.7	37	**3.9**	0.2	2
**REG**	**1.1**	0.6	11	**0.9**	0.3	8	**1.8**	0.8	3
**RMT**	**0.8**	0.2	6	**0.8**	0.2	6			
**RRR**	**1.3**	0.8	33	**1.0**	0.6	26	**2.2**	0.7	7
**SEC**	**1.2**	0.9	9	**1.2**	0.9	9			
**SIG**	**1.8**	1.0	53	**1.8**	1.1	49	**2.1**	0.4	4
**TC**	**2.0**	0.9	26	**1.6**	1.0	14	**2.4**	0.6	12
**TL**	**2.1**	1.8	76	**2.1**	1.9	75	**2.5**		1
**TP**	**1.3**	0.8	103	**1.0**	0.5	81	**2.3**	0.7	22

^*1^The average "exponential phase induction factors" for all genes of the different functional categories are listed for the whole genome, the main chromosome, and the megaplasmids. Functional categories and corresponding genes were taken from the genome website HaloLex [62]. The exponential phase induction factors were calculated by dividing the translational repression factors in stationary phase with the translational repression factors in exponential phase. In addition to the average values (∅), the standard deviations (SD) and the number of genes in the respective functional classes (n) are listed. If no value is given, the megaplasmids do not contain genes of the respective functional class. The functional classes are: AA-amino acid metabolism, CE-cell envelope, CHM-carbohydrate metabolism, CHP-chaperones, CHY-conserved hypothetical protein, CIM-central intermediary metabolism, COM-coenzyme metabolism, CP-cellular processes, EM-energy metabolism, HY-hypothetical protein, ISH-transposases and ISH-encoded proteins, LIP-lipid metabolism, MIS-miscellaneous, MOT-motility, NOF-no function assigned to experimentally identified protein, NUM-nucleotide metabolism, REG-gene regulation, RMT-RNA maturation, RRR-replication, repair, recombination, SEC-protein secretion, SIG-signal transduction, TC-transcription, TL-translation, TP-small molecule transport.

The high number of genes with coordinate differential regulation indicates that a common regulatory mechanism exists. It seems well possible that they bind a common positive or negative translational regulator and thus form a "RNA regulon", as has recently been proposed to exist in eukaryotes [36,37]. An alternative explanation might be that the transcripts of this group of genes interact differently with elements of the basal translation initiation apparatus compared with the rest of genes. This would be analogous to the situation in *Arabidopsis thaliana*, where the translation initiation factor eIF3 is involved in the translational regulation of genes with specific 5'-leader sequences [38].

### *H. salinarum *genes with constitutive non-average translational efficiency

No genes were found with at least twofold lower translational efficiency and no growth phase-dependent translational regulation. In contrast, 17 genes showed at least twofold higher translational efficiency both in exponential and in stationary phase (Table S2 – Additional file 2). Examples are *cctA *and *cctB *encoding the thermosome and genes for a superoxide dismutase, a heat shock protein, and three subunits of an AAA-type ATPase. There are two possible explanations for this pattern of translation. 1) The genes could have a constitutive above-average translational efficiency. This possibility is reinforced by the fact that some of them belong to the group of predicted highly expressed (PHX) genes suggested by Karlin et al. [39], which are characterized by an optimal codon usage. 2) The genes could be differentially regulated, but not in response to growth phase, but to an as yet unidentified stimulus.

### Growth phase dependent transcript level regulation in *H. salinarum*

To enable a comparison of translational control and transcript level regulation in *H. salinarum*, the transcriptomes of exponentially growing and stationary phase cultures have been analyzed. Again, the data were normalized to the average of all genes, easing the identification of genes with non-standard transcriptional patterns. 353 genes had an at least twofold different transcript level in exponential versus stationary phase, compared to the average transcript level. The results for these genes are listed in Table S3 (Additional file 3), which also includes gene names, functional categories, and the degree of translational control. The 353 genes correspond to 17% of the 2120 genes with good hybridization signals in at least two out of three biological replicates. This fraction of differentially regulated genes is slightly smaller than that in *E. coli*. 27% of all *E. coli *genes have at least twofold different transcript levels in exponential versus stationary phase [40].

The majority of regulated genes have elevated transcript levels in exponential phase (243 of 353, Table S3). There is no enrichment of plasmid genes in this group in contrast to the group of overtranslated genes discussed above. 76% of the genes with above-average transcript levels have a known function, a considerably higher value than the average value of 60%. This indicates that these genes are involved in biological functions that have been extensively studied in the past. The largest group belongs to the functional group of "translation" and is comprised of genes encoding 40 ribosomal proteins, three translation factors and one aminoacyl-tRNA-synthetase. Only five ribosomal proteins were not found to have a more than twofold higher transcript level in exponential phase. Also the basal transcription machinery is differentially regulated (eight subunits of the RNA polymerase). This group also contains many genes encoding enzymes of the central metabolism, e.g. glucose degradation (6 genes), TCA cycle (5 genes), NADH dehydrogenase and electron transport (12 genes), and ATP-synthase (7 genes).

The genes with differentially induced transcript levels are enriched in genes that are also regulated on the translational level and thus show potentiation of the regulatory effects. 30% of the 243 genes with induced transcript levels in exponential phase are also overtranslated in exponential phase (average value 21%). The fraction of genes regulated on both levels is even more pronounced for highly regulated genes. Of the 61 genes with an at least fourfold transcript level induction in exponential phase, 39 are also translated with enhanced efficiency in exponential phase (Table S3). In some cases, interesting differences in regulatory strategies are obvious, e.g. the subunits of the RNA polymerase are regulated on the transcript level (and do not have differential translational control), while – as mentioned above – nine TBP and TFB paralogs were found to be translationally regulated (and are not regulated on the transcript level).

A smaller fraction of the genes have a differentially induced transcript level in stationary phase cells (110 of 353, Table S3). In contrast to the group of genes discussed above, this group of genes is not enriched in translationally induced genes. Among the genes with highest induction level are the subunits of the DMSO reductase, which is an alternative terminal oxidase under anaerobic conditions, and three subunits of an ABC transporter (sugar/glycerolphosphate-specific). Notably, this group of stationary phase-induced genes also includes several DNA repair proteins, two paralogs of the origin-binding Cdc6 protein, and two members of the "structural maintenance of chromosomes" (SMC) protein family. Induction of these genes underscores the importance to guarantee the integrity of chromosomal DNA in stationary phase. The remaining genes encode either proteins without a known function, or are scattered over various functional classes.

### Translational regulation in *Haloferax volcanii*

As one important aim was to address the evolutionary conservation of translational regulation, the analysis of non-average translational efficiencies in exponential and stationary phase was also performed with *Haloferax volcanii*. The genome of *H. volcanii *has been sequenced by TIGR, but the annotation has not been completed. Therefore a shotgun DNA microarray with a onefold coverage of the genome was used [41]. It is comprised of 2880 PCR products of an average length of 1.5 kbp. They have been sequenced from both ends and the encoded proteins have been deduced from BLAST searches against public databases. The clones contain full or partial sequences of one or two (in rare cases up to three) genes. The hybridization signals are thought to be dominated from the longest gene sequence of each clone. If the sequences of two genes have similar lengths in a specific clone, both are included in the results.

### Genes with growth phase-dependent differential translational regulation

A more than twofold differential repression was determined for 26 cloned genomic fragments and the results are summarized in Table 4. This represents 1.4% of the 1866 clones that generated significant hybridization signals in at least two of the biological replicates. Three pairs of cloned sequences overlap, reducing the number of identified translationally repressed genes to 23. Nine transcripts are translationally repressed in exponential phase, including those of genes for transcriptional regulators and one paralog of the basal transcription initiation factor TFB, which could potentiate the regulatory effect. 14 transcripts were translationally repressed in stationary phase, including the *csg *transcript encoding the cell surface glycoprotein.

**Table 4:** *H. volcanii *genes with a differential translational repression in exponential phase or in stationary phase^*1^

			Translational repression factor					
			
Identifier	Putative function	length (bp)	exp			stat		
			
			∅	SD	n	∅	SD	n
451B11	Inosine monophosphate dehydrogenase	365	**49.7**	56.0	3	**8.8**	2.9	3
431C4	Phage integrase-site-specific recombinase Xer	644	**17.5**	19.8	2	**1.9**	1.2	2
	Conserved hypothetical protein	528						
432A4	NAD dependent epimerase-dehydratase	770	**12.8**	5.6	4	**5.4**	2.9	3
439E12	Tfb2 – transcription initiation factor IIB	590	**10.4**	9.0	3	**1.6**	0.3	2
435D7	Nitrate transporter NarK	606	**7.6**	3.8	4	**3.0**	1.1	3
431H5	Alpha amylase AglA	613	**5.4**	0.5	2	**2.4**	1.3	3
	Nitrate transporter NarK	455						
435A4			**4.7**	2.7	3	**1.4**	0.8	2
437B8	HoxA-like transcriptional regulator (Hrg)	578	**4.5**	0.1	2	**1.5**	0.3	3
	Transcription regulator	413						
453C11	Tfb2 – transcription initiation factor IIB	335	**3.6**	0.3	2	**1.9**	0.9	3
451G12	Hypothetical protein	581	**2.5**	0.3	2	**0.9**	0.9	2
	Conserved hypothetical protein	441						
433F2	Glycosyltransferase	953	**2.3**	1.4	3	**0.5**	0.1	3
	Stage V sporulation protein R-like (SpoVR)	593						

435F2	CRISPR-associated protein, TM1814 family (Cas6)	222	**8.6**	3.5	4	**21.9**	12.7	3
432H1	Methyltransferase type 11	415	**5.0**	4.2	2	**13.1**	2.7	3
	Hypothetical protein	362						
	Domain of unknown function (DUF309) family	232						
443B4	Transposase (Tnp)	518	**4.5**	0.8	4	**7.9**	3.5	3
441F8	Tat pathway signal sequence domain protein	1008	**2.2**	0.1	3	**4.8**	2.6	3
459C3	Cell surface glycoprotein (Csg)	1167	**1.1**	0.3	4	**3.9**	2.0	3
445E9			**0.6**	0.4	2	**3.3**	0.2	2
433A10	Helicase family protein	1307	**1.1**	0.2	2	**3.3**	2.1	3
	Hypothetical protein	431						
441E1	DSBA-like thioredoxin domain protein	683	**1.3**	0.1	2	**2.8**	1.7	3
	Ferredoxin like protein	377						
	Mut-nudix family protein	363						
443C1	Ubiquinol oxidase subunit I, cyanide insensitive (CydA)	1391	**0.9**	0.1	3	**2.4**	1.1	3
431D10	Solute-binding periplasmic ABC transporter	1148	**1.0**	0.1	3	**2.3**	0.4	2
	ABC transporter, permease protein	475						
452E1	Ferrichrome-binding protein	848	**1.2**	0.3	4	**2.3**	0.6	3
451E8	Ribonuclease HII (RnhB)	579	**1.1**	0.5	4	**2.2**	0.7	3
	Preprotein-export translocase chain SecD	408						
460F5	Preprotein-export translocase chain SecD	988	**1.2**	0.6	3	**2.2**	0.2	3
440D1	Conserved hypothetical protein	992	**1.0**	0.1	3	**2.0**	0.0	2
444G9	Conserved hypothetical protein	1120	**0.5**	0.1	3	**2.0**	0.4	3
	Fibronectin type III domain protein	408						

^*1^All genes are tabulated which have a "translational repression factor" of ≥2 differentially in exponential phase (upper part) or in stationary phase (lower part). The translational repression factor is the quotient of free RNA to polysome-bound RNA (normalized to the average of all genes). The identifier is the clone designation in the onefold coverage genome library used to generate the shotgun DNA microarray [41]. The "putative functions" are derived from blast searches against public databases. The "length" is the partial or total length of the gene which is present in the respective clone. The columns ∅, SD and n list average value, standard deviation and number of biological replicates included in the analysis.

4.2% of *H. volcanii *genes were translated with at least twofold elevated translational efficiency in one of the two growth phases (79 clones, Table S4 – Additional file 4). In contrast to *H. salinarum*, the *H. volcanii *genes of this group are evenly distributed on the chromosome and the megaplasmids (21% of the regulated genes and 25% of all predicted ORFs are megaplasmid encoded) and no functional category is over-represented. If overlapping clones are taken into account, the 79 clones represent 71 different genes. Of these, 65 are overtranslated in exponential phase and 6 in stationary phase. Exponential phase overtranslated genes include genes for a paralog of a basal transcription initiation factor, several transcription regulators, and one translation initiation factor, which could potentiate the regulatory effect.

### *H. volcanii *genes with constitutive non-average translational efficiency

87 clones contain genes with an at least twofold elevated translational efficiency in both growth phases (Table S5 – Additional file 5). If overlapping clones are taken into account, the clones represent 58 different genes. Several of these genes belong to the "predicted highly expressed" genes [39], and examples of the encoded products are ribosomal proteins, RNA polymerase subunits, stress proteins, and a Cdc48 homologue. The biological function of the high translational efficiencies of the remaining genes, which represent different functional categories, is less clear.

28 clones contain genes with a constitutive more than twofold below-average translational efficiency, a category that is totally absent in *H. salinarum*. Virtually all of these genes encode hypothetical proteins or transposases. It might well be that the translational efficiency of these genes is not regulated in response to growth phase, but that another stimulus is needed for the induction of translation, e.g. specific stress conditions.

### Evolutionary conservation of translational regulation

Characterization of translational control in *H. salinarum *and *H. volcanii *has revealed that the fraction of regulated genes in both haloarchaeal species is much higher than anticipated for prokaryotes until now. In total, more than 400 genes and more than 100 genes are translated with differential efficiency in exponential phase compared to stationary phase cells in *H. salinarum *and *H. volcanii*, respectively. Therefore the fact that translational control constitutes a non-negligible part of the regulation of gene expression is shared by both species.

**Figure 2:**
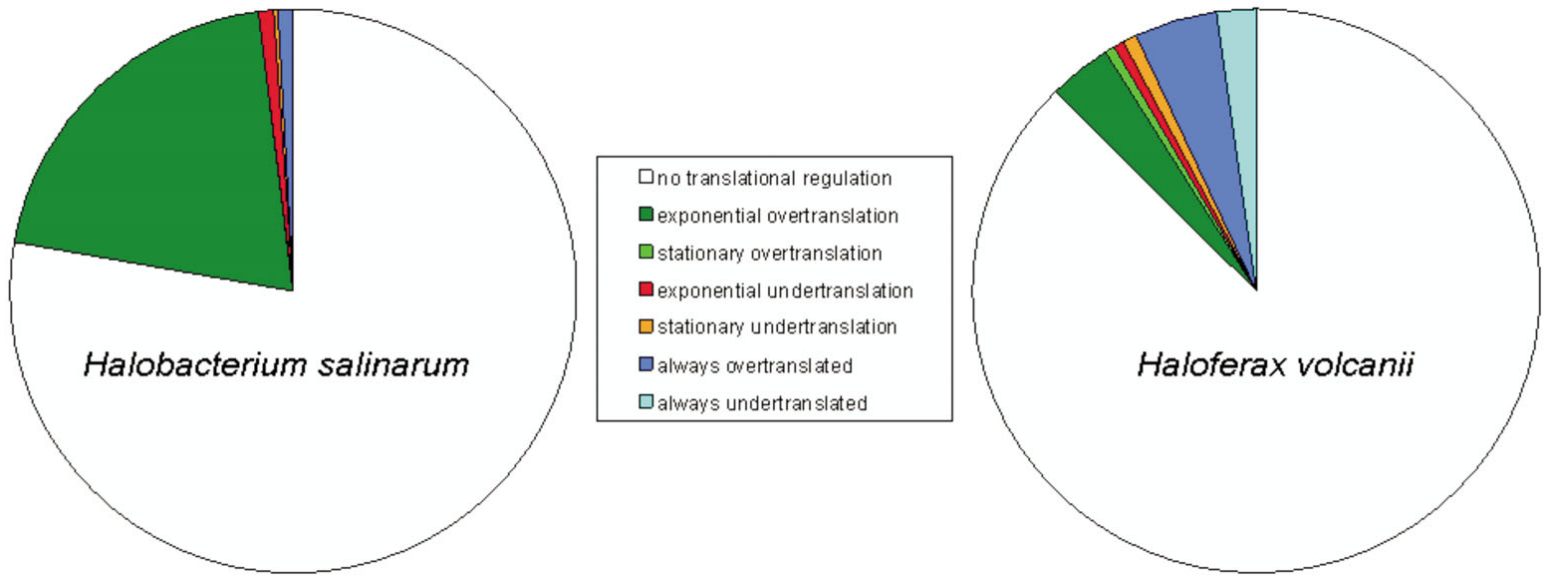
**Comparison of groups of translationally regulated genes in *Halobacterium salinarum *and *Haloferax volcanii***. The relative fractions of regulated genes, compared to the total number of expressed genes, were calculated for six groups of genes with different patterns of translational efficiencies in exponential phase and stationary phase, as indicated in the inset. The six groups with non-average translational efficiencies and the group of averagely-translated genes are graphically represented for *H. salinarum *and *H. volcanii*.

However, all other aspects are remarkably dissimilar: 1) the different groups of co-regulated genes have very different sizes. The fractions of all translationally-regulated groups of genes are compared in Figure 2, which illustrates the differences in both species, 2) the above-average translational efficiency of plasmid-encoded genes is only found in *H. salinarum*, 3) the group of genes with under-average translational efficiency in both growth phases is only found in *H. volcanii*, 4) most importantly, there is no overlap of genes that are regulated identically in both species.

These differences cannot easily be rationalized. Two very different types of DNA microarrays were used, but this could not have been the reason. The shotgun DNA microarray of *H. volcanii *was used in several studies and has generated very specific and biologically meaningful results, e.g. comparison of glucose-dependent versus amino acid-dependent metabolism [41], identification of genes involved in xylose catabolism (unpublished data), characterization of a regulon (Dambeck M, Soppa J: Characterization of the biological role of IftA (important for transition), a *Haloferax volcanii *member of the enolase superfamily, in preparation), and identification of a tryptophane-regulated promoter [42]. Characterization of translational regulation in eukaryotes revealed that – depending on the experimental conditions – very different sets of genes can be regulated even in the same species, e.g. in *S. cerevisiae *during amino acid starvation and after fusel alcohol addition (see below) [16]. However, in the present study the conditions were kept as identical as possible. Both species grew under aerobic conditions in complex medium with nearly identical growth rates with doubling times of about four hours. In both species mid-exponential cultures (4 × 10^8 ^cells/ml) were compared to cultures that had been cultivated in stationary phase (3 × 10^9 ^cells/ml) for 24 hours. Therefore, the only explanation seems to be that there is no evolutionary conservation of the identity of translationally regulated genes in these two haloarchaeal species of different genera. The different sets of growth phase-regulated genes add to the previously known differences of *H. salinarum *and *H. volcanii*. In short, *H. salinarum *is a rod-shaped flagellated archaeon with an optimal salt concentration of over 4 M NaCl. It can grow by aerobic respiration, arginine fermentation and phototrophically using bacteriorhodopsin. It can orient its swimming motility by chemotaxis, phototaxis and aerotaxis and, in addition, it can produce gas vesicles. In contrast, *H. volcanii *is a pleiomorphic archaeon with an optimal salt concentration of about 2.2 M NaCl. It can grow by aerobic and by nitrate respiration. It does not contain retinal proteins and does not produce flagella or gas vesicles.

A comparison whether translational control is conserved in other prokaryotic species is not possible, because no data are available for any other archaeal or for any bacterial species. However, several studies have been performed with three different species of eukaryotes, i.e. *S. cerevisiae, Arabidopsis thaliana*, and human cell lines [2,11-23]. The reported extent of translational regulation varies widely, from about 1% to about 25% of all expressed genes. Low fractions of translationally regulated genes were reported e.g. for *S. cerevisae *transferred from a fermentable to a nonfermentable carbon source [14] or *A. thaliana *starved for sucrose [20]. High fractions of translationally regulated genes were reported e.g. for *S. cerevisiae *treated with rapamycin [12] or *A. thaliana *during dehydration stress [19]. Quantitative comparisons between the different studies are not easy, because species, stimuli, experimental methods and data handling vary. However, it is clear that the applied stimulus can have tremendous influence on differential translational control. One example is the comparison of the effects of amino acid starvation and the addition of fusel alcohol to *S. cerevisiae *[16]. Although both treatments are thought to target translation initiation factor eIF-2B, 615 genes changed translational efficiencies after the former treatment, while only 167 genes were effected after the latter. Moreover, the two groups of genes were nearly mutually exclusive.

Even if comparisons of the results obtained with eukaryotes is hampered by the small number of species and the variability of conditions and experimental approaches, the available data do not indicate evolutionary conservation of translationally regulated genes among eukaryotes. This is in congruence with the lack of conservation of growth phase-regulated genes in two haloarchaeal species revealed in this study. Taken together, the results indicate that either translational regulation was added to the inventory for regulating gene expression late in evolution, or that the mechanism evolved early, but that the identity of translationally regulated genes and the level at which each gene is regulated are readily exchanged.

### Extent of transcriptional control, translational control, and potentiation

In total 17% of all expressed genes of *H. salinarum *have different transcript levels in exponential phase compared to stationary phase. The fraction of translationally regulated genes is even higher with a value of nearly 22%. If the large group of plasmid genes is subtracted as possibly exceptional, a value of 8% remains. But even this value is much higher than anticipated and it indicates that transcriptional and translational control are of similar importance for *H. salinarum*.

A comparison with bacteria is not possible, but several studies with eukaryotes have been performed. Similar fractions of transcriptionally and translationally regulated genes were found during stress response of yeast [16] and sucrose starvation of *Arabidopsis thaliana *[20]. Two further studies detected even more translationally than transcriptionally regulated genes, i.e. in oxygen-deprived *A. thaliana *seedlings [17] and in irradiated human cell lines [22]. Taken together, comparison of our new results with previous studies challenges the current view that the relative importance of transcriptional and translational regulation is very different in eukaryotes and prokaryotes.

The fraction of genes that is simultaneously regulated on the transcriptional and the translational level, which was called "potentiation" of regulation, seems to be specific for the species as well as for the stimulus used. A very good correlation with up to about 50% co-ordinately regulated genes occurred after heat shock, rapamycin treatment, and amino acid starvation of *S. cerevisiae *[12,14]. In contrast, a low level or no potentiation was detected after butanol-treatment of *S. cerevisiae*, rapamycin treatment and irradiation of human cells [16,21,22]. The results of *H. salinarum *are between these extremes. Coordinate changes of transcription and translation were detected for about 70 genes characterized by above-average transcript levels and translational efficiencies during exponential phase.

### Possible mechanisms of translational control

The different groups of translationally regulated genes are of very different size, e.g. 393 genes are differentially overtranslated and five are differentially undertranslated in exponential phase in *H. salinarum*. This indicates that several mechanisms – more general and more gene-specific – might operate in haloarchaea. In eukaryotes both types of mechanisms have been characterized. Many general initiation factors are involved in translation initiation, and their concentration or posttranslational modification can influence translational efficiencies of many genes [43,44]. In contrast, translational efficiencies of small groups of genes can be regulated by gene-specific regulatory proteins, which have specific binding sites in the 3'-UTRs or the 5'-UTRs of the respective transcripts [45-47]. In bacteria evidence is accumulating that small non-coding RNAs are involved in translational regulation [28,48].

Archaea share some of the translation initiation factors with eukaryotes [33] and harbor small non-coding RNAs [49], but molecular mechanisms of translational regulation have not been investigated. It has been proposed that archaea have two different classes of transcripts, i.e. transcripts with 5'-UTRs and leaderless transcripts, and that two different mechanisms of translation initiation exist [50]. Determination of the 5'-ends and 3'-ends of 40 haloarchaeal transcripts revealed that most of them are leaderless and that leadered transcripts with and without a Shine-Dalgarno sequence exist [51]. Several of the translationally regulated transcripts were found to lack a 5'-UTR, indicating that the 3'-UTR might be involved in regulation. A reporter gene system was established that permits to study the *in vivo *function of 5'-UTRs and 3'-UTRs [51], an *in vitro *translation system was established [52], and the generation of *in frame *deletion mutants is easily possible [53,54]. These methods will be applied to unravel the molecular mechanisms of translational regulation of the different groups of co-regulated genes described above, and to reveal whether principles known from eukaryotes or bacteria operate, or whether archaea-specific mechanisms have evolved.

## Conclusion

Global analysis of growth phase-dependent translational regulation in two haloarchaeal species revealed that the fraction of differentially regulated genes is much higher than anticipated for prokaryotes and is as high as had been detected in eukaryotes. There is only a small overlap between the translationally regulated genes of the two species, indicating a lack of phylogenetic conservation, again in congruence with the results obtained with three eukaryotic species. The large number of co-regulated genes indicates that "translational regulons" of transcripts exist which bind a common regulatory protein. The fraction of genes with growth phase-dependent differential regulation of the transcript level is in the same range as the translationally regulated genes. About 70 genes are regulated on both levels (potentiation of regulation), but the most genes are regulated by either of the two mechanisms.

## Methods

### Strains, media and culture conditions

*H. salinarum *(German culture collection strains DSMZ670 and DSMZ671) was grown in complex medium as described previously [55]. Growth phase-dependent translational and transcriptional regulation was studied with DSMZ670. DSMZ671 is a gas vesicle-negative derivative of DSMZ670. It had been used for genome sequencing (Pfeiffer F, Schuster SC, Broicher A, Falb M, Palm P, Rodewald K, Ruepp A, Soppa J, Tittor J, Oesterhelt D: Evolution in the laboratory: the genome of *Halobacterium salinarum *strain R1 as compared to strain NRC-1, Genomics, in press) and was therefore used for construction of the DNA microarray. *H. volcanii *WR340 [56] was kindly provided by Moshe Mevarech (Tel Aviv University, Israel) and was grown in complex medium according to Cline et al. [57]. Both species were cultivated aerobically at 42°C and 250 rpm, resulting in doubling times of 4 h.

### Isolation of free and polysome-bound RNAs

"Exponential phase" cultures were grown to a cell density of 5 × 10^8 ^cells/ml, and "stationary phase" cultures were incubated further 24 hours after onset of stationary phase. 200 ml of *H. salinarum *culture or 400 ml of *H. volcanii *culture were collected by centrifugation (15 min, 8000 *g*) and resuspended in buffer A (100 mM magnesium acetate, 10 mM HEPES pH 7.6, 3.4 M KCl for *H. salinarum *and 2.5 M KCl for *H. volcanii*). Cells were disrupted by sonication (3 × 30 s, output control 3, duty cycle 50%, Branson Sonifier 250), and the DNA was degraded by addition of 20 μl RNase-free DNase (RQ1 DNase, Promega, Mannheim, Germany) for 15 min at room temperature. 0.5 ml aliquots were laid on top of sucrose gradients consisting of 2 ml 50% (wt/vol) nuclease-free D-sucrose (Roth, Karlsruhe, Germany) at the bottom and a 9.5 ml gradient of 15% (w/v) to 40% (w/v) in buffer A. Centrifugation was for 16 h at 82000 *g *using a SW 40 Ti rotor (Beckman Coulter, Fullerton, USA). The gradients were divided into eight fractions of 1.5 ml. Initial small scale RNA isolation and analytical agarose gel electrophoreses were used to identify the positions of free RNA, small and large ribosomal subunits, and polysomes. The two fractions from the top were used to isolate free RNA, and the three bottom fractions to isolate polysome-bound RNAs.

### RNA isolation

For RNA isolation from sucrose gradients, corresponding fractions from four tubes were combinded and filled up to 9 ml with RNase-free water. RNA isolation was performed essentially as described [58,59]. Phenol chloroform extraction and ethanol precipitation were performed according to Gauthier et al. [59] except for different centrifugation conditions (15 min 3000 *g *and 30 min 48000 *g*). The RNA was suspended in 150 μl RNase free water. It was further purified with the RNeasy Midi kit (Qiagen, Hilden, Germany) following the manufacturer's instructions for RNA clean-up including DNase on column treatment. RNA concentration was determined photometrically, and its integrity was checked using denaturing formaldehyde gels [60].

For transcriptome analysis, RNA was isolated using the Qiagen RNeasy system (Qiagen, Hilden, Germany) with DNase on column treatment according to the manufacturer's instructions. For exponential phase cells, the Mini kit was used to isolate RNA from 1 × 10^9 ^cells, and for stationary phase cells, the Midi kit was used to isolate RNA from 1 × 10^10 ^cells.

### Isolation of genomic DNA from *H. salinarum*

Genomic DNA of *H. salinarum *was needed as template for PCR reactions to produce the DNA microarray. It was isolated essentially as described [61]. 10 ml of an exponentially growing culture was centrifuged (15 min, 4000 *g*) and the cells were resuspended in 300 μl basal salts (medium without carbon source). They were lysed by the addition of 2.5 ml lysis buffer (100 mM NaCl, 10 mM Tris pH 8.0, 1 mM EDTA, 0.05% (wt/vol) SDS). The lysate was overlaid with 3 ml ethanol and the precipitated DNA at the interphase was spooled onto a bended Pasteur pipette. The DNA was washed twice with ethanol and once with ether, dried and dissolved in TE (10 mM Tris pH 7.2, 1 mM EDTA).

### Production of DNA microarrays

The genome sequence of *H. volcanii *ist still not published, therefore production of a gene-specific DNA microarray was not possible. Production of a shotgun onefold-coverage DNA microarray for *H. volcanii *has been described previously [41]. It is comprised of 2880 cloned genome fragments of an average size of about 1.5 kbp. In the course of this project all clones were sequenced from both ends using standard methods, and the encoded proteins were identified by BLAST searches against public databases.

Some time ago the genome sequence of *H. salinarum *was completed [62] (Pfeiffer F, Schuster SC, Broicher A, Falb M, Palm P, Rodewald K, Ruepp A, Soppa J, Tittor J, Oesterhelt D: Evolution in the laboratory: the genome of *Halobacterium salinarum *strain R1 as compared to strain NRC-1, Genomics, in press) and enabled the generation of a gene-specific DNA microarray. For each gene, primers for PCR amplifications were designed with the software PrimeArray [63], using the following criteria: all primers had annealing temperatures of 70°C – 72°C, the PCR products were 300 bp to 500 bp in length, and sequence similarities to other parts of the genome apart of the target gene were minimized. The primers were obtained from Metabion (Munich, Germany). The PCR amplifications were performed in 96 well plates with 100 μl volume using HotStar*Taq*, buffer, and "Q solution" according to the manufacturer's instructions (Qiagen, Hilden, Germany). 200 ng genomic DNA of *H. salinarum *were included as template, the concentration for each primer was 0.5 μM, the dNTP concentrations were adjusted to the GC content of the genome (160 μM for dATP and dTTP and 240 μM for dCTP and dGTP). The PCR was conducted with 15 min initial denaturation at 95°C followed by 25 cycles of 1 min at 96°C, 30 sec at 55°C and 50 sec at 72°C followed by a final extension of 7 min at 72°C. The PCR products were checked by analytical agarose gel electrophoresis. The efficiency of PCR product generation of the 2530 genes was >95%. The PCR products were transferred to V-shaped 96 well plates and precipitated as described [41]. The DNA microarray was produced with a MicrogridII spotter (Genomic Solutions, Ann Arbor, MI, USA) as described for *H. volcanii *[41].

### DNA microarray analysis

RNA was isolated as described above and reverse transcribed into Cy3- or Cy5-labelled cDNA using random hexamer primers and M-MLV reverse transcriptase RNase H minus (Promega, Mannheim, Germany). cDNA generation and its preparation for hybridization were performed as described before [41] For the analyses of translational regulation, equal amounts of "free" and "ribosome-bound" RNA (10–20 μg each) were labelled. Dye-swap experiments were included to exclude effects of disproportionate incorporation of Cy3- or Cy5-dUTP.

For the analysis of transcript levels, initial experiments showed that the use of equal amounts of "exponential phase" RNA and "stationary phase" RNA samples led to a general under-representation of the stationary phase signals. Obviously the mRNA content of the total RNA used for cDNA generation is lower in stationary phase RNA. Therefore, 45 μg stationary phase RNA and 15 μg exponential phase RNA were used for reverse transcription and labelling.

Prehybridization of the microarrays, hybridization of the combined Cy3- and Cy5-labelled cDNA, and posthybridization processing was performed as described previously [41]. The hybridization temperatures were 68°C for *H. salinarum *and 64°C for *H. volcanii*, respectively.

### Data acquisition, data normalization and data analysis

Cy3- and Cy5-fluorescence intensities were acquired using a GenePix 4000A laser scanner (Axon Inc., Union City, USA) basically as described before [41]. The raw fluorescence data were processed with the software GenePixPro 3.0 (Axon Inc.). The further data analyses were performed using the spreadsheet software MS Excel (Microsoft, Redmond, USA). First, all spots were removed that did not meet the following criteria: 1) signal to noise ratio of at least 3 for the red or green fluorescence, 2) a fluorescence intensity of at least 100 (translational control) or 500 (transcriptional control) for at least one colour, and 3) flagged "good" by the spot finding software. If only fluorescence for the free RNA fraction could be detected, the quotient free to ribosomal was set to ∞ and this result was taken as a strong hint at a translational repression. As no internal standardization is possible in an experiment using density gradient fractions, these data were then normalized to the average of all evaluable data points. Then average values were calculated from the biological replicates. Only genes with results from at least two replicates were further analyzed. Genes were considered undertranslated or overtranslated if they deviated from the average by ≥2 or ≤0.5 in at least one growth phase (on average and in at least two single measurements).

### Database submission

All DNA microarray results have been submitted to the ArrayExpress database and obtained the following accession numbers: determination of differential translational efficiencies and transcript levels of *H. salinarum *– E-MEXP-1180; determination of differential translational efficiencies of *H. volcanii *– E-MEXP-1191.

### Quantitative Realtime RT PCR

RNA was isolated as described above. Reverse transcription of 2 μg RNA was carried out with 400 U M-MLV reverse transcriptase RNase H minus (Promega, Mannheim, Germany) and 0.6 μg random hexamer primers (Sigma, Steinheim, Germany) in 40 μl 1× reaction buffer (Promega) in the presence of 0.2 mM dATP and dTTP as well as 0.3 mM dCTP and dGTP. Prior to enzyme addition, the reaction mix was heated for 10 min to 65°C and cooled on ice for 2 min. After reverse transcriptase addition, the reaction was performed for 1 h at 42°C. Then, additional 200 U reverse transcriptase were added and the incubation was continued for 1 h. Finally the reaction was heat inactivated for 5 min at 80°C.

The quantitative Realtime PCR was performed in a RotorGene 3000 (Corbett Research, Melbourne, Australia) in a volume of 25 μl with DyNAmo SYBR Green qPCR Mastermix (Finnzymes Oy, Espoo, Finland), 0.4 μM each forward and reverse primer (biomers.net, Ulm, Germany; sequences see Table S6 – Additional file 6) and cDNA in an appropriate dilution (usually 0.5 μl) as template. Controls without template and controls without RT reactions were included. Average data for the genes OE2010R, OE2055F, OE2563R, OE2595F, OE3637R and OE4674F, which had been found to be unregulated using the microarrays, were used for normalization of the results. The PCR consisted of 10 min initial denaturation at 94°C, at least 50 cycles of 30 sec 94°C, 45 sec 60°C (OE1405R, OE3470F, OE4136R: 68°C), 30 sec 72°C and a final extension for 5 min at 72°C. A subsequent melting point analysis was performed to check the uniformity of the product. Data analysis was conducted with the RotorGene 6.0 software (Corbett Research). Relative levels of "free" mRNA to "polysome-bound" mRNA were calculated according to the $2^{-\Delta \Delta {\text{C}}_{\text{T}}}$ method [35], first normalizing the C_T _of each RNA fraction to the average C_T _of the presumed unregulated genes (see above), and then setting the amount of "polysome-bound" RNA to 1. Three biological replicates were performed for each gene.

## Authors' contributions

AZ, CL and MH performed the global study of translational regulation. CL performed the global transcriptome analysis and the Quantitative Realtime RT PCR. AZ, JT, GR, SCS, DO, and JS cooperated in the development of *H. salinarum *microarrays. SCS sequenced the 2880 PCR products comprising the *H. volcanii *DNA microarray. AZ established the microarray techniques. JS designed research. CL and JS wrote the manuscript. All authors read and approved the final manuscript.

## Supplementary Material

Additional file 1 *H. salinarum *genes with growth phase-dependent above-average translational efficiencies Click here for file

Additional file 2 *H. salinarum *genes with constitutive non-average translational efficiency Click here for file

Additional file 3 Growth phase dependent transcriptional regulation in *Hb. salinarum* Click here for file

Additional file 4 *H. volcanii *genes with growth phase-dependent above-average translational efficiency Click here for file

Additional file 5 *H. volcanii *genes with constitutive non-average translational efficiency Click here for file

Additional file 6 Oligonucleotides used in the quantitative Realtime PCR analysis Click here for file
