# Supplementary material for: Genome-wide analysis of growth phase-dependent translational and transcriptional regulation in halophilic archaea
Source: BMC Genomics. 2007 Nov 12;8:415. doi: 10.1186/1471-2164-8-415 (PMC3225822; doi:10.1186/1471-2164-8-415)
Supplement: Additional file 1 — H. salinarum genes with growth phase-dependent above-average translational efficiencies [file 1471-2164-8-415-S1.pdf]

Table S1. *H. salinarum* genes with growth phase-dependent above-average translational efficiencies[*1]

ORF	gene product	translational repression factor						transcript level		
		exp			stat			stat/exp		
		∅	SD	n	∅	SD	n	∅	SD	n
**Stationary growth phase overtranslation**										
OE3542R	hypothetical protein	**0.5**	0.1	4	**0.2**	0.0	3	**2.6**	0.3	3
**Exponential growth phase overtranslation**										
<u>AA - amino acid metabolism</u>										
OE1270F	glutamate dehydrogenase	**0.3**	0.2	4	**0.6**	0.2	3	**0.4**	0.1	3
OE1916F	cysteine synthase CysK1	**0.4**	0.1	4	**0.9**	0.1	3	**0.5**	0.0	3
OE3331R	anthranilate synthase beta chain	**0.5**	0.2	4	**1.0**	0.2	2			0
OE3959R	branched-chain-amino-acid transaminase	**0.3**	0.1	4	**0.6**	0.1	2			0
OE5205R_a	ornithine carbamoyltransferase, catabolic	**0.3**	0.1	4	**0.6**	0.1	3	**0.9**	0.2	3
OE5205R_b	ornithine carbamoyltransferase, catabolic	**0.3**	0.1	4	**0.7**	0.1	2	**1.1**	0.2	3
<u>CHP - chaperones</u>										
OE1737R	DnaK-type molecular chaperone Hsp70	**0.4**	0.1	4	**0.9**	0.3	3	**0.5**	0.1	3
<u>CIM - central intermediary metabolism</u>										
OE1500R	pyruvate, water dikinase (PEP synthase)	**0.4**	0.1	4	**0.9**	0.0	3	**0.2**	0.0	3
OE3195F	succinate-CoA ligase (ADP-forming) beta chain	**0.3**	0.2	4	**1.0**	0.2	3	**0.3**	0.0	3
OE3196F	succinate--CoA ligase (ADP-forming) alpha chain	**0.3**	0.1	4	**0.9**	0.0	3	**0.6**	0.5	3
OE3308F	malate dehydrogenase (oxaloacetate decarboxylating)	**0.4**	0.1	4	**0.9**	0.2	3	**0.7**	0.0	3
OE3634F	isocitrate dehydrogenase (NADP+)	**0.4**	0.1	4	**0.9**	0.2	2	**0.4**	0.0	3
OE4613F	aconitate hydratase	**0.3**	0.1	4	**0.7**	0.2	3	**0.5**	0.1	3
<u>COM - coenzyme metabolism</u>										
OE2057F	thiamin biosynthesis protein ThiC	**0.4**	0.1	4	**1.1**	0.1	3	**0.3**	0.0	3
OE3207F	probable precorrin-8W decarboxylase	**0.4**	0.1	4	**0.8**	0.1	3	**0.1**	0.0	3
OE3209F	probable precorrin-2 C20-methyltransferase	**0.4**	0.2	4	**0.8**	0.1	3	**0.2**	0.1	3
OE3524F	pyridoxine biosynthesis protein	**0.4**	0.1	4	**0.7**	0.2	3	**0.5**	0.0	3
<u>CP - cellular processes</u>										
OE1076F	cell division control protein Cdc6 homolog	**0.3**	0.1	4	**1.1**	0.3	2			0
OE1275F	proteasome alpha chain	**0.4**	0.1	4	**0.7**	0.1	2	**0.5**	0.1	3
OE1512R	segregation and condensation protein ScpA	**0.3**	0.1	4	**0.8**	0.0	2	**1.1**	0.1	3
OE1515R_a	structural-maintenance-of-chromosomes protein	**0.3**	0.2	4	**0.9**	0.1	2	**1.2**	0.2	3
OE1515R_b	structural-maintenance-of-chromosomes protein	**0.4**	0.2	4	**1.1**	0.3	2	**1.8**	0.1	3
OE1515R_c	structural-maintenance-of-chromosomes protein	**0.4**	0.2	4	**0.9**	0.3	2	**1.7**	0.1	3
OE1515R_d	structural-maintenance-of-chromosomes protein	**0.4**	0.2	4	**1.0**	0.1	2	**3.7**	1.9	3
OE1515R_e	structural-maintenance-of-chromosomes protein	**0.4**	0.2	4	**1.2**	0.3	2	**2.9**	1.0	3
OE1559R	cell division protein FtsZ1	**0.3**	0.1	4	**1.3**	0.5	2			0
OE2296F	proteasome beta chain	**0.4**	0.2	3	**0.8**	0.1	2	**0.8**	0.1	3
OE3693F	peptidylprolyl isomerase	**0.4**	0.1	4	**1.0**	0.1	3	**0.5**	0.2	3
OE3718F	cell division protein	**0.4**	0.2	4	**0.9**	0.1	3	**1.1**	0.2	3
OE4184F	cell division control protein Cdc6 homolog	**0.3**	0.1	4	**1.0**	0.1	2			0
OE5019F	cell division control protein Cdc6 homolog	**0.4**	0.2	4	**1.0**	0.1	3	**1.1**	0.1	3
OE5069F	cell division control protein Cdc6 C-terminal region homolog	**0.5**	0.1	4	**1.0**	0.1	2			0
OE5142F	cell division protein FtsZ5	**0.4**	0.1	4	**0.9**	0.0	3			0
OE5381F	cell division control protein Cdc6 homolog (pseudogene, N-terminal part, interrupted by frameshift)	**0.3**	0.1	4	**1.0**	0.2	3			0
OE6037R	cell division control protein Cdc6 homolog	**0.4**	0.1	4	**1.3**	0.6	2			0

OE7115F	cell division control protein Cdc6 homolog	**0.4**	0.2	4	**1.0**	0.0	2	**1.1**	0.2	3
EM - energy metabolism										
OE1979R	cytochrome-c oxidase chain I	**0.4**	0.1	4	**1.0**	0.2	3	**1.2**	0.2	3
OE3984R	H+-transporting two-sector ATPase chain B.a	**0.3**	0.1	4	**1.0**	0.1	3			0
OE3985R	H+-transporting two-sector ATPase chain A.a	**0.5**	0.1	4	**1.1**	0.3	3	**0.2**	0.0	3
OE3987R	H+-transporting two-sector ATPase chain C.a	**0.5**	0.2	4	**0.9**	0.1	3	**0.2**	0.1	3
OE3989R	H+-transporting two-sector ATPase chain K.a	**0.5**	0.1	4	**1.1**	0.1	3	**0.1**	0.0	3
OE7066F	cytochrome d ubiquinol oxidase chain II	**0.5**	0.2	4	**0.9**	0.1	2	**0.9**	0.3	3
OE7084F	thioredoxin	**0.3**	0.1	4	**0.9**	0.0	2	**0.9**	0.1	3
ISH - transposases and ISH-encoded proteins										
OE1058R	probable transposase (ISH1)	**0.5**	0.3	3	**0.9**	0.1	2			0
OE5062R	IS1341-type transposase (TCE31)	**0.4**	0.1	4	**1.2**	0.3	2	**0.6**	0.0	3
OE5063R	probable IS200-type transposase (TCE31)	**0.4**	0.1	3	**1.1**	0.4	2	**0.7**	0.1	3
OE5102R	probable IS1341-type transposase (ISH12)	**0.4**	0.3	4	**1.0**	0.2	3			0
OE5104R	probable IS200-type transposase (ISH12)	**0.5**	0.3	4	**1.0**	0.1	2	**1.3**	0.3	3
OE5180F	probable transposase (TCE33)	**0.5**	0.2	4	**0.9**	0.0	2			0
OE5233F	probable transposase (ISH5)	**0.4**	0.2	4	**1.0**	0.0	3	**0.9**	0.1	3
OE5260F	probable transposase (ISH10)	**0.4**	0.1	4	**1.1**	0.0	2			0
OE5338R	transposase homolog (ISH8/ISH26)<br>(nonfunctional, C-terminal part, interrupted by ISH1)	**0.5**	0.2	4	**0.9**	0.1	3	**1.1**	0.3	3
OE5339R	probable transposase (ISH1)	**0.4**	0.1	4	**0.8**	0.2	2	**1.1**	0.2	3
OE5393F	insertion element protein (ISH2)	**0.4**	0.1	4	**1.2**	0.3	2			0
OE5407F	probable transposase (ISH8/ISH26)	**0.4**	0.1	4	**1.1**	0.1	2	**1.2**	0.3	3
OE5418F	insertion element protein (ISH2)	**0.4**	0.1	4	**1.1**	0.3	2	**0.7**	0.2	3
OE6012F	probable transposase (ISH6/ISHS1)	**0.4**	0.1	4	**1.2**	0.2	2			0
OE6075R	probable transposase (ISH8/ISH26)	**0.5**	0.2	4	**0.9**	0.2	3			0
OE6089R	IS1341-type transposase (TCE32)	**0.5**	0.2	4	**1.0**	0.2	3	**0.7**	0.1	3
OE6126R	insertion element protein (ISH2)	**0.4**	0.1	4	**1.0**	0.1	3	**0.9**	0.2	3
OE6133R	probable transposase (ISH10)	**0.5**	0.2	4	**0.9**	0.1	2	**1.1**	0.3	3
OE6165R	probable transposase (ISH3/ISH27)	**0.3**	0.1	4	**1.0**	0.1	3			0
OE6277R	transposase homolog (TCE33)<br>(nonfunctional, N-terminal part)	**0.3**	0.1	4	**0.9**	0.1	2	**0.7**	0.1	3
OE6296R	probable transposase (ISH8/ISH26)	**0.5**	0.3	3	**0.9**	0.2	2	**1.4**	0.3	3
OE6353F	insertion element protein (ISH2)	**0.4**	0.1	4	**1.0**	0.2	2	**0.9**	0.2	3
OE7006R	probable transposase (ISH7/ISH24)	**0.4**	0.2	4	**1.0**	0.0	2	**1.0**	0.2	3
OE7019F	probable transposase (ISH8/ISH26)	**0.4**	0.1	4	**1.2**	0.5	2	**0.7**	0.1	3
OE7052F	probable transposase (ISH5)	**0.3**	0.1	4	**1.0**	0.2	2	**1.0**	0.1	3
OE7159R	probable transposase (ISH7/ISH24)	**0.4**	0.1	4	**1.0**	0.1	2	**1.1**	0.3	3
OE7170R_a	probable transposase (ISH4/ISH23/ISH50)	**0.4**	0.1	4	**0.9**	0.2	2			0
OE7170R_b	probable transposase (ISH4/ISH23/ISH50)	**0.4**	0.2	4	**1.0**	0.3	2	**0.6**	0.1	3
LIP - lipid metabolism										
OE4500R	probable acyl/butyryl-CoA dehydrogenase	**0.4**	0.2	4	**0.9**	0.0	2	**0.5**	0.1	3
OE7093R	isopentenyl-diphosphate delta-isomerase	**0.3**	0.1	4	**1.2**	0.5	2	**0.8**	0.1	2
MIS - miscellaneous										
OE2378R	ParA domain protein (fla operon protein ParA1)	**0.4**	0.1	4	**1.0**	0.3	2	**0.5**		1
OE2386R	p-nitrophenylphosphatase homolog	**0.3**	0.1	4	**0.8**	0.1	2	**0.7**	0.2	2
OE2674R	rRNA (adenine-N6,N6-)-dimethyltransferase	**0.5**	0.1	4	**0.9**	0.2	2	**0.8**	0.2	3
OE2689F	fibrillarin	**0.4**	0.1	4	**0.9**	0.1	2			0
OE2853R	probable peptidylprolyl isomerase	**0.4**	0.2	4	**0.9**	0.1	3	**0.3**	0.0	3
OE3101R	bacterioopsin activator	**0.4**	0.1	4	**0.8**	0.1	3			0
OE3218F	cobalamin operon protein	**0.3**	0.1	4	**0.9**	0.2	3	**0.2**	0.1	3
OE3339R	AAA-type ATPase (transitional ATPase homolog)	**0.5**	0.2	4	**1.0**	0.2	2	**0.6**	0.1	2

OE3349F	coenzyme F420-quinone oxidoreductase 42K chain	**0.3**	0.1	4	**0.6**	0.1	2	**0.6**	0.1	3
OE3356F	AAA-type ATPase (transitional ATPase homolog)	**0.4**	0.1	4	**1.5**	0.4	2			0
OE3544F	probable oxidoreductase (ferredoxin reductase homolog)	**0.5**	0.1	4	**0.9**	0.1	3	**0.7**	0.1	3
OE3971R	ParA domain protein	**0.4**	0.1	4	**1.1**	0.2	2			0
OE4263F	conserved hem operon protein	**0.4**	0.1	4	**1.3**	0.4	2			0
OE4438F	leucyl aminopeptidase	**0.4**	0.2	4	**1.2**	0.2	3	**0.3**	0.1	3
OE4509F	nonhistone chromosomal protein	**0.4**	0.2	3	**0.8**	0.1	2	**0.9**	0.2	2
OE5094F	diaminobutyrate-pyruvate aminotransferase (siderophore biosynthesis protein)	**0.4**	0.2	4	**1.1**	0.1	2	**1.6**	0.3	3
OE5095F	L-2,4-diaminobutyrate decarboxylase (siderophore biosynthesis protein)	**0.5**	0.2	4	**0.9**	0.1	3	**1.4**	0.2	2
OE5096F	siderophore biosynthesis protein	**0.5**	0.1	4	**0.9**	0.1	2	**0.8**	0.2	3
OE5097F	siderophore biosynthesis protein (malonyl-CoA decarboxylase homolog)	**0.4**	0.2	4	**1.1**	0.3	2	**1.1**	0.1	3
OE5099F	siderophore biosynthesis protein	**0.5**	0.2	4	**0.9**	0.1	2			0
OE5112R	gas-vesicle operon protein GvpM2	**0.5**	0.2	4	**0.9**	0.1	3			0
OE5113R	gas-vesicle operon protein GvpL2	**0.5**	0.1	4	**1.0**	0.1	2	**0.9**	0.1	3
OE5114R	gas-vesicle operon protein GvpK2	**0.4**	0.2	4	**1.2**	0.0	3	**1.0**	0.2	3
OE5116R	gas-vesicle operon protein GvpJ2	**0.4**	0.2	4	**0.8**	0.1	2	**1.3**	0.4	3
OE5121R	gas-vesicle operon protein GvpG2	**0.4**	0.1	4	**0.9**	0.1	2	**0.7**	0.0	2
OE5123R	gas-vesicle operon protein GvpE2 (probable activator)	**0.5**	0.1	4	**0.9**	0.2	3	**1.0**	0.1	3
OE5125F	gas-vesicle protein GvpA2	**0.4**	0.2	4	**0.9**	0.1	3	**0.9**	0.1	3
OE5127F	hypothetical protein	**0.4**	0.1	4	**1.0**	0.2	2	**0.7**	0.2	3
OE5128F	gas-vesicle operon protein GvpN2	**0.4**	0.2	4	**1.2**	0.5	2	**1.8**	0.4	3
OE5129F	gas-vesicle operon protein GvpO2	**0.5**	0.2	4	**0.8**	0.1	2			0
OE5186R	catalase (including: peroxidase)	**0.2**	0.1	4	**0.7**	0.1	3	**0.6**	0.1	3
OE5189F	probable acyl-CoA transferase (3-oxoacid CoA-transferase homolog)	**0.5**	0.1	4	**1.6**	0.2	2	**0.8**	0.1	3
OE5192R	alkaline phosphatase	**0.3**	0.1	4	**1.0**	0.0	3	**2.5**	0.4	3
OE5337R	probable ATP-dependent helicase (nonfunctional, C-terminal part, interrupted by ISH8)	**0.5**	0.2	4	**1.5**	0.3	2	**0.9**	0.0	3
OE5436R	probable arsenate reductase	**0.3**	0.1	4	**1.1**	0.2	2			0
OE6311F	AAA-type ATPase (transitional ATPase homolog)	**0.4**	0.1	4	**1.0**	0.2	2			0
OE7010R	ParA domain protein (nonfunctional, N-terminal part, interrupted by ISH7)	**0.4**	0.1	4	**1.0**	0.0	2			0
OE7024R	gas-vesicle operon protein GvpK1	**0.4**	0.2	4	**0.9**	0.1	2	**0.8**	0.0	3
OE7026R	gas-vesicle operon protein GvpJ1	**0.5**	0.2	4	**1.3**	0.3	2			0
OE7028R	gas-vesicle operon protein GvpH1	**0.5**	0.2	4	**0.9**	0.0	2	**0.9**	0.2	3
OE7036F_a	gas-vesicle protein GvpC1	**0.5**	0.2	4	**0.9**	0.1	2			0
OE7036F_b	gas-vesicle protein GvpC1	**0.4**	0.1	4	**0.8**	0.1	2			0
OE7037F	gas-vesicle operon protein GvpN	**0.4**	0.1	4	**1.3**	0.3	2			0
OE7038F	gas-vesicle operon protein GvpO1	**0.5**	0.2	4	**1.0**	0.2	2	**0.6**	0.0	3
NUM - nucleotide metabolism										
OE2667F	nucleoside-diphosphate kinase	**0.4**	0.1	4	**1.8**	0.9	2	**0.5**	0.1	3
OE5201F	aspartate carbamoyltransferase catalytic chain	**0.3**	0.1	4	**1.1**	0.4	2	**0.2**	0.1	2
OE5202F	aspartate carbamoyltransferase regulatory chain	**0.2**	0.1	4	**1.0**	0.3	2	**0.2**	0.0	3
REG - gene regulation										
OE5209R	transcription regulator ArcR	**0.4**	0.2	4	**0.8**	0.2	3	**1.2**	0.1	3
OE5443F	probable transcription regulator	**0.4**	0.2	4	**1.0**	0.2	2	**0.7**	0.1	3
RRR - replication, repair, recombination										
OE4282F	single-stranded-DNA-specific exonuclease	**0.4**	0.1	4	**1.1**	0.3	2	**0.7**	0.0	3
OE4293R	DNA-directed DNA-polymerase type III chain 2 (intein-containing)	**0.4**	0.1	4	**0.9**	0.0	2			0
OE4466R	DNA repair protein	**0.3**	0.1	4	**0.6**	0.2	3	**0.4**	0.1	3

OE6036F_a	probable DNA-directed DNA polymerase type II	**0.4**	0.2	4	**1.1**	0.1	2			0
OE6036F_b	probable DNA-directed DNA polymerase type II	**0.5**	0.2	4	**1.1**	0.3	2	**1.0**	0.1	3
OE7114F	probable DNA-directed DNA polymerase type II	**0.5**	0.3	3	**0.9**	0.1	3			0
OE7194F	plasmid replication protein RepJ	**0.4**	0.1	4	**1.4**	0.2	2			0
SEC - protein secretion										
OE1347R	type II secretion system protein GspE1	**0.5**	0.2	4	**1.0**	0.2	2	**0.8**	0.1	3
OE4450R	signal recognition particle 54K protein	**0.3**	0.1	4	**1.0**	0.2	2	**0.7**	0.3	2
SIG - signal transduction										
OE1536R	transducer protein Htr14	**0.4**	0.1	4	**1.0**	0.1	2	**1.1**		1
OE2195F	transducer protein Htr18	**0.4**	0.1	4	**0.9**	0.2	3	**0.6**	0.2	3
OE2406R	protein-glutamate O-methyltransferase CheR	**0.4**	0.1	4	**1.3**	0.5	2			0
OE2415R	taxis sensor histidine kinase CheA	**0.2**	0.1	4	**0.6**	0.1	3	**0.5**	0.1	3
OE2416R	protein-glutamate methylesterase CheB	**0.3**	0.1	4	**0.7**	0.3	2	**0.5**	0.0	3
OE2474R	transducer protein HtrXIII	**0.4**	0.2	4	**0.9**	0.1	2	**0.6**	0.1	3
OE2996R	transducer protein Htr9 (HtpIII)	**0.5**	0.3	3	**0.9**	0.1	3			0
OE3070R	transducer protein HtrXII	**0.4**	0.2	4	**1.0**	0.2	3	**0.6**	0.1	3
OE3150R_a	transducer protein HemAT	**0.4**	0.1	4	**1.2**	0.1	2	**0.9**	0.0	3
OE3150R_b	transducer protein HemAT	**0.4**	0.1	4	**1.0**	0.1	2	**0.6**	0.1	3
OE3167F_a	transducer protein HtrVIII	**0.4**	0.2	4	**1.0**	0.1	3	**0.7**	0.1	3
OE3167F_b	transducer protein HtrVIII	**0.4**	0.2	4	**1.1**	0.3	2	**0.7**	0.1	3
OE3347F	Transducer protein HtrI	**0.3**	0.1	4	**1.0**	0.1	3			0
OE3436R_a	transducer protein Htr17	**0.5**	0.1	4	**1.2**	0.2	3	**0.8**	0.2	3
OE3436R_b	transducer protein Htr17	**0.4**	0.1	4	**1.0**	0.2	2			0
OE3473F	transducer protein HtrVII	**0.4**	0.0	4	**1.4**	0.8	2	**1.1**	0.2	3
OE3474R	transducer protein CosT	**0.5**	0.2	4	**1.1**	0.2	3	**0.7**	0.1	3
OE3481R_a	transducer protein HtrII	**0.3**	0.1	4	**2.0**	1.4	3			0
OE3481R_b	transducer protein HtrII	**0.4**	0.1	4	**0.9**	0.3	3	**0.7**	0.0	2
OE3611R_a	transducer protein BasT	**0.4**	0.2	4	**1.0**	0.2	2	**0.6**	0.1	2
OE3611R_b	transducer protein BasT	**0.5**	0.1	3	**1.0**	0.4	2	**0.7**	0.1	2
OE5243F_a	transducer protein Car	**0.3**	0.2	4	**0.7**	0.1	2	**0.4**	0.1	3
OE5243F_b	transducer protein Car	**0.4**	0.1	4	**1.0**	0.2	2	**0.8**	0.0	3
OE7042R	probable signal-transducing histidine kinase / response regulator	**0.5**	0.1	4	**0.9**	0.2	2	**0.9**	0.2	2
TC - transcription										
OE2631F	DNA-directed RNA polymerase chain D	**0.4**	0.2	4	**0.9**	0.0	3	**0.3**	0.1	3
OE2637F	DNA-directed RNA polymerase chain N	**0.3**	0.1	4	**0.8**	0.1	3			0
OE4146F	TATA-binding transcription initiation factor	**0.3**	0.1	4	**1.0**	0.3	2	**0.6**	0.1	3
OE4740R	DNA-directed RNA polymerase chain A'	**0.2**	0.0	3	**0.7**	0.1	3	**0.4**	0.1	3
OE4743R	DNA-directed RNA polymerase chain H	**0.4**	0.1	4	**0.7**	0.2	3	**0.3**	0.0	3
OE6026R	transcription initiation factor TFB	**0.4**	0.1	4	**1.4**	0.2	2			0
OE6071R	transcription initiation factor TFB	**0.4**	0.1	4	**1.2**	0.5	2			0
OE6127F	TATA-binding transcription initiation factor (nonfunctional, C-terminal part, interrupted by ISH2)	**0.4**	0.3	3	**0.9**	0.2	2	**0.9**	0.2	2
OE6140R	transcription initiation factor TFB	**0.4**	0.1	4	**1.0**	0.1	2	**1.2**	0.3	3
OE7059R_a	TATA-binding transcription initiation factor (nonfunctional, C-terminal part, interrupted by ISH2)	**0.3**	0.1	4	**0.9**	0.1	2	**0.8**	0.1	3
OE7059R_b	TATA-binding transcription initiation factor (nonfunctional, C-terminal part, interrupted by ISH2)	**0.4**	0.2	4	**1.1**	0.1	2	**0.9**	0.2	3
OE7062R	TATA-binding transcription initiation factor (nonfunctional, N-terminal part, interrupted by ISH2)	**0.4**	0.2	4	**0.9**	0.1	3	**0.7**	0.1	3
OE7146R	transcription initiation factor TFB (nonfunctional, C-terminal part, interrupted by ISH8)	**0.5**	0.1	4	**0.9**	0.1	2			0
OE7214R	TATA-binding transcription initiation factor	**0.4**	0.2	4	**1.0**	0.3	2	**0.7**	0.0	2

TL - translation										
OE1821R	ribosomal protein L36a.eR (HLA)	**0.3**	0.1	4	**0.7**	0.3	3			0
OE2165R	ribosomal protein S15	**0.4**	0.1	4	**1.2**	0.1	2	**0.2**	0.1	3
OE2600R	ribosomal protein L12	**0.3**	0.0	4	**0.9**	0.2	3			0
OE2601R	ribosomal protein L10	**0.4**	0.2	4	**1.0**	0.2	3	**0.2**	0.0	3
OE2602R	ribosomal protein L1	**0.4**	0.2	4	**1.0**	0.0	3	**0.2**	0.1	3
OE2629F	ribosomal protein S11	**0.2**	0.1	4	**1.0**	0.7	2	**0.2**	0.0	3
OE2632F	ribosomal protein L18.eR	**0.5**	0.2	4	**0.9**	0.2	3	**0.2**	0.0	3
OE2641F	ribosomal protein S2	**0.4**	0.1	4	**0.9**	0.1	3	**0.2**	0.0	3
OE2664F	ribosomal protein S28.eR	**0.4**	0.1	4	**1.1**	0.2	3	**0.5**	0.1	3
OE3388F	ribosomal protein L3	**0.4**	0.0	4	**1.0**	0.3	3			0
OE3392F	ribosomal protein L2	**0.3**	0.0	4	**0.9**	0.2	3	**0.2**	0.1	3
OE3393F	ribosomal protein S19	**0.2**	0.0	4	**1.0**	0.1	3	**0.2**	0.1	3
OE3394F	ribosomal protein L22	**0.2**	0.0	4	**1.2**	0.2	2	**0.2**	0.0	3
OE3395F	ribosomal protein S3	**0.4**	0.2	4	**0.8**	0.1	3	**0.1**	0.0	3
OE3396F	ribosomal protein L29	**0.3**	0.1	4	**1.1**	0.1	3	**0.3**	0.0	3
OE3402F	ribosomal protein L14	**0.3**	0.1	4	**0.9**	0.2	3	**0.2**	0.1	3
OE3404F	ribosomal protein L24	**0.4**	0.1	4	**1.1**	0.2	2			0
OE3405F	ribosomal protein S4.eR	**0.4**	0.1	4	**1.0**	0.4	2	**0.1**	0.0	2
OE3411F	ribosomal protein L6	**0.2**	0.0	4	**0.9**	0.2	2	**0.1**	0.0	3
OE3412F	ribosomal protein L32.eR	**0.2**	0.0	4	**1.0**	0.2	2	**0.2**	0.1	3
OE3413F	ribosomal protein L19.eR	**0.3**	0.1	4	**1.0**	0.0	2	**0.2**	0.1	3
OE3414F	ribosomal protein L18	**0.4**	0.2	4	**1.0**	0.1	3	**0.2**	0.0	3
OE3416F	ribosomal protein L30	**0.2**	0.1	4	**1.1**	0.1	2	**0.3**	0.0	3
OE3487R	translation initiation factor aIF-5A	**0.4**	0.1	4	**1.0**	0.2	3	**0.4**	0.0	3
OE4459R	ribosomal protein L31.eR	**0.4**	0.1	4	**0.9**	0.1	3			0
OE4460R	ribosomal protein L39.eR	**0.3**	0.1	3	**0.9**	0.2	3	**0.3**	0.1	3
OE4721R	translation elongation factor aEF-1 alpha chain	**0.3**	0.1	4	**0.5**	0.1	3	**0.2**	0.0	3
OE4736R	ribosomal protein S12	**0.4**	0.1	4	**0.9**	0.3	2	**0.3**	0.0	3
OE5203F	arginine-tRNA ligase	**0.3**	0.2	3	**0.7**	0.1	3	**0.8**	0.2	3
TP - small molecule transport										
OE3910R	probable ABC-type transport system periplasmic substrate-binding protein	**0.3**	0.1	4	**1.0**	0.0	2	**0.4**	0.2	3
OE4301R	ABC-type transport system ATP-binding protein	**0.4**	0.2	4	**0.9**	0.1	3	**0.2**	0.0	3
OE4302R	ABC-type oligopeptide transport system ATP-binding protein	**0.4**	0.2	4	**1.1**	0.2	2	**0.1**	0.0	3
OE5101R	probable cationic amino acid transport protein	**0.5**	0.2	4	**1.5**	0.1	2	**0.7**	0.0	3
OE5132F	probable phosphate transport protein	**0.4**	0.1	4	**1.1**	0.1	2			0
OE5146R	ABC-type transport system ATP-binding protein	**0.5**	0.1	4	**1.6**	0.4	2			0
OE5147R	probable ABC-type transport system periplasmic substrate-binding protein	**0.4**	0.1	4	**1.1**	0.1	2			0
OE5168F	probable ABC-type transport system permease protein (sugar / sn-glycerol-3-phosphate transport protein)	**0.5**	0.2	4	**0.9**	0.2	2	**4.6**	1.9	3
OE5204R	probable Na+/H+-exchanging protein	**0.5**	0.1	4	**1.0**	0.2	3	**0.5**	0.0	3
OE5223R	ABC-type transport system ATP-binding protein (nonfunctional, C-terminal part, interrupted by ISH3)	**0.4**	0.2	4	**0.9**	0.1	3	**1.0**	0.1	3
OE5245F	ABC-type transport system ATP-binding protein	**0.4**	0.2	4	**1.0**	0.2	2	**0.7**	0.1	3
OE5255R	probable transport protein	**0.4**	0.2	4	**1.0**	0.1	2	**0.9**	0.1	3
OE5264F	ABC-type transport system ATP-binding protein	**0.4**	0.1	4	**1.2**	0.3	2			0
OE5268R	ABC-type transport system ATP-binding protein	**0.3**	0.1	4	**1.1**	0.1	3	**0.8**	0.1	3
OE5270R	ABC-type transport system permease protein	**0.4**	0.2	4	**0.9**	0.0	2	**0.8**	0.2	3
OE5272R	ABC-type transport system permease protein	**0.4**	0.2	4	**1.0**	0.0	2	**1.0**	0.1	3
OE5273R	ABC-type transport system periplasmic substrate-	**0.4**	0.2	4	**1.0**	0.0	2	**0.6**	0.1	3

	binding protein									
OE5307F	ABC-type transport system ATP-binding protein	**0.4**	0.1	4	**1.1**	0.2	2	**0.6**	0.0	3

<u>CHY - conserved hypothetical protein, HY - hypothetical protein, NOF - no function assigned to experimentally identified protein</u>

OE1231R	hypothetical protein	**0.5**	0.2	4	**1.0**	0.2	2	**0.7**	0.1	3
OE1293F	hypothetical protein (overlaps real protein)	**0.4**	0.2	4	**0.9**	0.1	2			0
OE1564R	Na+/Ca2+-exchanging protein homolog	**0.4**	0.2	4	**1.3**	0.5	2	**0.8**	0.1	3
OE2027F	conserved hypothetical protein	**0.4**	0.1	4	**1.3**	0.2	2	**0.8**	0.1	3
OE2044F	conserved hypothetical protein	**0.4**	0.2	4	**1.2**	0.6	2	**0.7**	0.1	3
OE2120F	conserved hypothetical protein	**0.3**	0.1	4	**1.3**	0.3	2	**0.7**	0.2	3
OE2160R	hypothetical protein	**0.3**	0.1	4	**0.8**	0.2	3	**0.3**	0.1	3
OE3050F	conserved hypothetical protein	**0.4**	0.2	4	**1.1**	0.1	2	**0.8**	0.1	3
OE3075R	hypothetical protein	**0.5**	0.2	4	**1.0**	0.2	2	**0.8**	0.1	3
OE3367F	DnaJ N-terminal domain protein	**0.4**	0.1	4	**1.3**	0.1	2	**0.7**	0.1	2
OE3398F	conserved hypothetical protein	**0.2**	0.0	4	**1.0**	0.2	2	**0.1**	0.0	3
OE3617F	conserved hypothetical protein	**0.4**	0.2	4	**0.9**	0.3	2	**1.0**	0.3	2
OE3815R	conserved hypothetical protein	**0.5**	0.1	4	**1.0**	0.2	3			0
OE4027F	conserved hypothetical protein	**0.4**	0.2	4	**1.0**	0.2	3	**0.8**	0.1	3
OE4393R	transport protein homolog	**0.4**	0.1	4	**1.3**	0.1	2	**0.5**	0.1	3
OE4627F	hypothetical protein	**0.4**	0.1	4	**1.0**	0.3	2			0
OE4713R	conserved hypothetical protein	**0.5**	0.1	4	**1.0**	0.1	3	**1.2**	0.4	3
OE5018R	hypothetical protein	**0.5**	0.2	4	**0.9**	0.1	2	**0.8**	0.1	3
OE5022F	conserved hypothetical protein	**0.4**	0.2	4	**1.0**	0.0	2	**1.1**	0.2	3
OE5029R	hypothetical protein	**0.5**	0.2	4	**1.1**	0.3	2	**0.6**	0.0	3
OE5030R	hypothetical protein	**0.4**	0.1	4	**1.5**	0.2	2			0
OE5031R	conserved hypothetical protein	**0.3**	0.1	4	**0.9**	0.1	3			0
OE5032R	hypothetical protein	**0.4**	0.2	4	**0.9**	0.2	3			0
OE5036F	conserved hypothetical protein	**0.4**	0.1	4	**1.0**	0.2	3	**1.2**	0.0	3
OE5039R	conserved hypothetical protein	**0.4**	0.1	4	**1.3**	0.0	2	**0.9**	0.0	3
OE5043R	conserved hypothetical protein	**0.4**	0.2	4	**0.9**	0.1	2	**1.1**	0.3	3
OE5048F	conserved hypothetical protein	**0.5**	0.1	4	**1.4**	0.3	2	**0.9**	0.1	3
OE5055F	cationic amino acid transport protein homolog	**0.4**	0.0	3	**1.2**	0.2	2	**1.1**	0.1	3
OE5065R	conserved hypothetical protein	**0.3**	0.1	3	**1.2**	0.1	2			0
OE5068F	conserved hypothetical protein	**0.5**	0.0	3	**1.1**	0.1	2	**1.8**	0.4	3
OE5070R	conserved hypothetical protein	**0.4**	0.1	4	**1.7**	0.2	2			0
OE5074R	conserved hypothetical protein	**0.4**	0.1	4	**1.1**	0.2	2	**0.9**	0.1	3
OE5076R	hypothetical protein	**0.4**	0.2	4	**1.0**	0.1	3	**0.8**	0.1	3
OE5078F	sodium-dependent phosphate transport protein homolog	**0.4**	0.1	4	**1.0**	0.1	2	**1.0**	0.1	3
OE5090F	hypothetical protein	**0.4**	0.1	4	**0.9**	0.0	2	**1.6**	0.3	3
OE5093F	hypothetical protein	**0.4**	0.1	4	**1.0**	0.3	2	**0.8**	0.0	3
OE5106F	TrkA domain protein	**0.4**	0.2	4	**0.9**	0.1	2	**0.7**	0.0	3
OE5110F	conserved hypothetical protein	**0.5**	0.1	4	**1.4**	0.6	2	**1.2**	0.4	3
OE5130F	TrkA domain protein	**0.3**	0.1	4	**1.1**	0.0	3			0
OE5134F	probable transport protein	**0.5**	0.3	3	**1.3**	0.3	3	**0.9**	0.4	3
OE5138F	conserved hypothetical protein	**0.3**	0.1	4	**0.9**	0.1	3			0
OE5141R	conserved hypothetical protein	**0.3**	0.1	4	**0.9**	0.1	2	**0.8**	0.1	3
OE5157F	conserved hypothetical protein	**0.5**	0.2	4	**1.0**	0.2	2	**0.9**	0.2	3
OE5158F	hypothetical protein	**0.4**	0.1	4	**0.9**	0.0	3	**1.2**		1
OE5163R	hypothetical protein (overlaps a more likely protein)	**0.4**	0.2	4	**0.9**	0.1	3	**1.1**	0.6	3
OE5165R	hypothetical protein	**0.5**	0.1	4	**1.4**	0.4	2	**0.7**	0.0	3
OE5176R	hypothetical protein	**0.4**	0.1	4	**1.0**	0.1	2	**0.7**	0.1	3

OE5178F	conserved hypothetical protein	**0.4**	0.1	4	**1.3**	0.2	2	**0.9**	0.1	3
OE5188F	protein kinase weak homolog	**0.4**	0.2	4	**1.0**	0.1	2	**1.4**	0.4	3
OE5190R	hydrogenase expression/formation protein homolog	**0.5**	0.2	4	**0.9**	0.1	2	**1.3**	0.2	3
OE5193F	hemolysin homolog	**0.5**	0.2	4	**1.3**	0.4	2	**1.1**	0.1	3
OE5198R	conserved hypothetical protein	**0.4**	0.2	4	**0.9**	0.1	2			0
OE5210F	hypothetical protein	**0.5**	0.2	4	**1.2**	0.1	2	**0.9**	0.1	3
OE5219F	hypothetical protein	**0.4**	0.1	4	**1.1**	0.0	2	**1.4**	0.5	3
OE5222R	hypothetical protein	**0.5**	0.2	4	**0.9**	0.1	3			0
OE5235F	conserved hypothetical protein	**0.4**	0.1	4	**1.0**	0.3	2	**0.8**	0.0	3
OE5249F	conserved hypothetical protein	**0.4**	0.1	4	**0.9**	0.1	2			0
OE5254F	conserved hypothetical protein	**0.5**	0.2	4	**0.9**	0.1	3			0
OE5265F	conserved hypothetical protein	**0.4**	0.1	4	**1.3**	0.3	2	**1.0**	0.2	3
OE5286R	ATP-dependent RNA helicase homolog	**0.4**	0.2	3	**1.0**	0.2	2	**0.9**	0.2	2
OE5296F	hypothetical protein	**0.5**	0.2	4	**1.2**	0.1	2	**0.8**	0.1	3
OE5297F	hypothetical protein	**0.4**	0.1	4	**1.5**	0.3	2			0
OE5303F	predicted orf	**0.5**	0.2	4	**0.8**	0.1	2	**0.8**	0.1	2
OE5308F	conserved hypothetical protein	**0.4**	0.1	4	**1.4**	0.3	2			0
OE5314F	hypothetical protein	**0.4**	0.1	4	**1.1**	0.2	2	**0.9**	0.3	3
OE5315R	hypothetical protein	**0.5**	0.2	4	**0.9**	0.1	2	**0.8**	0.2	3
OE5325F	conserved hypothetical protein	**0.4**	0.1	4	**1.5**	0.3	2	**1.0**	0.2	3
OE5335R	nuclease chain B homolog	**0.5**	0.2	4	**1.0**	0.1	2	**0.8**	0.1	3
OE5345R	hypothetical protein	**0.4**	0.1	4	**0.9**	0.1	3	**0.9**	0.2	3
OE5349R	hypothetical protein	**0.4**	0.1	4	**1.1**	0.2	2			0
OE5350R	conserved hypothetical protein	**0.3**	0.1	4	**1.1**	0.4	3			0
OE5360F	conserved hypothetical protein (nonfunctional, N-terminal part, interrupted by ISH3)	**0.3**	0.1	4	**0.9**	0.1	3	**0.7**	0.1	3
OE5361R	conserved hypothetical protein	**0.4**	0.1	4	**1.0**	0.2	2	**0.8**	0.0	2
OE5365R	conserved hypothetical protein	**0.4**	0.2	4	**0.9**	0.1	2	**0.7**	0.1	3
OE5366R	conserved hypothetical protein	**0.4**	0.1	4	**1.0**	0.2	2			0
OE5386R	deoxyhypusine synthase homolog (nonfunctional, central part)	**0.5**	0.3	4	**0.9**	0.1	2	**1.0**	0.2	3
OE5389F	conserved hypothetical protein	**0.4**	0.2	4	**0.9**	0.3	2	**0.9**	0.1	3
OE5391F	conserved hypothetical protein	**0.5**	0.2	4	**1.0**	0.2	2	**1.1**	0.1	3
OE5394R	conserved hypothetical protein (nonfunctional, N-terminal part, interrupted by ISH2)	**0.4**	0.2	4	**0.9**	0.1	2	**0.8**	0.0	3
OE5396R	hypothetical protein	**0.4**	0.1	4	**0.8**	0.0	3	**0.8**	0.1	3
OE5399R	hypothetical protein	**0.4**	0.2	4	**1.0**	0.3	2	**0.7**	0.0	3
OE5404F	hypothetical protein	**0.3**	0.1	4	**0.9**	0.1	3			0
OE5405F	hypothetical protein	**0.5**	0.2	4	**0.9**	0.2	2	**0.7**	0.1	3
OE5413F	conserved hypothetical protein	**0.5**	0.2	4	**0.9**	0.0	2	**1.2**	0.2	3
OE5417R	conserved hypothetical protein	**0.5**	0.1	4	**1.3**	0.3	2			0
OE5430F	hypothetical protein	**0.5**	0.1	3	**0.9**	0.0	2	**0.7**	0.0	3
OE5434R	conserved hypothetical protein	**0.5**	0.2	4	**0.9**	0.1	2	**0.8**	0.1	3
OE5444F	probable methyltransferase (phosphatidylethanolamine N-methyltransferase homolog)	**0.4**	0.1	4	**0.9**	0.2	2	**0.6**	0.0	3
OE6002R	hypothetical protein	**0.4**	0.1	4	**0.9**	0.2	2			0
OE6005R	hypothetical protein	**0.5**	0.2	4	**1.1**	0.3	3			0
OE6008R_a	ParA domain protein	**0.4**	0.2	4	**1.0**	0.0	2	**0.8**	0.1	3
OE6008R_b	ParA domain protein	**0.3**	0.1	4	**1.0**	0.1	3			0
OE6010F	conserved hypothetical protein	**0.4**	0.1	4	**1.5**	0.7	2	**0.7**	0.1	3
OE6020R	hypothetical protein	**0.5**	0.3	3	**1.0**	0.2	2	**1.0**	0.2	3
OE6022R	hypothetical protein	**0.4**	0.1	4	**1.0**	0.2	2	**0.7**	0.1	3

OE6023R	conserved hypothetical protein	**0.5**	0.1	2	**1.3**	0.4	2			0
OE6027R	hypothetical protein	**0.4**	0.1	4	**1.4**	0.4	2			0
OE6032F	conserved hypothetical protein	**0.4**	0.1	4	**1.2**	0.4	2	**1.3**	0.3	3
OE6038R	hypothetical protein	**0.5**	0.2	4	**0.9**	0.2	3	**1.3**	0.2	3
OE6049R	conserved hypothetical protein	**0.5**	0.2	4	**0.9**	0.2	2	**1.1**	0.1	3
OE6051F	conserved hypothetical protein	**0.5**	0.2	4	**0.9**	0.2	2	**1.2**	0.2	3
OE6052R	hypothetical protein	**0.4**	0.1	4	**1.2**	0.3	2			0
OE6066R	hypothetical protein	**0.4**	0.2	4	**0.9**	0.1	3	**0.9**	0.3	3
OE6069R	conserved hypothetical protein	**0.5**	0.1	4	**1.1**	0.0	2	**0.7**	0.1	3
OE6073R	conserved hypothetical protein	**0.5**	0.2	4	**1.2**	0.5	2	**0.7**	0.1	3
OE6083R	conserved hypothetical protein	**0.5**	0.1	4	**0.9**	0.2	2			0
OE6084R	conserved hypothetical protein	**0.4**	0.1	4	**0.9**	0.0	2	**0.7**	0.1	3
OE6088R	hypothetical protein	**0.4**	0.1	4	**0.9**	0.2	2	**0.6**	0.0	3
OE6092R	hypothetical protein	**0.4**	0.1	4	**1.1**	0.2	2	**0.4**		1
OE6095R	conserved hypothetical protein	**0.5**	0.1	4	**1.2**	0.3	2	**0.6**	0.1	3
OE6097R	conserved hypothetical protein	**0.4**	0.2	4	**0.9**	0.1	3	**0.7**	0.1	3
OE6099F	hypothetical protein	**0.5**	0.2	4	**1.2**	0.1	2	**0.8**	0.1	3
OE6102R	conserved hypothetical protein	**0.4**	0.1	4	**1.0**	0.1	2	**0.9**	0.1	3
OE6104R	conserved hypothetical protein	**0.5**	0.1	4	**0.9**	0.2	2	**0.7**	0.1	3
OE6112F	hypothetical protein	**0.4**	0.2	4	**0.9**	0.2	2	**0.7**	0.0	2
OE6115F	conserved hypothetical protein	**0.5**	0.2	4	**0.9**	0.1	2	**0.8**	0.1	3
OE6118F	hypothetical protein	**0.4**	0.1	4	**1.2**	0.4	2			0
OE6120F	hypothetical protein	**0.4**	0.1	4	**1.0**	0.0	3	**0.8**	0.1	3
OE6121F	hypothetical protein	**0.5**	0.2	4	**0.9**	0.2	3	**0.6**	0.2	3
OE6128R	hypothetical protein	**0.5**	0.1	4	**1.1**	0.1	2	**0.8**		1
OE6138F	conserved hypothetical protein	**0.5**	0.2	4	**1.0**	0.3	2	**0.8**	0.1	3
OE6145R	signal-transducing histidine kinase homolog	**0.5**	0.1	4	**0.9**	0.2	2			0
OE6147R	conserved hypothetical protein	**0.4**	0.1	4	**1.1**	0.2	2	**0.9**	0.2	3
OE6150R	conserved hypothetical protein	**0.5**	0.2	4	**1.0**	0.0	2	**0.9**	0.2	3
OE6152R	conserved hypothetical protein	**0.4**	0.1	4	**1.1**	0.0	2	**0.6**	0.0	3
OE6154F	hypothetical protein	**0.3**	0.1	4	**1.0**	0.1	2	**0.8**	0.1	3
OE6161R	conserved hypothetical protein (nonfunctional, C-terminal part, interrupted by ISH3)	**0.4**	0.2	4	**1.0**	0.2	2	**0.8**	0.1	3
OE6170R	hypothetical protein	**0.4**	0.1	4	**1.2**	0.4	2	**0.6**	0.1	3
OE6278R	protein kinase AfsK homolog	**0.4**	0.1	4	**1.2**	0.3	2			0
OE6280R	hypothetical protein	**0.5**	0.2	4	**1.0**	0.2	2	**0.9**	0.3	3
OE6301F	hypothetical protein	**0.4**	0.1	4	**1.2**	0.5	2	**0.9**	0.1	3
OE6303F	conserved hypothetical protein	**0.4**	0.1	4	**1.1**	0.3	3	**0.8**	0.1	3
OE6315F	conserved hypothetical protein	**0.5**	0.1	4	**1.5**	0.6	2	**0.8**	0.0	2
OE6321R	hypothetical protein	**0.4**	0.1	4	**1.0**	0.0	2			0
OE6323R	hypothetical protein	**0.4**	0.2	4	**1.0**	0.2	2	**0.8**		1
OE6329R	hypothetical protein	**0.5**	0.3	4	**0.9**	0.1	3	**0.9**	0.3	3
OE6340R	conserved hypothetical protein	**0.4**	0.2	4	**0.9**	0.0	2	**0.8**	0.1	3
OE6357F	conserved hypothetical protein	**0.4**	0.1	4	**1.2**	0.4	2	**0.7**	0.1	3
OE7001R	conserved hypothetical protein	**0.4**	0.1	4	**1.0**	0.1	3	**1.1**	0.2	3
OE7009F	conserved hypothetical protein (encoded by ISH7/ISH24 subtype 1)	**0.4**	0.1	4	**0.9**	0.1	3			0
OE7015F	conserved hypothetical protein (nonfunctional, N-terminal part, interrupted by ISH3)	**0.5**	0.2	4	**1.4**	0.6	2	**0.9**	0.2	3
OE7020F	hypothetical protein	**0.4**	0.3	3	**0.9**	0.2	2	**1.0**	0.3	3
OE7043R	predicted orf	**0.5**	0.1	4	**0.9**	0.2	2			0
OE7056F	predicted orf	**0.4**	0.1	4	**1.0**	0.2	2	**0.7**	0.1	3

OE7057F	conserved hypothetical protein	**0.5**	0.1	4	**1.6**	0.4	2	**0.7**	0.1	3
OE7064R	hypothetical protein	**0.4**	0.2	4	**0.9**	0.0	2	**0.9**	0.1	3
OE7070R	conserved hypothetical protein	**0.4**	0.2	4	**0.8**	0.1	2	**1.2**	0.2	3
OE7071F	conserved hypothetical protein	**0.4**	0.1	3	**1.1**	0.1	3	**1.1**	0.1	3
OE7075F	conserved hypothetical protein	**0.5**	0.2	4	**1.1**	0.1	2	**1.5**		1
OE7077F	conserved hypothetical protein	**0.4**	0.1	3	**0.8**	0.1	2	**1.8**	0.1	3
OE7089R	conserved hypothetical protein	**0.5**	0.2	4	**1.0**	0.2	2	**1.7**	0.6	2
OE7091F	hypothetical protein	**0.5**	0.2	4	**1.0**	0.3	2			0
OE7092F	hypothetical protein	**0.4**	0.1	4	**1.7**	0.3	2	**1.6**		1
OE7099R	conserved hypothetical protein	**0.4**	0.2	4	**1.1**	0.2	2			0
OE7100R	ParA domain protein	**0.5**	0.1	2	**0.9**	0.2	3	**1.0**	0.0	2
OE7104R	conserved hypothetical protein	**0.4**	0.1	4	**1.2**	0.3	2	**0.9**	0.1	3
OE7106F	hypothetical protein	**0.4**	0.1	4	**1.9**	0.3	2	**1.4**	0.4	3
OE7108R	conserved hypothetical protein<br>(nonfunctional, C-terminal part, interrupted by ISH2)	**0.5**	0.2	4	**0.9**	0.2	2	**1.2**	0.2	3
OE7111F	conserved hypothetical protein	**0.4**	0.2	4	**1.2**	0.1	2	**1.8**		1
OE7124R	hypothetical protein	**0.5**	0.2	4	**1.1**	0.1	2			0
OE7131R	conserved hypothetical protein	**0.4**	0.1	4	**1.3**	0.4	2			0
OE7135R	hypothetical protein	**0.3**	0.1	3	**1.1**	0.2	2			0
OE7139R	hypothetical protein	**0.4**	0.1	4	**1.3**	0.3	2			0
OE7141R	multidrug resistance transport protein homolog	**0.4**	0.2	4	**1.0**	0.0	2	**1.1**	0.2	3
OE7145R	hypothetical protein	**0.4**	0.2	4	**1.0**	0.1	3	**0.9**	0.1	3
OE7148F	hypothetical protein<br>(nonfunctional, C-terminal part, interrupted by ISH8)	**0.4**	0.1	4	**1.1**	0.1	2			0
OE7150R	conserved hypothetical protein	**0.5**	0.1	4	**0.9**	0.1	2	**1.5**		1
OE7152R	hypothetical protein	**0.5**	0.1	4	**1.1**	0.1	2	**1.2**	0.2	3
OE7154R	hypothetical protein	**0.4**	0.1	4	**1.1**	0.0	2	**0.9**	0.0	3
OE7161R	conserved hypothetical protein	**0.4**	0.1	4	**1.4**	0.4	2	**1.2**	0.3	3
OE7164R	hypothetical protein	**0.4**	0.1	4	**1.1**	0.2	2			0
OE7165R	hypothetical protein	**0.5**	0.3	4	**0.9**	0.1	3	**1.6**	0.3	3
OE7166F	conserved hypothetical protein	**0.4**	0.2	4	**0.9**	0.0	3	**1.0**	0.1	3
OE7167F	conserved hypothetical protein	**0.4**	0.1	4	**1.0**	0.0	3	**1.1**	0.1	3
OE7172F	hypothetical protein	**0.5**	0.1	4	**0.9**	0.1	2	**1.3**	0.0	3
OE7176R	helicase homolog	**0.4**	0.2	4	**1.2**	0.2	2	**1.7**	0.5	3
OE7177F	conserved hypothetical protein	**0.5**	0.2	4	**1.3**	0.2	2	**1.1**	0.2	3
OE7189F	conserved hypothetical protein	**0.3**	0.1	4	**0.9**	0.1	2	**0.8**	0.1	3
OE7190R	hypothetical protein	**0.5**	0.2	4	**0.9**	0.0	2	**1.6**	0.2	3
OE7191F	predicted orf	**0.4**	0.1	4	**1.0**	0.3	2	**0.7**	0.1	3
OE7192F	conserved hypothetical protein	**0.4**	0.1	4	**1.1**	0.1	2			0
OE7195R	predicted orf	**0.4**	0.2	4	**1.0**	0.1	3	**0.9**	0.1	3
OE7196F	conserved hypothetical protein	**0.4**	0.1	4	**1.2**	0.1	2			0
OE7209F	conserved hypothetical protein	**0.4**	0.2	4	**0.9**	0.2	2	**0.7**	0.1	3
OE7212R	hypothetical protein	**0.4**	0.1	4	**1.0**	0.1	2			0
OE7215F	conserved hypothetical protein	**0.4**	0.1	4	**1.3**	0.5	2			0
OE7216F	conserved hypothetical protein	**0.4**	0.2	4	**1.0**	0.1	2	**0.9**	0.1	3
OE7220F	hypothetical protein	**0.3**	0.2	2	**0.9**	0.1	2	**0.9**	0.1	3
OE7223F	conserved hypothetical protein<br>(nonfunctional, N-terminal part, interrupted by ISH2)	**0.3**	0.1	4	**1.1**	0.2	2	**0.6**	0.1	3
OE8007F	hypothetical protein	**0.4**	0.2	4	**0.9**	0.1	3	**1.3**	0.2	3
OE8047F	conserved hypothetical protein	**0.5**	0.2	4	**1.1**	0.1	2	**0.6**	0.1	3
OE8048F	conserved hypothetical protein	**0.4**	0.2	4	**0.9**	0.0	2	**1.2**	0.4	3
orf19a		**0.4**	0.2	3	**0.8**	0.1	3			0

[*1]All genes are tabulated which have a “translational repression factor” of ≤0.5 differentially in exponential phase (lower part) or in stationary phase (one gene on top of the list). The translational repression factor is the quotient of free RNA to polysome-bound RNA (normalized to the average of all genes). If available, also the relative transcript levels of these genes are listed (Quotient stationary phase / exponential phase). ORF numbers and gene products were taken from the genome website www.halolex.mpg.de. The columns ∅, SD and n list average value, standard deviation and number of repetitions.
