# Supplementary material for: Genome-wide analysis of growth phase-dependent translational and transcriptional regulation in halophilic archaea
Source: BMC Genomics. 2007 Nov 12;8:415. doi: 10.1186/1471-2164-8-415 (PMC3225822; doi:10.1186/1471-2164-8-415)
Supplement: Additional file 2 — H. salinarum genes with constitutive non-average translational efficiency [file 1471-2164-8-415-S2.pdf]

Table S2. *H. salinarum* genes with constitutive non-average translational efficiency[*1]

ORF	gene product	translational repression factor exp			translational repression factor stat			transcript level stat/exp		
		∅	SD	n	∅	SD	n	∅	SD	n
**Constitutive overtranslation**										
AA - amino acid metabolism										
OE5206R	carbamate kinase	**0.4**	0.2	4	**0.5**	0.1	3	**1.4**	0.3	3
OE5208R	arginine deiminase	**0.3**	0.1	4	**0.3**	0.0	3	**1.3**	0.1	3
CP - cellular processes										
OE3541R	probable heat shock protein	**0.4**	0.1	4	**0.5**	0.1	3	**4.7**	1.4	3
OE3925R	thermosome beta chain	**0.2**	0.1	4	**0.3**	0.0	3	**0.2**	0.0	3
OE4122R	thermosome alpha chain	**0.2**	0.1	4	**0.4**	0.2	3	**0.2**	0.0	3
OE5212F_a	SMC-like protein Sph1	**0.4**	0.2	4	**0.4**	0.0	3	**2.9**	1.5	3
OE5212F_b	SMC-like protein Sph1	**0.4**	0.2	3	**0.3**	0.1	3	**4.5**	1.9	3
MIS - miscellaneous										
OE2708R	superoxide dismutase 1 (Mn containing)	**0.4**	0.1	4	**0.5**	0.2	3	**0.2**	0.1	3
OE3112R	AAA-type ATPase (transitional ATPase homolog)	**0.3**	0.2	4	**0.5**	0.0	2	**0.6**	0.1	3
OE3356F	AAA-type ATPase (transitional ATPase homolog)	**0.3**	0.2	3	**0.5**	0.0	3	**0.9**	0.1	3
OE5082R	AAA-type ATPase (transitional ATPase homolog)	**0.4**	0.2	4	**0.4**	0.0	3	**0.8**	0.1	3
OE5160F	glycerol dehydrogenase	**0.5**	0.4	4	**0.3**	0.0	3	**1.1**	0.3	3
TC - transcription										
OE1478R	transcription initiation factor TFB	**0.4**	0.2	4	**0.3**	0.1	3	**0.7**	0.1	3
TP - small molecule transport										
OE1781F	probable ABC-type transport system ATP-binding protein	**0.4**	0.1	4	**0.3**	0.1	3			0
CHY - conserved hypothetical protein, HY - hypothetical protein, NOF - no function assigned to experimentally identified protein										
OE1782F	conserved hypothetical protein	**0.4**	0.1	4	**0.5**	0.1	3	**0.7**	0.1	3
OE1783F	conserved hypothetical protein	**0.3**	0.0	4	**0.5**	0.1	3	**0.8**	0.2	3
OE2585R	conserved hypothetical protein	**0.5**	0.1	4	**0.4**	0.1	3			0
orf178-02		**0.5**	0.1	4	**0.3**	0.0	3			0

[*1]All genes are tabulated which have a "translational repression factor" of ≤0.5 in exponential phase and in stationary phase. The translational repression factor is the quotient of free RNA to polysome-bound RNA (normalized to the average of all genes). If available, also the relative transcript levels of these genes are listed (Quotient stationary phase / exponential phase). ORF numbers and gene products were taken from the genome website www.halolex.mpg.de. The columns ∅, SD and n list average value, standard deviation and number of repetitions.
