# Supplementary material for: Genome-wide analysis of growth phase-dependent translational and transcriptional regulation in halophilic archaea
Source: BMC Genomics. 2007 Nov 12;8:415. doi: 10.1186/1471-2164-8-415 (PMC3225822; doi:10.1186/1471-2164-8-415)
Supplement: Additional file 3 — Growth phase dependent transcriptional regulation in Hb. salinarum [file 1471-2164-8-415-S3.pdf]

Table S3. Growth phase dependent transcriptional regulation in *Hb. salinarum*[*1]

ORF	gene product	transcript level stat/exp			translational repression factor exp			translational repression factor stat		
		∅	SD	n	∅	SD	n	∅	SD	n
**Better transcription in stationary growth phase**										
AA - amino acid metabolism										
OE1665R	dihydrodipicolinate synthase	**2.2**	0.4	3	**1.1**	0.3	4	**1.0**	0.1	2
OE2736F	formiminoglutamase	**2.8**	0.4	3	**1.4**	0.3	4	**1.0**	0.1	3
OE3913F	imidazoleglycerol-phosphate synthase (glutamine amidotransferase)	**2.3**	0.9	3	**0.8**	0.1	3	**1.1**	0.1	2
OE4206F	methylaspartate mutase large chain	**2.4**	0.5	2	**1.7**	0.6	3	**0.9**		1
CIM - central intermediary metabolism										
OE3765F	glycerol-3-phosphate dehydrogenase chain C	**3**	1.2	3	**1.2**	0.2	4	**1.1**		1
COM - coenzyme metabolism										
OE1143R	probable molybdenum cofactor biosynthesis protein MoeA2	**2.4**	0.5	2	**1.7**	0.2	3	**1.1**	0.1	2
OE1615R	folylpolyglutamate synthase / dihydropteroate synthase	**2.1**	0.4	3	**1.3**	0.4	4	**1.0**	0.1	3
CP - cellular processes										
OE1482R	probable dolichyl-phosphate beta-glucosyltransferase (only N-terminal homology)	**2.1**	0.4	2	**2.0**	1.1	2	**2.1**		1
OE1515R_a	structural-maintenance-of-chromosomes protein	**3.7**	1.9	3	**0.4**	0.2	4	**1.0**	0.1	2
OE1515R_b	structural-maintenance-of-chromosomes protein	**3.4**	1.1	3	**1.5**	0.4	3	**0.9**	0.1	3
OE1515R_c	structural-maintenance-of-chromosomes protein	**2.9**	1.0	3	**0.4**	0.2	4	**1.2**	0.3	2
OE3541R	probable heat shock protein	**4.7**	1.4	3	**0.4**	0.1	4	**0.5**	0.1	3
OE5037R	cell division control protein Cdc6 homolog	**2.1**	0.6	3	**0.4**	0.1	4	**1.2**		1
OE5212F_a	SMC-like protein Sph1	**4.5**	1.9	3	**0.4**	0.2	3	**0.3**	0.1	3
OE5212F_b	SMC-like protein Sph1	**2.9**	1.5	3	**0.4**	0.2	4	**0.4**	0.0	3
OE6037R	cell division control protein Cdc6 homolog	**3.0**	0.2	3	**0.6**	0.3	4	**1.0**	0.1	2
EM - energy metabolism										
OE2223F	probable dimethylsulfoxide reductase chain A (reductase subunit)	**4.5**	1.8	3	**1.4**		1	**0.8**	0.3	2
OE2225F	dimethylsulfoxide reductase chain B (electron transfer protein)	**5.0**	1.7	3	**1.4**	0.2	3	**1.1**		1
ISH - transposases and ISH-encoded proteins										
OE1031F	probable transposase (ISH10)	**2.4**	0.1	2	**1.5**	0.2	3	**1.1**	0.1	2
OE1045F	transposase homolog (TCE39)	**2.7**	0.6	3	**1.2**	0.2	3	**1.3**		1
LIP - lipid metabolism										
OE3093R	geranylgeranyl-diphosphate geranylgeranyltransferase (phytoene synthase)	**2.4**	0.5	3	**1.3**	0.3	4	**1.1**	0.3	3
MIS - miscellaneous										
OE1462R	nicotinamide-nucleotide adenylyltransferase	**2.3**	0.7	2	**1.7**	0.6	4	**0.9**	0.3	2
OE1656F	indole-3-acetyl-L-aspartic acid hydrolase	**2.2**	0.4	3	**1.0**	0.3	4	**1.0**	0.1	2
OE1698R	probable oxidoreductase Oxr4	**5.5**	0.8	3	**0.4**	0.2	4	**0.5**	0.2	3
OE1808F	probable phytoene dehydrogenase 3	**3.3**	0.7	3	**1.3**	0.4	4	**1.0**		1
OE2546F	probable glycosyltransferase	**2.2**	0.1	2	**1.4**	0.1	4	**0.8**	0.2	3
OE3100F	bacterioopsin-linked protein Blp	**3.2**	0.6	3	**0.6**	0.1	3	**2.5**	0.3	3
OE3261F	conserved cobalamin operon protein	**2.3**	0.2	3	**1.2**	0.4	4	**1.0**	0.1	3
OE4021F	probable oxidoreductase	**2.1**	0.2	2	**1.6**	0.0	2	**1.0**		1
OE4272F	conserved hem operon protein	**2.0**	0.6	3	**0.6**	0.3	4	**0.9**	0.1	2

OE4615F	protein N-acetyltransferase	**2.4**	0.7	3	**1.2**	0.3	4	**1.3**	0.1	2
OE4630R	probable GTP-binding protein	**2.2**	0.4	3	**1.8**	0.6	3	**1.2**	0.3	3
OE5192R	alkaline phosphatase	**2.5**	0.4	3	**0.3**	0.1	4	**1.0**	0.0	3
NUM - nucleotide metabolism										
OE3363F	probable orotidine-5'-phosphate decarboxylase	**2.1**	0.1	3	**1.1**	0.3	4	**1.0**	0.1	3
RMT - RNA maturation										
OE1222R	tRNA adenylyltransferase, CCA-adding	**2.3**	0.7	2	**2.2**	0.2	2	**1.1**	0.1	3
OE3749R	queuine tRNA-ribosyltransferase	**2.3**	0.3	3	**1.4**	0.2	3	**1.0**		1
RRR - replication, repair, recombination										
OE1286R	DNA mismatch repair protein	**3.4**	0.2	3	**1.4**	0.1	3	**1.1**	0.0	2
OE3108F	probable DNA primase	**2.5**	0.6	3	**1.0**	0.2	4	**1.0**	0.1	3
OE3467R	deoxyribodipyrimidine photo-lyase	**2.0**	0.1	3	**1.7**	0.6	3	**0.8**	0.1	3
OE4344F	excinuclease ABC chain C	**2.8**	0.6	3	**1.2**	0.2	4	**1.1**	0.2	3
TC - transcription										
OE1557R	transcription antitermination protein homolog	**2.2**	0.2	3	**1.1**	0.2	3	**1.0**	0.0	2
TL - translation										
OE4132R	tryptophan-tRNA ligase	**2.2**	0.2	3	**2.1**	0.7	3	**1.2**	0.3	3
OE4139R	tyrosine-tRNA ligase	**2.4**	0.8	3	**0.9**	0.4	4	**0.9**	0.2	2
TP - small molecule transport										
OE1288F	probable cationic amino acid transport protein	**2.1**	0.3	3	**1.1**	0.2	4	**1.1**	0.1	3
OE3961R	probable Na+/H+-exchanging protein	**2.2**	0.5	3	**1.2**	0.2	3	**1.0**	0.1	2
OE4082F	probable cation-transporting ATPase	**2.5**	0.3	3	**1.3**	0.1	4	**1.1**	0.2	3
OE4316F	ABC-type transport system permease protein	**2.1**	0.1	3	**0.6**	0.1	4	**1.1**	0.3	3
OE4318F	ABC-type transport system ATP-binding protein	**2.8**	0.9	3	**1.0**	0.1	4			0
OE4358F	ABC-type transport system permease protein	**2.2**	0.3	2	**1.3**	0.1	4	**0.8**	0.2	3
OE4479R	probable ABC-type phosphate transport system ATP-binding protein	**3.5**	0.9	3	**1.1**	0.1	2	**1.1**	0.3	3
OE4485R	probable ABC-type phosphate transport system periplasmic substrate-binding protein	**3.1**	1.0	3	**0.9**	0.2	4	**1.2**	0.1	2
OE5166F	probable ABC-type transport system periplasmic substrate-binding protein (sugar / sn-glycerol-3-phosphate-binding protein)	**10.4**	3.2	3	**0.5**	0.2	4	**1.0**	0.0	2
OE5168F	probable ABC-type transport system permease protein (sugar / sn-glycerol-3-phosphate transport protein)	**4.6**	1.9	3	**0.5**	0.2	4	**0.9**	0.2	2
OE5169F	probable ABC-type transport system permease protein (sugar / sn-glycerol-3-phosphate transport protein)	**4.1**	2.4	3	**0.7**	0.2	3	**0.8**	0.0	2
CHY - conserved hypothetical protein, HY - hypothetical protein, NOF - no function assigned to experimentally identified protein										
OE1008F	hypothetical protein	**2.6**	0.9	3	**0.9**	0.1	4	**1.0**	0.1	3
OE1025F	conserved hypothetical protein	**3.2**	0.7	3	**1.3**	0.2	3	**1.0**	0.2	3
OE1036F	hypothetical protein	**2.9**	0.4	3	**1.6**	0.3	4	**1.0**	0.1	3
OE1037F	hypothetical protein	**3.3**	0.2	3	**2.1**	0.9	3	**0.9**		1
OE1055R	hypothetical protein	**3.0**	0.0	2	**1.7**	0.3	3	**0.9**	0.2	3
OE1192R	hypothetical protein	**5.5**	0.1	2	**1.4**	0.2	4	**1.1**	0.1	2
OE1194R	hypothetical protein	**2.4**	0.2	2	**1.2**	0.2	4	**1.0**	0.1	3
OE1196R	hypothetical protein	**3.5**	2.4	2	**2.7**	2.0	2	**0.9**		1
OE1285F	conserved hypothetical protein	**2.3**	0.3	3	**1.4**	0.3	4	**1.1**	0.1	3
OE1314F	transport protein homolog	**4.3**	1.1	2	**1.6**	0.5	2			0
OE1342R	hypothetical protein	**2.3**	0.2	2	**1.6**	0.4	4	**1.3**		1
OE1417F	conserved hypothetical protein	**2.2**	0.0	2	**1.4**	0.0	3	**1.1**		1
OE1640F	transport protein homolog	**4.0**	1.5	2	**1.3**	0.3	4	**1.0**	0.0	3
OE1738R	hypothetical protein	**2.2**	0.3	3	**1.1**	0.1	3	**1.1**		1
OE1794R	conserved hypothetical protein	**2.7**	0.6	3	**1.3**	0.4	4	**0.6**	0.0	3

OE1841R	HyfD / HycC / NADH dehydrogenase (ubiquinone) chain L homolog	**3.0**	0.4	3	**1.2**	0.2	4	**1.1**	0.1	3
OE1882R	hypothetical protein	**2.9**	0.3	3	**1.0**	0.2	4	**1.3**	0.2	3
OE1899R	conserved hypothetical protein	**3.0**	0.2	3	**1.3**	0.2	3	**1.3**	0.2	2
OE1947F	conserved hypothetical protein	**3.7**	0.9	2	**1.2**	0.1	4	**1.2**	0.1	2
OE1974R	conserved hypothetical protein	**3.0**	0.3	3	**1.1**	0.2	3	**0.8**	0.3	3
OE2052F	conserved hypothetical protein	**2.3**	0.3	3	**1.2**	0.4	4	**1.1**	0.2	3
OE2102R	conserved hypothetical protein	**3.7**	0.9	3	**1.2**	0.1	4	**0.5**	0.1	3
OE2108F	conserved hypothetical protein	**2.0**	0.4	3	**1.3**	0.1	2	**1.1**	0.1	2
OE2232F	conserved hypothetical protein	**2.1**	0.2	2	**2.1**	0.4	2	**0.8**		1
OE2280R	hypothetical protein	**3.1**	0.4	3	**1.5**	0.3	3	**1.2**	0.1	3
OE2288F	pheromone shutdown protein homolog	**2.1**	0.5	3	**1.4**	0.3	3	**1.0**	0.0	3
OE2357F	conserved hypothetical protein	**2.5**	0.4	2	**1.4**	0.2	3	**1.2**		1
OE2365R	hydrolase homolog	**2.2**	0.2	2	**1.4**	0.2	4	**1.0**	0.1	3
OE2430F	hypothetical protein	**2.1**	0.2	2	**1.9**	0.4	3	**1.0**	0.3	3
OE2506R	conserved hypothetical protein	**2.4**	0.7	3	**1.3**	0.3	4	**1.0**	0.1	3
OE2536F	hypothetical protein	**2.5**	0.8	2	**1.5**	0.3	3	**0.8**	0.1	3
OE2537F	sugar transferase homolog	**3.3**	0.6	3	**1.4**	0.1	4	**1.0**	0.1	3
OE2545F	conserved hypothetical protein	**2.1**	0.1	2	**2.1**	0.5	3	**1.1**	0.0	2
OE2741F	hypothetical protein	**2.0**	0.5	2	**1.8**	0.1	3	**1.0**	0.2	3
OE2844R	transcription regulator homolog / TrkA C-terminal domain protein	**2.1**	0.4	3	**1.1**	0.2	4	**1.0**	0.2	3
OE2988R	conserved hypothetical protein	**2.7**	1.1	3	**1.1**	0.1	4	**1.0**	0.3	3
OE3432R	conserved hypothetical protein	**3.1**	0.8	3	**1.3**	0.0	2	**1.0**	0.0	2
OE3445F	conserved hypothetical protein	**2.1**	0.2	3	**1.3**	0.2	3	**1.2**	0.1	3
OE3542R	hypothetical protein	**2.6**	0.3	3	**0.5**	0.1	4	**0.2**	0.0	3
OE3549F	hypothetical protein	**3.0**	1.2	3	**5.5**	2.2	2	**1.2**		1
OE3551R	erythromycin esterase homolog	**3.6**	0.5	3	**1.6**	0.4	4	**1.2**	0.3	2
OE3766R	hypothetical protein	**2.7**	0.6	3	**1.2**	0.1	3	**1.2**	0.1	2
OE3822R	conserved hypothetical protein	**6.0**	2.0	3	**1.0**	0.1	4	**1.1**	0.1	3
OE3949R	glutaredoxin homolog	**2.6**	0.8	3	**1.0**	0.2	4	**1.0**	0.1	2
OE4028R	conserved hypothetical protein	**2.1**	0.4	2	**1.1**	0.1	4	**0.8**	0.0	3
OE4244F	conserved hypothetical protein	**2.1**	0.3	3	**1.3**	0.3	4	**1.1**	0.2	3
OE4349F	hypothetical protein	**2.3**	0.3	2	**1.6**	0.4	4	**0.9**	0.2	3
OE4363F	hypothetical protein	**4.3**	0.8	3	**1.4**	0.1	2	**1.1**	0.1	3
OE4432R	conserved hypothetical protein	**2.4**	0.7	3	**1.2**	0.6	4	**0.9**		1
OE4444F	conserved hypothetical protein	**2.1**	0.5	3	**1.1**	0.2	4	**0.9**	0.1	3
OE5033R	hypothetical protein	**2.2**	0.2	3	**0.5**	0.1	4	**1.1**	0.5	2
OE5174R	hypothetical protein	**2.0**	0.2	3	**0.5**	0.3	4	**0.9**	0.0	2
OE7008F	hypothetical protein (encoded by ISH7/ISH24 subtype 1)	**3.2**	0.5	3	**0.8**	0.2	3	**1.3**	0.5	2
OE7012R	hypothetical protein	**3.6**	0.2	3	**0.6**	0.3	4	**1.3**	0.2	2
OE7079F	tetracyclin resistance protein homolog	**2.5**	1.0	2	**0.6**	0.2	4	**0.9**	0.3	2
OE8009R	conserved hypothetical protein	**2.6**	0.7	3	**0.7**	0.2	3	**1.3**	0.5	2
**Better transcription in exponential growth phase**										
AA - amino acid metabolism										
OE1270F	glutamate dehydrogenase	**0.4**	0.1	3	**0.3**	0.2	4	**0.6**	0.2	3
OE1432F	sarcosine oxidase beta chain	**0.4**	0.1	3	**1.1**	0.4	3	**0.9**	0.0	2
OE2173F	cystathionine gamma-lyase	**0.4**	0.1	3	**0.6**	0.2	4	**0.9**	0.2	3
OE3036F	glycine hydroxymethyltransferase	**0.4**	0.1	3	**0.9**	0.3	4	**0.9**	0.1	3
OE3277R	glycine cleavage system protein H	**0.5**	0.0	3	**0.7**	0.1	4	**0.9**	0.1	3
OE3278R	glycine cleavage system protein T	**0.4**	0.1	3	**0.6**	0.2	4	**0.9**	0.3	3

	(aminomethyltransferase)									
OE3584R	probable muconate cycloisomerase	**0.4**	0.1	3	**0.8**	0.3	4	**1.0**	0.2	3
OE3922R	glutamate-ammonia ligase	**0.3**	0.0	3	**1.5**	0.6	4	**1.1**	0.1	3
OE3931R	threonine dehydratase	**0.4**	0.1	3	**1.2**	0.7	4	**1.4**	0.2	3
OE3959R	branched-chain-amino-acid transaminase	**0.2**	0.0	3	**0.4**	0.1	4	**0.6**	0.2	3
OE4121R	probable ornithine cyclodeaminase	**0.4**	0.1	3	**1.1**	0.5	4	**1.2**		1
OE4159F	adenosylhomocysteinase	**0.5**	0.1	3	**0.4**	0.1	4	**0.5**	0.2	3
CE - cell envelope										
OE1806R	probable periplasmic protein	**0.4**	0.0	3	**0.8**	0.3	4			0
CIM - central intermediary metabolism										
OE1495R	pyruvate kinase	**0.5**	0.2	2	**1.2**	0.3	3	**0.7**	0.0	3
OE1500R	pyruvate, water dikinase (PEP synthase)	**0.2**	0.0	3	**0.4**	0.1	4	**0.9**	0.0	3
OE2019F	fructose-bisphosphate aldolase 1	**0.3**	0.1	3	**0.6**	0.2	4	**1.0**	0.2	3
OE2020F	fructose-bisphosphatase	**0.3**	0.1	3	**1.0**	0.5	4	**1.0**	0.2	3
OE2622R	pyruvate-ferredoxin oxidoreductase beta chain	**0.4**	0.1	3	**0.5**	0.1	4	**0.6**	0.0	3
OE2640F	phosphopyruvate hydratase (enolase)	**0.3**	0.1	3	**0.7**	0.3	4	**1.0**	0.2	3
OE2865R	succinate dehydrogenase chain A (flavoprotein)	**0.4**	0.1	3	**1.1**	0.1	2	**0.7**	0.1	3
OE2866R	succinate dehydrogenase chain B (iron-sulfur protein)	**0.4**	0.0	3	**0.7**	0.2	4	**1.0**	0.2	3
OE2868R	succinate dehydrogenase chain C (cytochrome b-556)	**0.3**	0.0	3	**0.8**	0.3	3	**0.8**	0.0	3
OE3195F	succinate-CoA ligase (ADP-forming) beta chain	**0.3**	0.0	3	**0.3**	0.2	4	**1.0**	0.2	3
OE3634F	isocitrate dehydrogenase (NADP+)	**0.4**	0.0	3	**0.4**	0.1	4	**0.9**	0.2	2
COM - coenzyme metabolism										
OE1146R	probable molybdenum cofactor biosynthesis protein MoeA 1	**0.4**	0.1	3	**1.3**	0.2	2	**0.8**	0.2	3
OE2057F	thiamin biosynthesis protein ThiC	**0.3**	0.0	3	**0.4**	0.1	4	**1.1**	0.1	3
OE3207F	probable precorrin-8W decarboxylase	**0.1**	0.0	3	**0.4**	0.1	4	**0.8**	0.1	3
OE3209F	probable precorrin-2 C20-methyltransferase	**0.2**	0.1	3	**0.4**	0.2	4	**0.8**	0.1	3
OE3212F	precorrin-4 C11-methyltransferase	**0.3**	0.1	3	**0.7**	0.3	4	**0.9**	0.1	3
OE3214F	precorrin-3B C17-methyltransferase 1	**0.4**	0.1	3	**0.8**	0.4	4	**1.1**	0.0	3
OE3216F	precorrin-3B C17-methyltransferase 2	**0.1**	0.0	3	**0.3**	0	4	**1.1**		1
OE3230F	CobN protein (probable cobalt chelatase)	**0.3**	0.0	3	**0.8**	0.4	4	**1.0**	0.2	3
OE3648F	nicotinate-nucleotide pyrophosphorylase (carboxylating)	**0.5**	0.1	3	**1.6**	0.3	4	**1.2**	0.2	3
OE4651F	thiamine biosynthesis protein (thiazole biosynthetic enzyme)	**0.1**	0.0	3	**0.4**	0.1	4	**0.6**	0.1	3
OE4654F	phosphomethylpyrimidine kinase	**0.2**	0.0	3	**0.6**	0.2	4	**0.9**	0.1	3
CP - cellular processes										
OE1559R	cell division protein FtsZ1	**0.5**	0.0	3	**1.1**	0.2	4	**1.0**		1
OE3693F	peptidylprolyl isomerase	**0.5**	0.2	3	**0.4**	0.1	4	**1.0**	0.1	3
OE3925R	thermosome beta chain	**0.2**	0.0	3	**0.2**	0.1	4	**0.3**	0.0	3
OE4122R	thermosome alpha chain	**0.2**	0.0	3	**0.2**	0.1	4	**0.4**	0.2	3
EM - energy metabolism										
OE1953F	NADH dehydrogenase (ubiquinone) chain A	**0.2**	0.1	3	**0.5**	0.1	4	**0.8**	0.2	3
OE1954F_a	NADH dehydrogenase (ubiquinone) chain B	**0.4**	0.1	3	**1.5**	0.3	4	**1.0**	0.3	3
OE1954F_b	NADH dehydrogenase (ubiquinone) chain B	**0.4**	0.1	3	**0.4**	0.2	4	**0.8**	0.3	2
OE1956F	NADH dehydrogenase (ubiquinone) chain CD	**0.4**	0.1	3	**0.7**	0.3	4	**0.8**	0.1	3
OE1957F	NADH dehydrogenase (ubiquinone) chain H	**0.4**	0.0	3	**0.8**	0.2	4	**1.0**	0.4	3
OE1958F	NADH dehydrogenase (ubiquinone) chain I	**0.3**	0.1	3	**0.6**	0.1	4	**1.0**	0.3	3
OE1960F	NADH dehydrogenase (ubiquinone) chain J2 (C-terminal homology)	**0.3**	0.1	3	**0.7**	0.1	4	**0.8**	0.1	3
OE3985R	H+-transporting two-sector ATPase chain A.a	**0.2**	0.0	3	**0.5**	0.1	4	**1.1**	0.3	3
OE3986R	H+-transporting two-sector ATPase chain F.a	**0.2**	0.0	3	**0.5**	0.1	4	**2.0**		1
OE3987R	H+-transporting two-sector ATPase chain C.a	**0.2**	0.1	3	**0.5**	0.2	4	**0.9**	0.1	3

OE3988R	H+-transporting two-sector ATPase chain E.a	**0.2**	0.0	3	**0.5**	0.2	4	**1.1**		1
OE3989R	H+-transporting two-sector ATPase chain K.a	**0.1**	0.0	3	**0.5**	0.1	4	**1.1**	0.1	3
OE3991R	H+-transporting two-sector ATPase chain I.a	**0.2**	0.0	3	**0.8**	0.2	4	**0.9**	0.2	3
OE3992R	H+-transporting two-sector ATPase chain H.a	**0.1**	0.1	3	**0.6**	0.1	4	**1.2**	0.3	3
OE4005F	electron transfer flavoprotein beta chain	**0.4**	0.0	3	**0.6**	0.3	4	**1.0**	0.3	2
OE4007F	electron transfer flavoprotein alpha chain	**0.4**	0.1	3	**0.9**	0.4	4	**1.0**		1
OE4073R	halocyanin HcpB	**0.4**	0.1	3	**1.2**	0.1	2	**0.8**	0.1	3
OE4075R	halocyanin HcpB	**0.4**	0.1	3	**1.4**	0.5	4	**0.9**	0.0	2
OE4217R	ferredoxin (2Fe-2S)	**0.2**	0.0	3	**0.6**	0.1	4	**1.0**	0.2	3
ISH - transposases and ISH-encoded proteins										
OE1073F	probable IS200-type transposase (ISH12)	**0.3**	0.1	3	**1.3**	0.7	4	**1.7**	0.6	3
LIP - lipid metabolism										
OE1271F	probable fatty-acid-CoA ligase	**0.4**	0.0	3	**1.9**	0.6	3	**1.2**	0.1	3
OE2912F	probable fatty-acid-CoA ligase	**0.4**	0.1	3	**0.8**	0.1	4	**0.9**	0.2	3
OE3637R	hydroxymethylglutaryl-CoA reductase (NADPH)	**0.5**	0.1	3	**1.0**	0.2	4	**1.0**	0.1	3
OE4500R	probable acyl/butyryl-CoA dehydrogenase	**0.5**	0.1	3	**0.4**	0.2	4	**0.9**	0.0	2
MIS - miscellaneous										
OE1081R	probable glycosyltransferase	**0.4**	0.1	3	**1.3**	0.3	3	**0.7**	0.1	2
OE1171F	type I restriction-modification system DNA-methyltransferase RmeM (nonfunctional, N-terminal part, interrupted by ISH8)	**0.5**	0.1	2	**2.2**	1.5	2	**0.7**		1
OE1371R	prefoldin beta chain	**0.4**	0.0	3	**0.5**	0.1	4	**0.9**	0.1	3
OE1407F	inorganic pyrophosphatase	**0.3**	0.0	3	**0.5**	0.2	4	**1.2**	0.2	3
OE1465F	endopeptidase La	**0.2**	0.0	3	**0.6**	0.2	4	**0.9**	0.1	3
OE1613R	probable acylaminoacyl-peptidase	**0.4**	0.2	2	**0.9**	0.4	4			0
OE1684F	probable sulfate adenylyltransferase small chain	**0.3**	0.1	3	**0.8**	0.2	4	**0.9**	0.3	3
OE1934R	proteinase IV	**0.3**	0.1	3	**1.2**	0.2	4			0
OE2015R	3-hydroxybutyryl-CoA dehydrogenase	**0.4**	0.1	3	**0.7**	0.1	4	**1.1**	0.2	3
OE2373F	probable phosphate acetyltransferase	**0.4**	0.0	3	**0.7**	0.1	4	**1.0**	0.0	2
OE2390R	flagella accessory protein	**0.5**	0.1	3	**1.2**	0.2	3	**1.1**	0.5	2
OE2451R	probable oxidoreductase	**0.4**	0.1	3	**0.7**	0.2	4	**0.9**	0.1	3
OE2708R	superoxide dismutase 1 (Mn containing)	**0.2**	0.1	3	**0.4**	0.1	4	**0.5**	0.2	3
OE2853R	probable peptidylprolyl isomerase	**0.3**	0.0	3	**0.4**	0.2	4	**0.9**	0.1	3
OE2906R	superoxide dismutase 2	**0.3**	0.1	3	**0.8**	0.1	4	**0.7**	0.1	3
OE3168R	pyridoxal phosphate-dependent aminotransferase (acetylornithine transaminase homolog)	**0.3**	0.0	3	**0.6**	0.2	4	**0.8**	0.2	3
OE3213F	CbiG protein	**0.1**	0.1	3	**0.5**	0.2	4	**1.4**	0.1	2
OE3218F	cobalamin operon protein	**0.2**	0.1	3	**0.3**	0.1	4	**0.9**	0.2	3
OE3221F	conserved cobalamin operon protein	**0.1**	0.0	3	**0.7**	0.3	4	**1.1**	0.2	3
OE3224F	conserved cobalamin operon protein	**0.2**	0.0	3	**0.5**	0.4	4	**0.8**	0.1	3
OE3807R	oligoendopeptidase	**0.3**	0.0	3	**0.6**	0.3	4	**1.0**	0.1	3
OE4113F	2-oxoacid dehydrogenase E1 component alpha-1 chain (the substrate is NOT pyruvate or 2-oxoglutarate)	**0.3**	0.0	3	**1.1**	0.1	2	**0.7**	0.1	3
OE4114F	2-oxoacid dehydrogenase E1 component beta chain (the substrate is NOT pyruvate or 2-oxoglutarate)	**0.3**	0.1	3	**2.0**	0.3	3	**1.3**		1
OE4116F	dihydrolipoamide dehydrogenase (probable glycine cleavage system protein L and probable E3 component of the 2-oxoacid dehydrogenase complex) (substrate of 2-oxoacid dehydrogenase complex is NOT pyruvate or 2-oxoglutarate)	**0.4**	0.1	3	**1.0**	0.2	4	**0.8**		1
OE4187R	probable DNA-binding protein	**0.2**	0.1	3	**2.6**	1.1	4	**1.4**	0.2	2
OE4230F	probable acylaminoacyl-peptidase	**0.5**	0.1	3	**1.3**	0.5	4	**1.1**	0.2	3
OE4438F	leucyl aminopeptidase	**0.3**	0.1	3	**0.4**	0.2	4	**1.2**	0.2	3
OE4456R	prefoldin alpha chain	**0.4**	0.0	3	**0.6**	0.1	3	**0.9**	0.1	2

MOT - motility										
OE2469F	flagellin A1 precursor	**0.5**	0.2	3	**1.0**	0.1	4	**0.9**	0.2	3
NUM - nucleotide metabolism										
OE2579F	adenylosuccinate synthase	**0.5**	0.2	3	**0.9**	0.3	3	**0.9**	0.1	3
OE3017R	UDP-sugar hydrolase / 5'-nucleotidase	**0.2**	0.0	3	**0.9**	0.1	4	**1.2**	0.1	3
OE3328R	ribonucleotide reductase, B12-dependent	**0.4**	0.0	3	**1.0**	0.3	4	**0.9**	0.2	3
OE3731R	phosphoribosylformylglycinamidine synthase component I	**0.4**	0.0	2	**2.0**	0.2	2	**0.9**	0.2	2
OE4345R	probable ribonucleoside-diphosphate reductase small chain	**0.3**	0.0	3	**1.2**	0.1	4	**0.9**	0.1	3
OE4346R	ribonucleoside-diphosphate reductase large chain	**0.4**	0.1	3	**1.2**	0.2	4	**0.8**	0.1	3
OE5201F	aspartate carbamoyltransferase catalytic chain	**0.2**	0.1	2	**0.3**	0.1	4	**1.1**	0.4	2
OE5202F	aspartate carbamoyltransferase regulatory chain	**0.2**	0.0	3	**0.2**	0.1	4	**1.0**	0.3	2
RRR - replication, repair, recombination										
OE4195F	replication factor C small chain	**0.5**	0.1	3	**0.9**	0.2	4	**0.7**	0.1	3
OE4466R	DNA repair protein	**0.4**	0.1	3	**0.3**	0.1	4	**0.6**	0.2	3
SEC - protein secretion										
OE3787R	preprotein-export translocase chain SecF	**0.5**	0.1	2	**1.5**	0.4	4	**0.8**		1
OE4181R	Sec-independent protein translocase component TatC2	**0.5**	0.2	2	**1.5**	0.3	3	**0.8**	0.1	3
SIG - signal transduction										
OE3436R	transducer protein Htr17	**0.5**	0.1	3	**1.3**	0.2	2	**0.8**	0.1	3
OE3481R	transducer protein HtrII	**0.5**	0.1	3	**0.9**	0.2	4	**0.9**	0.3	3
OE3612R	chemotactic signal transduction system periplasmic substrate-binding protein BasB	**0.4**	0.1	3	**1.0**	0.3	4	**1.1**	0.1	2
OE3854R	probable response regulator	**0.4**	0.1	2	**1.4**	0.2	4	**0.9**	0.2	3
OE5243F	transducer protein Car	**0.4**	0.1	3	**0.3**	0.2	4	**0.7**	0.1	2
TC - transcription										
OE1637R	probable DNA-directed RNA polymerase chain M	**0.5**	0.0	3	**1.0**	0.2	4	**1**	0.3	3
OE2631F	DNA-directed RNA polymerase chain D	**0.3**	0.1	3	**0.4**	0.2	4	**0.9**	0.0	3
OE2638F	DNA-directed RNA polymerase chain K	**0.3**	0.1	3	**0.4**	0.0	4	**1.1**		1
OE3452F	probable DNA-directed RNA polymerase chain M	**0.4**	0.0	3	**1.4**	0.2	2	**0.8**	0.2	3
OE4740R	DNA-directed RNA polymerase chain A'	**0.4**	0.1	3	**0.2**	0.0	3	**0.7**	0.1	3
OE4741R	DNA-directed RNA polymerase chain B'	**0.4**	0.1	3	**0.5**	0.1	4	**0.8**		1
OE4742R	DNA-directed RNA polymerase chain B''	**0.5**	0.0	3	**0.3**	0.1	4	**1.1**		1
OE4743R	DNA-directed RNA polymerase chain H	**0.3**	0.0	3	**0.4**	0.1	4	**0.7**	0.2	3
TL - translation										
OE1160R	ribosomal protein L10.eR	**0.3**	0.0	3	**0.5**	0.1	4	**1.0**	0.5	2
OE1373R	ribosomal protein L37a.eR	**0.5**	0.1	3	**0.8**	0.1	4	**1.7**		1
OE2165R	ribosomal protein S15	**0.2**	0.1	3	**0.4**	0.1	4	**1.2**	0.1	2
OE2601R	ribosomal protein L10	**0.2**	0.0	3	**0.4**	0.2	4	**1.0**	0.2	3
OE2602R	ribosomal protein L1	**0.2**	0.1	3	**0.4**	0.2	4	**1.0**	0.0	3
OE2603R	ribosomal protein L11	**0.4**	0.1	3	**0.8**	0.2	4	**1.1**	0.1	3
OE2627F	ribosomal protein S13	**0.3**	0.1	3	**0.2**	0.1	4	**1.2**		1
OE2628F	ribosomal protein S4	**0.2**	0.0	3	**0.7**	0.2	4	**1.1**		1
OE2629F	ribosomal protein S11	**0.2**	0.0	3	**0.2**	0.1	4	**1.0**	0.7	2
OE2632F	ribosomal protein L18.eR	**0.2**	0.0	3	**0.5**	0.2	4	**0.9**	0.2	3
OE2633F	ribosomal protein L13	**0.2**	0.1	3	**0.5**	0.1	4	**1.3**	0.3	3
OE2641F	ribosomal protein S2	**0.2**	0.0	3	**0.4**	0.1	4	**0.9**	0.1	3
OE2664F	ribosomal protein S28.eR	**0.5**	0.1	3	**0.4**	0.1	4	**1.1**	0.2	3
OE2665F	ribosomal protein L24.eR	**0.4**	0.0	3	**0.7**	0.1	4	**1.1**	0.1	3
OE2679R	ribosomal protein L21.eR	**0.4**	0.1	3	**0.8**	0.2	4	**1.1**	0.2	3
OE2683R	translation elongation factor aEF-1 beta chain	**0.5**	0.0	3	**1.1**	0.2	4	**1.0**	0.3	3

OE3062F	ribosomal protein S17.eR	**0.5**	0.1	2	**1.7**	0.4	3	**0.9**	0.1	2
OE3141R	ribosomal protein L37.eR	**0.5**	0.0	2	**2.3**	1.4	2			0
OE3357R	ribosomal protein S8.eR	**0.2**	0.0	3	**0.7**	0.1	3	**1.0**	0.2	2
OE3390F	ribosomal protein L23	**0.2**	0.0	3	**0.4**	0.0	4	**1.5**		1
OE3392F	ribosomal protein L2	**0.2**	0.1	3	**0.3**	0.0	4	**0.9**	0.2	3
OE3393F	ribosomal protein S19	**0.2**	0.1	3	**0.2**	0.0	4	**1.0**	0.1	3
OE3394F	ribosomal protein L22	**0.2**	0.0	3	**0.2**	0.0	4	**1.2**	0.2	2
OE3395F	ribosomal protein S3	**0.1**	0.0	3	**0.4**	0.2	4	**0.8**	0.1	3
OE3396F	ribosomal protein L29	**0.3**	0.0	3	**0.3**	0.1	4	**1.1**	0.1	3
OE3400F	ribosomal protein S17	**0.1**	0.0	3			0	**1.0**		1
OE3402F	ribosomal protein L14	**0.2**	0.1	3	**0.3**	0.1	4	**0.9**	0.2	3
OE3405F	ribosomal protein S4.eR	**0.1**	0.0	2	**0.4**	0.1	4	**1.0**	0.4	2
OE3408F	ribosomal protein S14	**0.3**	0.0	3	**0.6**	0.2	4	**0.9**	0.2	3
OE3411F	ribosomal protein L6	**0.1**	0.0	3	**0.2**	0.0	4	**0.9**	0.2	2
OE3412F	ribosomal protein L32.eR	**0.2**	0.1	3	**0.2**	0.0	4	**1.0**	0.2	2
OE3413F	ribosomal protein L19.eR	**0.2**	0.1	3	**0.3**	0.1	4	**1.0**	0.0	2
OE3414F	ribosomal protein L18	**0.2**	0.0	3	**0.4**	0.2	4	**1.0**	0.1	3
OE3415F	ribosomal protein S5	**0.4**	0.1	2	**1.0**	0.4	4	**1.0**	0.0	2
OE3416F	ribosomal protein L30	**0.3**	0.0	3	**0.2**	0.1	4	**1.1**	0.1	2
OE3417F	ribosomal protein L15	**0.4**	0.1	3	**0.4**	0.1	4	**1.4**		1
OE3487R	translation initiation factor aIF-5A	**0.4**	0.0	3	**0.4**	0.1	4	**1.0**	0.2	3
OE3817R	ribosomal protein S19.eR	**0.4**	0.0	3	**0.8**	0.2	4	**0.9**	0.1	3
OE3898F	ribosomal protein L40.eR	**0.4**	0.1	2	**1.3**	0.2	3	**1.2**	0.0	2
OE4460R	ribosomal protein L39.eR	**0.3**	0.1	3	**0.3**	0.1	3	**0.9**	0.2	3
OE4507F	phenylalanine-tRNA ligase beta chain	**0.5**	0.1	3	**1.1**	0.2	4	**1.7**	0.0	2
OE4720R	ribosomal protein S10	**0.2**	0.0	3	**0.7**	0.2	4	**1.0**	0.1	3
OE4721R	translation elongation factor aEF-1 alpha chain	**0.2**	0.0	3	**0.3**	0.1	4	**0.5**	0.1	3
OE4729R	translation elongation factor aEF-2	**0.4**	0.1	3	**0.6**	0.2	4	**0.9**	0.0	3
OE4736R	ribosomal protein S12	**0.3**	0.0	3	**0.4**	0.1	4	**0.9**	0.3	2
TP - small molecule transport										
OE1080F	probable polysaccharide export protein	**0.4**	0.1	2	**1.4**	0.3	2	**1.0**	0.1	2
OE2779F	amino acid transport protein (probable phenylalanine transport protein)	**0.3**	0.0	3	**1.2**		1	**1.5**		1
OE3318R	cobalt transport protein CbiN	**0.3**	0.1	3	**0.9**	0.1	4	**0.9**	0.1	3
OE3319R	CbiM protein (probable ABC-type cobalt transport system permease protein 2)	**0.1**	0.0	3	**0.9**	0.1	4	**1.1**	0.3	2
OE3907R	ABC-type transport system permease protein	**0.4**	0.1	2	**1.2**	0.3	4	**1.4**		1
OE3908R	ABC-type transport system ATP-binding protein	**0.4**	0.0	3	**0.7**	0.3	4	**0.8**	0.1	3
OE3910R	probable ABC-type transport system periplasmic substrate-binding protein	**0.4**	0.2	3	**0.3**	0.1	4	**1.0**	0.0	2
OE4301R	ABC-type transport system ATP-binding protein	**0.2**	0.0	3	**0.4**	0.2	4	**0.9**	0.1	3
OE4302R	ABC-type oligopeptide transport system ATP-binding protein	**0.1**	0.0	3	**0.4**	0.2	4	**1.1**	0.2	2
OE4303R	ABC-type transport system permease protein	**0.2**	0.1	3	**0.6**	0.1	4	**1.0**	0.2	3
OE4304R	ABC-type transport system permease protein	**0.1**	0.0	3	**0.9**	0.2	4	**0.8**		1
OE4336R	probable ABC-type transport system permease protein	**0.3**	0.2	3	**0.7**	0.3	4	**0.6**	0.2	2
OE4339R	ABC-type transport system ATP-binding protein	**0.3**	0.0	3	**0.9**	0.3	4	**0.8**	0.1	3
OE4480R	probable ABC-type phosphate transport system permease protein	**0.5**	0.0	3	**1.6**	0.3	4	**1.7**	0.2	3
CHY - conserved hypothetical protein, HY - hypothetical protein, NOF - no function assigned to experimentally identified protein										
OE1001F	conserved hypothetical protein	**0.4**	0.0	3	**0.8**	0.3	3	**0.9**	0.0	3
OE1321R	conserved hypothetical protein	**0.4**	0.2	2	**1.3**	0.0	4	**1.0**	0.2	3

OE1323R	conserved hypothetical protein	**0.4**	0.1	3	**1.8**	0.5	4	**1.1**	0.3	3
OE1352F	hypothetical protein	**0.4**	0.2	3	**0.7**	0.2	4			0
OE1353F	hypothetical protein	**0.4**	0.1	3	**0.8**	0.4	4	**1.1**	0.1	3
OE1356F	conserved hypothetical protein	**0.4**	0.1	3	**0.9**	0.3	4	**1.0**	0.2	3
OE1405R	conserved hypothetical protein	**0.3**	0.0	3	**2.4**	0.9	4	**5.1**	0.2	3
OE1412F	conserved hypothetical protein	**0.4**	0.1	3	**1.1**	0.3	4	**1.2**	0.4	3
OE1475F	conserved hypothetical protein	**0.5**	0.0	3	**0.9**	0.1	4	**0.8**	0.1	3
OE1636F	protein-L-isoaspartate O-methyltransferase homolog	**0.4**	0.1	3	**1.7**	0.3	3	**0.9**		1
OE1718R	conserved hypothetical protein	**0.5**	0.1	3	**1.2**	0.2	4	**0.9**	0.3	3
OE1804R	conserved hypothetical protein	**0.4**	0.0	3	**1.5**	0.2	3	**1.1**	0.1	2
OE1826R	predicted orf	**0.5**	0.1	3	**1.1**	0.2	2	**1.2**	0.1	2
OE1842R	NADH dehydrogenase (ubiquinone) chain L homolog	**0.4**	0.1	2	**1.6**	0.2	3	**1.0**	0.0	2
OE1860F	conserved hypothetical protein	**0.5**	0.1	3	**1.4**		1	**0.8**		1
OE1866F	hypothetical protein	**0.3**	0.0	3	**1.4**	0.2	3	**1.4**		1
OE1878R	hypothetical protein	**0.5**	0.0	3	**0.9**	0.2	4	**0.5**	0.1	3
OE1932R	conserved hypothetical protein	**0.5**	0.1	2	**1.5**	0.3	4	**0.7**		1
OE2131F	conserved hypothetical protein	**0.5**	0.0	3	**1.7**	0.4	4	**1.5**	0.1	3
OE2141F	conserved hypothetical protein	**0.5**	0.0	3	**0.8**	0.3	3	**1.0**		1
OE2160R	hypothetical protein	**0.3**	0.1	3	**0.3**	0.1	4	**0.8**	0.2	3
OE2161R	conserved hypothetical protein	**0.3**	0.1	3	**0.9**	0.3	4	**1.0**	0.1	3
OE2247R	conserved hypothetical protein	**0.5**	0.1	3	**1.1**	0.3	4	**1.1**	0.2	3
OE2249R	transducer protein weak homolog lacking transduction domain	**0.5**	0.0	3	**0.6**	0.2	4	**0.7**	0.0	3
OE2448F	transcription regulator Bat homolog	**0.4**	0.0	3	**0.8**	0.2	4	**1.0**		1
OE2466F	hypothetical protein	**0.4**	0.1	3	**1.5**	0.3	3	**0.7**	0.0	2
OE2525R	conserved hypothetical protein	**0.5**	0.1	2	**1.8**	0.4	4	**1.2**		1
OE2532R	conserved hypothetical protein	**0.4**	0.1	3	**1.2**	0.6	2	**0.9**	0.2	2
OE2573F	hypothetical protein	**0.4**	0.1	3	**0.7**	0.1	4	**0.8**	0.1	3
OE2706R	hypothetical protein	**0.3**	0.1	3	**1.2**	0.2	3	**1.1**		1
OE2716R	hypothetical protein	**0.5**	0.1	3	**1.3**	0.7	3	**1.1**	0.2	2
OE2747R	conserved hypothetical protein	**0.4**	0.1	3	**2.0**	0.9	3	**1.0**		1
OE2778R	hypothetical protein	**0.5**	0.2	3	**1.9**	0.5	3	**0.9**	0.1	3
OE2816F	conserved hypothetical protein	**0.4**	0.0	3	**0.8**	0.3	3	**0.9**	0.1	3
OE2838R	TrkA domain protein	**0.5**	0.1	3	**1.3**	0.2	2	**0.8**	0.1	3
OE2851R	hypothetical protein	**0.3**	0.0	3	**0.8**	0.1	4	**1.4**	0.2	2
OE3028R	conserved hypothetical protein	**0.3**	0.0	3	**0.6**	0.1	4	**0.8**	0.1	3
OE3047F	hypothetical protein	**0.5**	0.1	3	**1.4**	0.3	4	**1.0**	0.1	3
OE3155R	conserved hypothetical protein	**0.4**	0.0	2	**0.8**	0.1	4	**0.8**	0.0	3
OE3227F	nicotinate-nucleotide-dimethylbenzimidazole phosphoribosyltransferase homolog	**0.4**	0.1	3	**0.9**	0.3	4	**1.0**	0.3	2
OE3322F	conserved hypothetical protein	**0.4**	0.0	3	**0.6**	0.2	4	**0.9**		1
OE3361F	conserved hypothetical protein	**0.5**	0.0	2	**1.6**	0.5	2	**1.4**	0.4	3
OE3398F	conserved hypothetical protein	**0.1**	0.0	3	**0.2**	0.0	4	**1.0**	0.2	2
OE3538R	hypothetical protein	**0.5**	0.1	2	**3.5**	2.0	4	**0.9**	0.1	2
OE3569R	hypothetical protein	**0.4**	0.1	2	**1.1**	0.4	3	**1.3**		1
OE3636F	plasmid replication protein RepJ homolog	**0.5**	0.1	3	**1.4**	0.3	4	**1.1**		1
OE3719F	conserved hypothetical protein	**0.4**	0.1	3	**0.7**	0.1	4	**0.8**	0.1	3
OE3796R	conserved hypothetical protein	**0.4**	0.1	3	**0.9**	0.1	3	**1.2**		1
OE3798R	conserved hypothetical protein	**0.4**	0.0	2	**1.0**	0.0	3	**1.8**		1
OE3832F	conserved hypothetical protein	**0.4**	0.0	3	**0.7**	0.2	4	**0.7**	0.1	3
OE3921F	conserved hypothetical protein	**0.4**	0.0	3	**1.5**	0.3	3	**0.8**	0.0	3
OE3942R	hypothetical protein	**0.5**	0.1	3	**1.3**	0.2	3	**1.2**		1

OE4089R	conserved hypothetical protein	**0.5**	0.1	2	**1.9**	0.7	2	**0.9**	0.1	3
OE4201R	preflagellin peptidase homolog	**0.4**	0.0	2	**1.3**	0.2	3	**1.1**	0.3	3
OE4223R	conserved hypothetical protein	**0.3**	0.1	3	**1.3**	0.0	3	**1.3**	0.1	2
OE4300R	hypothetical protein	**0.3**	0.1	3	**0.7**	0.1	4	**0.8**	0.1	3
OE4354R	conserved hypothetical protein	**0.4**	0.0	3	**0.5**	0.2	4	**0.6**	0.0	3
OE4511R	hypothetical protein	**0.1**	0.0	3	**9.0**	3.8	4	**1.2**	0.2	3
OE4563F	hypothetical protein	**0.2**	0.0	3	**0.9**	0.3	4	**1.0**	0.1	3
OE4655R	conserved hypothetical protein	**0.5**	0.1	3	**1.0**	0.4	3			0
OE4670F	conserved hypothetical protein	**0.4**	0.1	3	**1.5**	0.2	4	**0.8**		1
OE4712F	conserved hypothetical protein	**0.5**	0.2	3	**0.7**	0.0	4	**0.9**	0.2	2
OE4716R	conserved hypothetical protein	**0.5**	0.0	3	**0.7**	0.1	4	**0.9**	0.2	3
OE5051R	TrkA domain protein	**0.4**	0.2	3	**1.4**	0.3	4	**0.9**		1
OE5100R	TrkA domain protein	**0.4**	0.0	3	**0.9**	0.2	3	**0.9**	0.2	3
OE5130F	TrkA domain protein	**0.5**	0.0	3	**0.9**	0.1	4	**0.7**	0.1	3
OE5298F	TrkA domain protein	**0.5**	0.1	3	**0.8**	0.1	4	**0.7**	0.1	3

*1 Relative mRNA levels stationary/exponential growth phase are listed for genes reaching values ≥2 or ≤0.5 ("transcript level"). If available, also the translational repression factors for these genes are listed. ORF numbers and gene products correspond to http://www.halolex.mpg.de. The columns ∅, SD and n list average value, standard deviation and number of repetitions.
