# Supplementary material for: Genome-wide analysis of growth phase-dependent translational and transcriptional regulation in halophilic archaea
Source: BMC Genomics. 2007 Nov 12;8:415. doi: 10.1186/1471-2164-8-415 (PMC3225822; doi:10.1186/1471-2164-8-415)
Supplement: Additional file 4 — H. volcanii genes with growth phase-dependent above-average translational efficiency [file 1471-2164-8-415-S4.pdf]

Table S4. *H. volcanii* genes with growth phase-dependent above-average translational efficiency[*1]

identifier	putative function	length (bp)	translational repression factor					
			exp			stat		
			∅	SD	n	∅	SD	n
**Exponential growth phase overtranslation**								
AA - amino acid metabolism								
432H12	aspartokinase LysC	901	**0.3**	0.3	3	**0.7**	0.3	3
437F10	beta-eliminating lyase TnaA	1267	**0.2**	0.1	4	**0.6**	0.3	3
441B11	IGP synthase cyclase subunit HisF	678	**0.5**	0.1	4	**1.5**	0.5	2
	NADPH:quinone oxidoreductase Qor	582						
441C11	aspartokinase LysC	729	**0.4**	0.3	2	**1.2**	0.6	3
441F5	D-3-phosphoglycerate dehydrogenase SerA	796	**0.5**	0.1	4	**0.9**	0.2	3
	conserved hypothetical protein	666						
446B6	O-acetylserine lyase CysM	909	**0.1**	0.0	2	**1.0**	0.4	2
447F4	aminotransferase, class V superfamily Csd	761	**0.3**	0.1	4	**0.6**	0.2	3
448H10	glutamate synthase large subunit GltA	1469	**0.3**	0.1	3	**0.6**	0.2	3
452H2	glutamate dehydrogenase GdhB	2628	**0.4**	0.1	3	**0.8**	0.1	2
454E4	argininosuccinate synthase ArgG	1068	**0.5**	0.1	4	**0.9**	0.3	3
454G5	serine hydroxymethyltransferase GlyA	1068	**0.5**	0.2	4	**1.1**	0.4	3
CHM - carbohydrate metabolism								
445G2	polysaccharide deacetylase domain protein	218	**0.5**	0.2	2	**0.9**	0.2	3
454B11	acetoin dehydrogenase AcoA	855	**0.1**	0.1	2	**0.4**	0.2	3
CIM - central intermediary metabolism								
441D1	NAD-dependent malate dehydrogenase Mdh	1604	**0.4**	0.1	3	**1.0**	0.6	3
443B7	succinyl-CoA synthase, alpha subunit SucD	745	**0.4**	0.0	4	**0.7**	0.3	3
	succinyl-CoA synthase, beta subunit SucC	741						
449B11	pyruvate ferredoxin oxidoreductase subunit alpha PorA	1626	**0.3**	0.1	2	**0.6**	0.2	3
COM - coenzyme metabolism								
444F11	isochorismatase family protein family	944	**0.4**	0.1	4	**0.8**	0.3	3
CP - cellular processes								
454B5	cell division protein FtsH	1216	**0.4**	0.1	3	**0.8**	0.2	3
456H3	ATP-dependent protease, putative	1561	**0.5**	0.0	4	**1.0**	0.6	3
457F11	peptidylprolyl isomerase PpiB2	254	**0.4**	0.1	2	**0.7**	0.3	3
	tellurite resistance protein TehB	197						
EM - energy metabolism								
433C1	cytochrome c oxidase subunit I-like protein CtaD	1159	**0.5**	0.2	4	**0.9**	0.3	3
434B6	H+-transporting ATP synthase subunit I AtpI	1027	**0.4**	0.1	4	**0.8**	0.4	3
	hypothetical protein	519						
LIP - lipid metabolism								
447D1	3-hydroxybutyryl-CoA dehydrogenase Hbd1	810	**0.3**	0.1	3	**0.8**	0.3	3
MIS - miscellaneous								
438B7	phospho-adenylyl-sulfate Reductase NodP	835	**0.3**	0.1	3	**0.5**	0.1	3
	phospho-adenylyl-sulfate Reductase NodP	735						
449F5	dehydrogenase homolog	115	**0.4**	0.1	2	**1.3**	0.5	2
457F5	oxidoreductase Aad	706	**0.4**	0.1	3	**0.7**	0.3	3
	sporulation control protein Alr4153	651						
458E5	putative peptidase	876	**0.4**	0.2	3	**0.8**	0.2	3
NUM - nucleotide metabolism								

439D9	inosine monophosphate dehydrogenase-GMP reductase	798	**0.4**	0.1	4	**0.9**	0.3	3
	AT hook motif domain protein	663						
442F2	phosphoribosylformylglycinamidine synthase I PurQ	675	**0.4**	0.0	2	**1.4**	0.3	2
<u>REG - gene regulation</u>								
432G1	LtrC-like protein LtrC	452	**0.4**	0.3	2	**2.0**	0.2	2
	hypothetical protein Vng7115	409						
447A5	2-domain regulatory protein Prp2	1019	**0.5**	0.2	4	**1.0**	0.5	3
450G2	sugar-specific transcriptional regulator TrmB	1140	**0.4**	0.0	2	**0.7**	0.1	2
<u>RRR - replication, repair, recombination</u>								
440H6	DNA gyrase, A subunit GyrA	1421	**0.4**	0.1	3	**0.8**	0.3	3
460C5	DNA polymerase B PolB1	1523	**0.4**	0.1	3	**0.7**	0.1	3
<u>SIG - signal transduction</u>								
451B12	MCP domain signal transducer Htr4	1218	**0.5**	0.2	3	**1.1**	0.7	2
<u>TC - transcription</u>								
435H3	transcription factor B Tfb	958	**0.4**	0.1	4	**0.8**	0.2	3
456D3	transcription factor B Tfb	638	**0.4**	0.1	4	**1.0**	0.1	3
<u>TL - translation</u>								
435H4	translation initiation factor 2, subunit alpha Eif2A	801	**0.4**	0.1	4	**0.8**	0.3	3
448H7	ribosomal protein L24 RplX	399	**0.5**	0.1	2	**2.2**	1.0	2
	conserved hypothetical protein	396						
	hypothetical protein	396						
459F10	tyrosyl-tRNA synthetase TyrS	871	**0.4**	0.1	3	**0.7**	0.2	3
	lipase-esterase	608						
<u>TP - small molecule transport</u>								
436D4	dipeptide ABC transporter ATP-binding DppD	1535	**0.3**	0.1	4	**0.6**	0.3	3
438B3	ferrichrome-binding protein (Vng2549C)	797	**0.5**	0.4	4	**1.2**	0.6	2
439F6	ferrichrome-binding protein	1248	**0.2**	0.0	2	**2.0**	0.8	3
441H2	cationic amino acid transporter Cat-1	880	**0.5**	0.1	3	**1.4**	0.6	3
444H6	oligopeptide ABC transporter, solute-binding protein OppA	1412	**0.3**	0.1	4	**0.6**	0.2	3
	hypothetical protein	1359						
449H12	ABC transport protein inner membrane component	894	**0.5**	0.3	3	**2.2**	1.4	2
	ABC transport protein inner membrane component	544						
452D5	ABC transporter permease protein MacB	1143	**0.5**	0.1	4	**1.4**	0.5	3
455A2	amino acid ABC transporter (substrate-binding protein)	921	**0.3**	0.1	3	**0.7**	0.1	3
459H9	ferrichrome-binding protein (Vng2549C)	1115	**0.5**	0.2	3	**1.1**	0.3	3
<u>CHY - conserved hypothetical protein, HY - hypothetical protein, NOF - no function assigned to experimentally identified protein; no homolgy to annotated genes</u>								
434A7			**0.4**	0.3	3	**1.2**	0.1	3
434G7	homolog 1 to rad50 ATPase	1196	**0.5**	0.1	4	**0.8**	0.3	3
	homolog 1 to rad50 ATPase	1160						
434H4	homolog 1 to rad50 ATPase	1196	**0.5**	0.1	4	**1.0**	0.5	3
	homolog 1 to rad50 ATPase	1160						
436A5			**0.5**	0.2	3	**1.2**	0.7	3
436D3	hypothetical protein	972	**0.5**	0.3	4	**1.3**	0.9	3
	glucose dehydrogenase OxrA	786						
	sigma-70 region 4 like DNA binding protein	495						
	hypothetical protein	492						
	hypothetical protein	476						
441D2	membrane protein, putative	781	**0.4**	0.2	4	**1.0**	0.4	3
	precorrin-8W decarboxylase CbiT	620						
441H3	replication protein A	1213	**0.4**	0.1	4	**0.7**	0.2	3

442F5	conserved hypothetical protein	519	**0.4**	0.1	3	**0.9**	0.0	2
	sugar-specific transcriptional regulator TrmB	300						
443C7	hypothetical protein rrnB0067	448	**0.4**	0.2	2	**0.9**	0.3	3
	hypothetical protein rrnB0068	417						
	hypothetical protein rrnB0068	313						
444B11	hypothetical protein	780	**0.3**	0.2	3	**1.0**	0.4	3
	conserved protein	576						
	mannose-1-phosphate guanylyltransferase ManC	507						
	conserved hypothetical protein	402						
444F5	hypothetical protein	1536	**0.3**	0.0	3	**0.6**	0.4	3
	membrane protein Pan1	724						
	conserved hypothetical protein	555						
445F1			**0.5**	0.2	3	**1.2**	0.2	2
445H1	hypothetical protein Vng6400h	1078	**0.5**	0.2	4	**1.1**	0.4	2
446D1			**0.3**	0.1	4	**0.5**	0.1	3
448E7			**0.3**	0.4	2	**1.0**	0.2	3
450B1	predicted phospho-2-dehydro-3-deoxyheptonate aldolase	729	**0.5**	0.1	4	**0.9**	0.2	2
	tryptophan synthase, alpha subunit TrpA	511						
451F9	hypothetical protein	670	**0.5**	0.3	3	**1.2**	0.1	2
	endonuclease III NthA1	530						
455A10	circadian regulator	741	**0.5**	0.1	4	**1.0**	0.3	3
457B9	HD domain protein	813	**0.4**	0.1	3	**0.9**	0.1	2
	small conductance mechanosensitive ion channel	463						
458F2	conserved hypothetical protein	558	**0.4**	0.1	4	**0.8**	0.2	3
	conserved hypothetical protein	415						
	Hypothetical protein Vng6400h	309						
459C2	mRNA 3'-end processing factor Epf1	735	**0.2**	0.0	2	**0.6**	0.2	3
	geranylgeranyl reductase family subfamily	593						
460B2	conserved hypothetical protein	668	**0.4**	0.1	4	**1.0**	0.3	3
	serine hydroxymethyltransferase GlyA	516						
**Stationary growth phase overtranslation**								
CP - cellular processes								
435H5	protein kinase Prk	1185	**2.3**	2.1	3	**0.4**	0.1	3
444E6	kinase anchor protein PrkA	1167	**1.4**	0.9	2	**0.4**	0.1	3
EM - energy metabolism								
450E9	halocyanin precursor-like HcpB	687	**0.9**	0.2	2	**0.2**	0.2	2
	natural resistance-associated macrophage protein family	342						
MIS - miscellaneous								
433F2	glycosyltransferase	907	**2.3**	1.4	3	**0.5**	0.1	3
	stage V sporulation protein R-like SpoVR	542						
442E3	urease, alpha subunit UreC	1075	**0.9**	0.4	3	**0.4**	0.2	3
CHY - conserved hypothetical protein, HY - hypothetical protein, NOF - no function assigned to experimentally identified protein; no homolgy to annotated genes								
443E2	stage V sporulation protein R-like SpoVR	956	**1.2**	0.9	2	**0.4**	0.1	3
	glycosyltransferase	588						
444D11	stage V sporulation protein R-like SpoVR	713	**1.1**	0.5	3	**0.5**	0.2	3
	glycosyltransferase	619						
456A7	hypothetical protein	1440	**0.8**	0.2	3	**0.5**	0.2	3
	lipoprotein, putative	1058						
	lipoprotein, putative	441						

[*1]All genes are tabulated which have a “translational repression factor” of ≤0.5 differentially in exponential phase (upper part) or in stationary phase (lower part). The translational repression factor is the quotient of free RNA to polysome-bound RNA (normalized to the average of all genes). The identifier is the clone designation in the onefold coverage genome library used to generate the shotgun DNA microarray (Zaigler et al. 2003). The “putative functions” are derived from blast searches against public databases. The “length” is the partial or total length of the gene which is present in the respective clone. The columns ∅, SD and n list average value, standard deviation and number of biological replicates included in the analysis.
