# Supplementary material for: Genome-wide analysis of growth phase-dependent translational and transcriptional regulation in halophilic archaea
Source: BMC Genomics. 2007 Nov 12;8:415. doi: 10.1186/1471-2164-8-415 (PMC3225822; doi:10.1186/1471-2164-8-415)
Supplement: Additional file 5 — H. volcanii genes with constitutive non-average translational efficiency [file 1471-2164-8-415-S5.pdf]

Table S5. *H. volcanii* genes with constitutive non-average translational efficiency[*1]

identifier	putative function	length (bp)	translational repression factor exp ∅	exp SD	exp n	stat ∅	stat SD	stat n
**Constitutive undertranslation**								
AA - amino acid metabolism								
436G3	branched-chain amino acid aminotransferase IlvE1	512	**3.3**	0.8	4	**2.9**	1.4	3
	rieske [2Fe-2S] domain containing oxidoreductase	426						
	GtrA-like family protein	320						
COM - coenzyme metabolism								
457D5	NAD+ synthetase NadE	480	**2.8**	1.8	3	**2.1**	0.5	3
ISH - transposases and ISH-encoded proteins								
435B10	transposase Tnp	999	**2.6**	0.4	4	**3.0**	1.1	3
435E12	transposase Tnp	519	**3.8**	0.9	3	**2.5**	0.9	3
	tryptophanyl-tRNA synthetase TrpS	468						
438E1	transposase Tnp	999	**3.6**	1.2	4	**4.8**	0.8	3
447C11	transposase Tnp	287	**2.4**	1.1	2	**3.2**	1.3	3
449G12	transposase Tnp	372	**3.2**	1.1	2	**3.8**	2.7	2
452F5	transposase Tnp	999	**3.0**	0.6	4	**4.0**	1.7	3
453G11	transposase Tnp	384	**4.2**	2.9	4	**3.5**	0.9	3
454C9	transposase Tnp	537	**3.3**	1.0	4	**4.3**	1.1	3
457D3	transposase	324	**2.8**	1.6	3	**2.3**	0.8	3
MIS - miscellaneous								
432H4	conditioned medium-induced protein 9-related protein	288	**2.9**	1.3	4	**3.1**	1.3	3
	conserved hypothetical protein	224						
	cold shock transcription regulator protein CspD1	195						
438F2	nonhistone chromosomal protein MC1	237	**6.6**	0.5	2	**4.9**	0.8	3
	dihydroorotate oxidase PyrD	186						
447D4	conditioned medium-induced protein 9-related protein	288	**3.0**	1.1	4	**4.7**	2.2	3
	cold shock transcription regulator protein CspD1	192						
448A8	rhodanese-like domain protein	366	**2.2**	0.9	4	**2.8**	1.8	3
	translation initation factor SUI1, putative	294						
	hypothetical protein	221						
TP - small molecule transport								
439E10	multi antimicrobial extrusion drug-sodium antiporter MatE	518	**3.7**	1.4	2	**2.9**	0.8	3
CHY - conserved hypothetical protein, HY - hypothetical protein, NOF - no function assigned to experimentally identified protein; no homolgy to annotated genes								
431E9	hypothetical protein	949	**5.8**	1.1	4	**5.7**	2.2	3
	transposase Cap8H	840						
431G4	conserved hypothetical protein	613	**3.9**	2.9	2	**2.3**	1.1	3
	hypothetical protein	351						
432B7	cyanoglobin GlbN	363	**4.0**	1.5	4	**3.3**	2.3	3
	putative transcription factor TFIIB repeat	230						
432C2			**8.0**	3.7	4	**13.7**	10.7	3
432E2			**13.3**	4.4	4	**16.2**	7.2	3
432F1			**8.9**	4.8	4	**13.8**	8.7	3
433A2	hypothetical protein	560	**14.3**	13.9	4	**8.0**	3.2	3
	transposase Cap8H	443						

434D8	conserved protein	1314	**7.7**	4.5	3	**9.5**	10.9	2
435E2	hypothetical protein	1723	**3.0**	1.0	4	**2.7**	1.3	3
	acyl-CoA thioester hydrolase Act	483						
	conserved hypothetical protein	456						
	uncharacterized ACR, COG1259 superfamily	444						
436C6	hypothetical protein	1049	**9.6**	1.8	4	**11.8**	10.0	3
	dihydrofolate reductase FolA	419						
436E3	hypothetical protein Vng6148h	816	**2.6**	0.6	3	**2.3**	0.3	3
436H11	hypothetical protein Vng6148h	842	**2.1**	0.4	3	**2.2**	0.0	3
	SNF2 family N-terminal domain protein	540						
438H5	conserved hypothetical protein	386	**4.0**	2.9	4	**7.2**	5.1	3
441D12	conserved protein	318	**4.5**		2	**3.1**	0.9	2
443B3	hypothetical protein Vng6406H	931	**3.1**	0.1	2	**2.1**	0.2	3
443C3			**4.6**	1.1	3	**3.8**	1.2	3
446H9			**9.0**	3.4	4	**12.1**	6.2	3
447A6	membrane protein, putative	883	**2.5**	1.1	2	**2.9**	1.8	3
447B12	hypothetical protein	277	**19.0**	10.0	4	**23.1**	20.1	3
	transposase Cap8H	160						
448E3			**10.9**	9.8	4	**6.0**	3.9	3
448F9			**24.8**	18.0	4	**36.6**	24.9	3
450H8	conserved hypothetical protein	414	**3.6**	3.0	4	**2.6**	1.6	3
	3-ketoacyl-CoA thiolase AcaB2	394						
451C1	hypothetical protein	1691	**8.6**	1.3	3	**9.3**	4.2	2
452E9			**6.4**	4.5	4	**12.8**	10.6	3
453F7			**12.2**	3.4	4	**17.7**	9.9	3
456E3	conserved hypothetical protein	537	**3.8**	0.4	3	**4.2**	1.3	3
459B12	conserved hypothetical protein	648	**2.1**	1.0	4	**3.1**	1.7	3
**Constitutive overtranslation**								
AA - amino acid metabolism								
438C6	3-isopropylmalate dehydratase, large subunit LeuC	1252	**0.4**	0.1	4	**0.3**	0.2	3
	3-isopropylmalate dehydratase, small subunit LeuD	609						
440H4	aspartate aminotransferase AvtA4	1041	**0.4**	0.0	4	**0.4**	0.2	3
457A1	beta-eliminating lyase TnaA	1407	**0.3**	0.1	4	**0.5**	0.1	3
457E2	glutamate synthase large subunit GltA	1699	**0.3**	0.1	4	**0.4**	0.1	3
CHM - carbohydrate metabolism								
437A5	L-lactate dehydrogenase	510	**0.4**	0.1	4	**0.5**	0.1	3
	ferritin-like domain subfamily	453						
440F5	glucoamylase	877	**0.3**	0.1	3	**0.4**	0.3	3
454E2	NAD dependent epimerase-dehydratase family	1026	**0.4**	0.1	2	**0.4**	0.0	3
CIM - central intermediary metabolism								
432H8	aldehyde ferredoxin oxidoreductase Aor2	1042	**0.5**	0.1	4	**0.4**	0.1	2
437H9	2-oxoglutarate ferredoxin oxidoreductase subunit alpha	1217	**0.3**	0.1	4	**0.3**	0.1	3
438C2	pyruvate dehydrogenase E1 alpha subunit PdhA2	816	**0.4**	0.1	4	**0.5**	0.2	3
	pyruvate dehydrogenase PdhB	505						
439F10	succinate dehydrogenase flavoprotein subunit SdhA	1148	**0.3**	0.1	4	**0.5**	0.2	3
444C5	2,3-bisphosphoglycerate-independent PGA mutase	1645	**0.4**	0.1	3	**0.5**	0.2	3
445F10	acetate-CoA ligase AcsA	760	**0.5**	0.1	4	**0.5**	0.2	3
	bacterio-opsin activator-like protein Boa	711						
446B3	succinate dehydrogenase iron-sulfur protein SdhB	661	**0.3**	0.0	4	**0.4**	0.0	3
449A6	glycolate oxidase subunit GlcD	1061	**0.5**	0.2	4	**0.5**	0.2	3
450C10	acetate--CoA ligase AcsA	438	**0.4**	0.0	2	**0.5**	0.1	3

	bacterio-opsin activator-like protein Boa	247						
	acyl-coenzyme A synthetase ACS5	222						
457C3	aconitate hydratase 1 AcnA	509	**0.3**	0.1	4	**0.4**	0.2	3
	thioredoxin	261						
458A10	pyruvate dehydrogenase PdhB	492	**0.4**	0.2	4	**0.4**	0.2	3
	pyruvate dehydrogenase E1 alpha subunit PdhA2	294						
460E10	succinyl-Coa synthetase Acs3	961	**0.4**	0.1	4	**0.4**	0.1	3
	cobyrinic acid ac-diamide synthase Pta	682						
COM - coenzyme metabolism								
447G2	precorrin-3B C17-methyltransferase CobH	746	**0.3**	0.1	4	**0.5**	0.4	3
431B8	cobalamin biosynthesis CbiL	540	**0.3**	0.1	3	**0.4**	0.1	3
	precorrin-8W decarboxylase CbiT	506						
	cobalamin biosynthesis precorrin-3 methylase CbiF	294						
CP - cellular processes								
446A11	general stress protein 69 Gsp	963	**0.4**	0.1	4	**0.5**	0.1	3
452G11	cell division control protein 48 Cdc48	1118	**0.3**	0.1	4	**0.4**	0.2	3
	hypothetical protein RrnAC1529	579						
EM - energy metabolism								
439A6	V-type ATPase, subunit A	1210	**0.4**	0.0	4	**0.4**	0.1	3
444H1	V-type ATPase, subunit A	736	**0.4**	0.1	4	**0.4**	0.1	3
	V-type ATPase, subunit C	557						
448F1	V-type ATPase, subunit A	809	**0.3**	0.1	4	**0.4**	0.1	3
448G5	V-type ATPase, subunit A	1461	**0.3**	0.1	4	**0.3**	0.1	3
449E6	V-type ATPase, subunit A	918	**0.4**	0.1	4	**0.3**	0.2	3
LIP - lipid metabolism								
454A12	3-hydroxybutyryl-CoA dehydrogenase HBD2	1159	**0.4**	0.1	4	**0.5**	0.3	3
MIS - miscellaneous								
432H9	sulfatase arylsulfatase A-like	937	**0.4**	0.1	4	**0.5**	0.1	3
	glutamate-cysteine ligase family 2 GCS2	462						
436A2	N-acyl-L-amino acid amidohydrolase	920	**0.4**	0.1	4	**0.4**	0.2	3
	hypothetical protein	887						
	oligopeptide ABC transporter peptide-binding protein	688						
440B1	cobalamin biosynthesis CbiG	632	**0.3**	0.1	4	**0.3**	0.1	3
	precorrin-3B C17-methyltransferase CobH	528						
441B2	phenylacetic acid degradation protein PaaA	683	**0.3**	0.1	4	**0.4**	0.1	3
447H5	4-aminobutyrate aminotransferase GabT	629	**0.5**	0.2	3	**0.5**	0.1	3
	mRNA 3'-end processing factor-like Epf2	502						
448C2	catalase-peroxidase HPI KatG	480	**0.3**	0.1	4	**0.4**	0.1	3
450C2	mandelate racemase - muconate lactonizing enzyme	975	**0.3**	0.1	4	**0.4**	0.1	3
451A6	mandelate racemase - muconate lactonizing enzyme, N-terminal domain protein	1062	**0.3**	0.1	4	**0.4**	0.2	3
452B2	hydantoinase	1375	**0.3**	0.1	4	**0.3**	0.1	3
452C2	phenylacetic acid degradation protein PaaA	852	**0.3**	0.1	4	**0.3**	0.1	3
454A2	aldehyde ferredoxin oxidoreductase Aor	1643	**0.3**	0.1	2	**0.3**	0.0	3
455G2	4-aminobutyrate aminotransferase GabT	1326	**0.4**	0.2	3	**0.5**	0.1	3
455H3	catalase-peroxidase HPI KatG	713	**0.3**	0.1	3	**0.4**	0.1	3
	N5,N10-methylenetetrahydromethanopterin reductase	647						
456A8	hydantoinase	474	**0.5**	0.2	4	**0.4**	0.2	3
458G9	phenylacetic acid degradation protein PaaA	564		0.2	4	**0.3**	0.1	3
	transcription regulator Boa-12	441						
459G4	cobalamin biosynthesis CbiG	981	**0.4**	0.1	4	**0.3**	0.1	3

RRR - replication, repair, recombination								
434D1	DNA topoisomerase VI, B subunit Top6B	1539	**0.3**	0.1	4	**0.2**	0.0	3
440A10	excinuclease ABC, B subunit UvrB	1323	**0.5**	0.2	3	**0.5**	0.0	3
440G11	DNA polymerase B PolB1	1359	**0.4**	0.1	4	**0.4**	0.2	3
448H1	DNA topoisomerase I TopA	587	**0.4**	0.1	3	**0.5**	0.1	3
455A6	DNA gyrase, B subunit GyrB	856	**0.3**	0.1	4	**0.3**	0.1	3
	DNA topoisomerase VI, B subunit Top6B	542						
457G12	excinuclease ABC, B subunit UvrB	1487	**0.4**	0.1	3	**0.5**	0.2	3
SIG - signal transduction								
439G2	HTR-like protein HtlD	1492	**0.5**	0.1	3	**0.5**	0.1	3
455G7	heme-based aerotactic transducer HemAT Htr10	571	**0.5**	0.1	4	**0.4**	0.2	3
TC - transcription								
433F10	DNA-directed RNA polymerase subunit B RpoB2	1566	**0.4**	0.1	4	**0.3**	0.1	3
438E9	DNA-directed RNA polymerase RpoE	573	**0.3**	0.1	4	**0.4**	0.1	3
444B9	DNA-directed RNA polymerase, subunit A'' RpoA2	1029	**0.5**	0.1	4	**0.4**	0.1	3
	DNA-directed RNA polymerase subunit A' RpoA1	799						
445H3	DNA-directed RNA polymerase subunit A' RpoA1	987	**0.3**	0.1	4	**0.5**	0.3	3
	DNA-directed RNA polymerase beta subunit RpoB	440						
447C10	DNA-directed RNA polymerase RpoE	569	**0.4**	0.1	4	**0.4**	0.2	3
	translation initiation factor eIF-2 subunit gamma Eif2G	511						
	predicted RNA-binding protein containing PIN domain	390						
460G3	DNA-directed RNA polymerase subunit B RpoB2	1528	**0.3**	0.1	4	**0.4**	0.0	3
TL - translation								
433D2	isoleucyl-tRNA synthetase IleS	1113	**0.4**	0.1	4	**0.4**	0.1	3
436E1	ribosomal protein L22 RplV	462	**0.3**	0.1	4	**0.3**	0.2	3
	ribosomal protein S19 RpsS	423						
	ribosomal protein L2 RplB	359						
440C3	translation initiation factor eIF-2 subunit gamma Eif2G	1034	**0.3**	0.1	4	**0.4**	0.1	3
446A3	ribosomal protein L14p Rpl14P	372	**0.4**	0.1	4	**0.5**	0.4	3
446E8	ribosomal protein L32E Rpl32e	672	**0.4**	0.1	4	**0.5**	0.3	3
	ribosomal protein L19e	385						
451H10	leucyl-tRNA synthetase LeuS	1431	**0.3**	0.1	4	**0.4**	0.1	3
	hypothetical protein	1431						
455D12	ribosomal protein L6p Rpl6p	537	**0.4**	0.1	4	**0.4**	0.2	3
	ribosomal protein S8 RpsH	393						
	ribosomal protein L32E Rpl32e	367						
460C6	phenylalanyl-tRNA synthetase alpha chain PheS	1334	**0.4**	0.2	3	**0.3**	0.0	2
TP - small molecule transport								
432D5	dipeptide ABC transporter ATP-binding DppF	1274	**0.4**	0.0	4	**0.4**	0.1	3
438H12	oligopeptide ABC transporter Mll9136, peptide-binding	988	**0.4**	0.1	4	**0.4**	0.2	3
	dipeptide transport system permease protein Mll9135	510						
439C3	dipeptide transport system permease protein Mll9134	900	**0.4**	0.1	4	**0.4**	0.1	3
	dipeptide transport system permease protein Mll9135	739						
	hypothetical protein	543						
444G10	dipeptide ABC transporter ATP-binding DppD	1245	**0.4**	0.2	4	**0.4**	0.1	3
449H2	oligopeptide ABC transporter peptide-binding protein	748	**0.3**	0.1	4	**0.3**	0.2	3
	dipeptide ABC transporter permease DppB2	561						
455F7	oligopeptide ABC transporter Mll9136, peptide-binding	1516	**0.3**	0.0	4	**0.4**	0.2	3
455H8	dipeptide transport system permease protein Mll9135	918	**0.4**	0.1	4	**0.4**	0.2	3
	oligopeptide ABC transporter Mll9136, peptide-binding	514						
456A3	dipeptide transport system permease protein Mll9135	815	**0.3**	0.0	4	**0.4**	0.1	3

460H7	oligopeptide ABC transporter peptide-binding protein	753	**0.3**	0.0	4	**0.4**	0.2	3
	dipeptide ABC transporter permease DppB2	502						
CHY - conserved hypothetical protein, HY - hypothetical protein, NOF - no function assigned to experimentally identified protein; no homolgy to annotated genes								
435C3	protein of unknown function (DUF917) superfamily	1089	**0.4**	0.1	4	**0.4**	0.1	3
	protein of unknown function (DUF917) superfamily	518						
439A9	hypothetical protein	714	**0.4**	0.1	4	**0.4**	0.2	3
	L-carnitine dehydratase/bile acid-inducible protein F	671						
446D1			**0.3**	0.1	4	**0.5**	0.1	3
446H2	mRNA 3'-end processing factor-like Epf2	965	**0.3**	0.0	4	**0.4**	0.1	3
448G7			**0.3**	0.1	2	**0.5**	0.1	3
450A5	hypothetical protein rrnAC1828	1209	**0.4**	0.1	4	**0.5**	0.2	3
	FeS assembly protein SufB	418						
452G2	endonuclease Staphylococcal nuclease-like ComA	820	**0.4**	0.1	2	**0.5**	0.2	3
452H12			**0.3**	0.2	2	**0.4**	0.0	2
453C4	hypothetical protein rrnAC1529	762	**0.4**	0.1	4	**0.4**	0.3	3
	cell division control protein 48 Cdc48	593						
455E4			**0.5**	0.1	4	**0.5**	0.2	3
459A5	oxidoreductase MviM3	948	**0.4**	0.3	3	**0.5**	0.3	3
	conserved hypothetical protein	670						

[*1]All genes are tabulated which have a “translational repression factor” of ≤0.5 or ≥2.0 in exponential phase and in stationary phase. The translational repression factor is the quotient of free RNA to polysome-bound RNA (normalized to the average of all genes). The identifier is the clone designation in the onefold coverage genome library used to generate the shotgun DNA microarray (Zaigler et al. 2003). The “putative functions” are derived from blast searches against public databases. The “length” is the partial or total length of the gene which is present in the respective clone. The columns ∅, SD and n list average value, standard deviation and number of biological replicates included in the analysis.
