# Supplementary material for: Genome-wide analysis of growth phase-dependent translational and transcriptional regulation in halophilic archaea
Source: BMC Genomics. 2007 Nov 12;8:415. doi: 10.1186/1471-2164-8-415 (PMC3225822; doi:10.1186/1471-2164-8-415)
Supplement: Additional file 6 — Oligonucleotides used in the quantitative Realtime PCR analysis [file 1471-2164-8-415-S6.pdf]

**Table S6. Oligonucleotides used in the quantitative Realtime PCR analysis (in 5'→3' direction)**

| ORF | 5' oligonucleotide | 3' oligonucleotide |
|---|---|---|
| OE2010R | ATGAGCGACCGCGTTCGGGA | CGTGCAGCGTGGAACACGAG |
| OE2055F | TAGACTCCGAGCCGGAGCTG | TTGATGCCGACGACCGCGAC |
| OE2563R | GGTCGCTGCGAACCAGTGTC | TGCCGTCGTGGACCGAAACG |
| OE2595F | TCAGCAGACGCTTGCCGACC | AACCCGAGCAGTGCGTCGG |
| OE3637R | GGTCCTCGAACGCGAAACCG | TGGTGGCCATCGGGAGGTAG |
| OE4674F | CACCACGAGGACCGCCTC | ACGTAACCGACGGTGCCG |
| OE4511R | GCGTCGAAGAGATGCATCAG | AGTACAACAGGGTACTTCGG |
| OE3538R | ATGTCACAGGATTATCGTCTC | TCACTCGACGTACGCCACG |
| OE4187R | ATGGCAGACCTCATCGTCAAG | TTACAGGTCGCGGGGCTG |
| OE1119F | ATGGGTATCGGTTCTCCGAC | CTACCGATAGTACGGAATCG |
| OE3049R | CAACGACACGATCACGCTG | TCTCGCCGTCGGCACTG |
| OE1405R | TGACCGTCCTCGGCGAGGAAG CCCGATACG | ACGACCGCGTCGAAGCCCTCG TTGGTGAGC |
| OE3470F | CACGACGGCGGGAACCACGTG ACGCTGCAG | CGGAGCTGATCGGCGTCCTGG TCGGAGTAC |
| OE5071F | CAGCCATCGATGCCGATTC | TCCACTCCCAGCATCGAAC |
| OE4136R | CATGCTCGGCGCGAACCGCGT GCAGGTGCG | CGGAGCTGGTCGGCGTCGCTT TTCTCGTAG |
